# mTOR activation induces endolysosomal remodeling and nonclassical secretion of IL-32 via exosomes in inflammatory reactive astrocytes

**DOI:** 10.1186/s12974-024-03165-w

**Published:** 2024-08-08

**Authors:** Kun Leng, Brendan Rooney, Frank McCarthy, Wenlong Xia, Indigo V. L. Rose, Sophie Bax, Marcus Chin, Saeed Fathi, Kari A. Herrington, Manuel Leonetti, Aimee Kao, Stephen P. J. Fancy, Joshua E. Elias, Martin Kampmann

**Affiliations:** 1 Institute for Neurodegenerative Diseases, University of California, San Francisco, San Francisco, CA USA; 2 Biomedical Sciences Graduate Program, University of California, San Francisco, San Francisco, CA USA; 3 Medical Scientist Training Program, University of California, San Francisco, San Francisco, CA USA; 4 Chan Zuckerberg Biohub, San Francisco, CA USA; 5 Departments of Neurology and Pediatrics, School of Medicine, University of California, San Francisco, CA USA; 6 Neuroscience Graduate Program, University of California, San Francisco, San Francisco, CA USA; 7 Memory and Aging Center, Department of Neurology, University of California, San Francisco, San Francisco, CA USA; 8 Small Molecule Discovery Center, Department of Pharmaceutical Chemistry, University of California, San Francisco, San Francisco, CA USA; 9 Center for Advanced Microscopy, University of California, San Francisco, San Francisco, CA USA; 10 Weill Institute for Neurosciences, University of California, San Francisco, San Francisco, CA USA; 11 Department of Biochemistry and Biophysics, University of California, San Francisco, San Francisco, CA USA

**Keywords:** Astrocytes, Inflammatory reactive astrocytes, Neuroinflammation, mTOR, Endolysosomal system, IL-32, Extracellular vesicles

## Abstract

Astrocytes respond and contribute to neuroinflammation by adopting inflammatory reactive states. Although recent efforts have characterized the gene expression signatures associated with these reactive states, the cell biology underlying inflammatory reactive astrocyte phenotypes remains under-explored. Here, we used CRISPR-based screening in human iPSC-derived astrocytes to identify mTOR activation a driver of cytokine-induced endolysosomal system remodeling, manifesting as alkalinization of endolysosomal compartments, decreased autophagic flux, and increased exocytosis of certain endolysosomal cargos. Through endolysosomal proteomics, we identified and focused on one such cargo–IL-32, a disease-associated pro-inflammatory cytokine not present in rodents, whose secretion mechanism is not well understood. We found that IL-32 was partially secreted in extracellular vesicles likely to be exosomes. Furthermore, we found that IL-32 was involved in the polarization of inflammatory reactive astrocyte states and was upregulated in astrocytes in multiple sclerosis lesions. We believe that our results advance our understanding of cell biological pathways underlying inflammatory reactive astrocyte phenotypes and identify potential therapeutic targets.

**Supplementary Information:**

The online version contains supplementary material available at 10.1186/s12974-024-03165-w.

## Introduction

Astrocytes maintain homeostasis of the central nervous system in myriad ways, for example through phagocytosis of synapses [1, 2] or exocytosis of ATP or glutamate [3–5]. In the context of central nervous system injury or disease, astrocytes respond to and amplify inflammatory signaling cascades [6, 7], adopting inflammatory reactive astrocyte states characterized by distinct gene expression and cytokine signatures [8–10]. Although recent efforts have elucidated these signatures, the cell biological pathways underlying inflammatory reactive astrocyte phenotypes remain under-explored.

The endolysosomal system encompasses a diverse pool of intracellular vesicles of varying luminal pH that mediate degradative (e.g. lysosomes, phagosomes, autophagosomes) as well as exocytic functions (e.g. recycling endosomes, multivesicular bodies) [11, 12]. Although a large body of work exists on the cross-regulation of autophagy and inflammatory responses in immune cells [13], much less is known about how this occurs in brain cell types, especially astrocytes.

Here, using human induced pluripotent stem cell (hiPSC)-derived astrocytes, we found that the inflammatory cytokines IL-1α + TNF + C1q in combination (hereafter abbreviated as “ITC”), which have been widely used to induce inflammatory astrocyte reactivity in vitro [14], caused mTOR activation and mTOR-dependent remodeling of the endolysosomal system, which was associated with increased exocytosis of certain endolysosomal cargos. Through endolysosomal proteomics, we identified and focused on one such cargo, the pro-inflammatory cytokine IL-32. Although cerebrospinal fluid levels of IL-32 are elevated in neuroinflammatory diseases such as multiple sclerosis [15], the secretion mechanism of IL-32 is not well understood, as it lacks a classical signal peptide [16, 17]. Furthermore, it is unclear what cell types in the central nervous system produce IL-32 under neuroinflammatory conditions. We found that IL-32 was upregulated in astrocytes in multiple sclerosis lesions, was likely secreted in part via exosomes, and was involved in the polarization of inflammatory reactive astrocyte states.

## Results

**Fig. 1:**
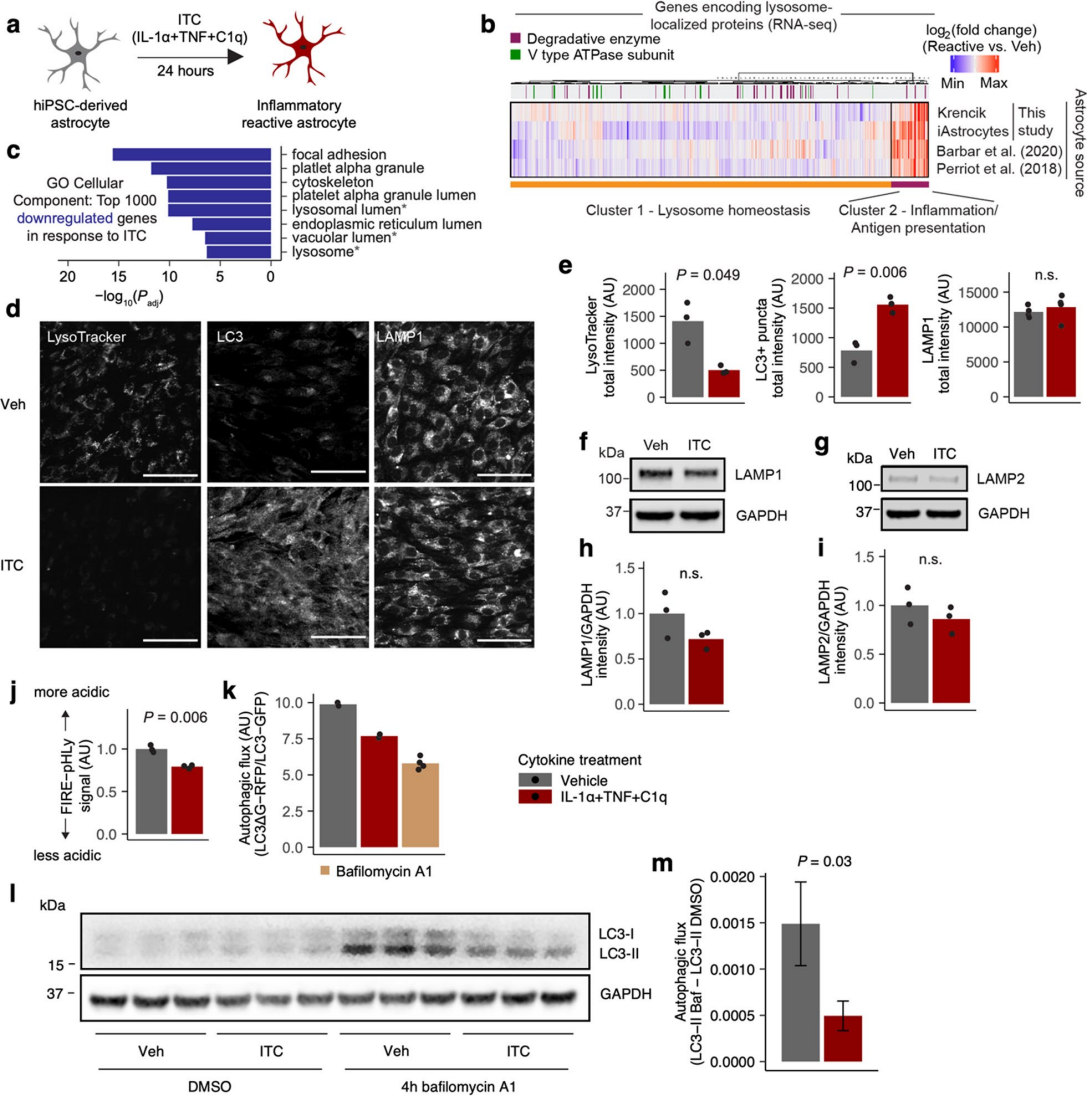
Endolysosomal function is perturbed in inflammatory reactive astrocytes. **a** Schematic of modeling inflammatory astrocyte reactivity in vitro. **b** Heatmap of changes in the expression of genes encoding lysosome-localized proteins in different hiPSC-derived astrocyte models treated with ITC or similar treatments vs vehicle control. **c** Top GO Cellular Component terms enriched among the top 1000 downregulated genes in iAstrocytes treated with ITC; endolysosomal system-related terms are highlighted by asterisks. **d** Representative images of LysoTracker staining in live iAstrocytes or immunostaining of LC3 or LAMP1 in fixed and permeabilized iAstrocytes; scale bar = 75 μm. **e** Quantification of imaging experiments shown in** d** (n = 3 wells per condition). **f**, **g** Representative immunoblot bands against LAMP1 (**f**) or LAMP2 (**g**). **h**, **i** Quantification of immunoblot experiments shown in **f**, **g** (n = 3 wells per condition). **j** Measurement of the acidity of LAMP1^+^ endolysosomal compartments using FIRE-pHLy via flow cytometry (see Methods, Additional file 8). **k** Measurement of autophagic flux with the LC3ΔG-RFP/LC3-GFP fluorescent reporter from Kaizuka et al. [25]. **l** Immunoblot against LC3 demonstrating LC3-I and LC3-II bands. **m** Quantification of autophagic flux from the LC3-II bands in **l**; error bars reflect the 95% confidence interval associated with the standard error of the mean; individual data points not shown because the quantities of interest are differences between means, with no biologically meaningful pairing of individual data points across conditions. *P* values where shown were calculated using two-sided Student’s t test

To identify the cellular pathways responsible for the loss of homeostatic functions that accompanies inflammatory astrocyte reactivity [14], we reanalyzed RNA-sequencing data from multiple hiPSC-derived astrocyte models [18–22] (Table S1, Additional file 3) treated with ITC or similar treatments (Fig. 1a), focusing on downregulated genes. We found that downregulated genes after ITC treatment were enriched in genes encoding proteins associated with the endolysosomal system (Fig. 1b, c, Supplementary Fig. 1), suggesting a potential link to the reported phenotype of decreased phagocytosis.

To confirm if ITC indeed perturbed endolysosomal system function, we performed downstream experiments using the iAstrocyte model that we previously developed (WTC11 genotype) [9]. We found that ITC reduced LysoTracker staining without appreciably changing total endolysosomal mass as measured by the abundance of LAMP1 or LAMP2 (Fig. 1d–i, Additional file 1), which tend to be enriched in lysosomes but can also mark other endolysosomal compartments [23]. Since a decrease in LysoTracker staining can reflect a decrease in either total endolysosomal mass or acidity, we deduced from the above results that ITC caused endolysosomal alkalinization. Indeed, using a genetically encoded endolysosomal pH reporter (FIRE-pHLy) [24], we found that ITC decreased the acidity of LAMP1^+^ endolysosomal compartments (Fig. 1j). We also observed a buildup of LC3^+^ puncta on immunostaining after ITC (Fig. 1d, e), suggestive of perturbed autophagic flux. Using both a genetically encoded reporter [25] (Fig. 1k) and LC3-II western blot [26] (Fig. 1l, m, Additional file 1), we found that ITC decreased autophagic flux as well, consistent with the importance of acidic lysosomes for degradation of autophagic substrates [27].

**Fig. 2:**
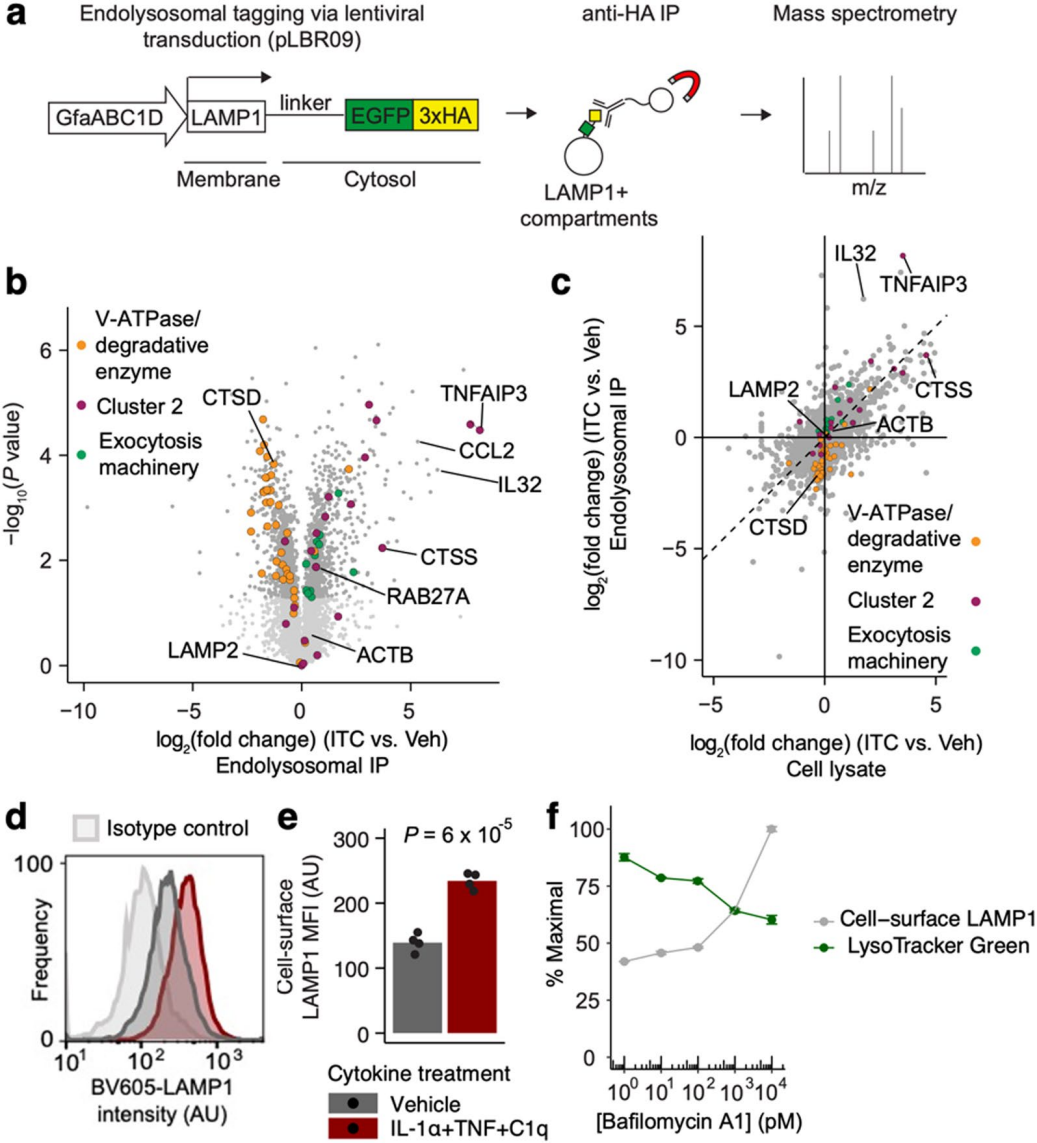
Perturbation of endolysosomal function is accompanied by remodeling of the endolysosomal proteome. **a** Schematic of endolysosomal proteomics workflow (n = 3 wells per condition). **b** Volcano plot of endolysosomal proteomic data. **c** Scatterplot comparing log_2_-fold change of endolysosomal vs total cell lysate protein abundance in ITC-treated iAstrocytes compared to vehicle-treated iAstrocytes (IP: immunoprecipitation). **d** Representative histograms of cell-surface LAMP1 staining intensity in vehicle- or ITC-treated iAstrocytes compared to isotype control staining intensity measured by flow cytometry. **e** Median fluorescence intensity (MFI) of cell-surface LAMP1 measured by flow cytometry (n = 3 wells per condition); *P* value by two-sided Student’s t test. **f** Cell-surface LAMP1 or LysoTracker staining in iAstrocytes treated with increasing doses of bafilomycin A1 (n = 3 wells per condition; error bars reflect the standard error of the mean)

To gain a more detailed understanding of how endolysosomal function is perturbed in inflammatory reactive astrocytes, we performed mass spectrometry-based proteomic characterization of LAMP1^+^ endolysosomal compartments in ITC-treated vs control iAstrocytes (Fig. 2a, see Methods, Additional file 8), using the total cell lysate as a reference. We found that v-ATPase subunits and degradative enzymes were less abundant in endolysosomal compartments in ITC-treated iAstrocytes (Fig. 2b, c), consistent with transcript-level downregulation in the RNA-seq data (Fig. 1b). Furthermore, CST3, a potent inhibitor of lysosomal proteases [28], accumulated in endolysosomal compartments upon ITC treatment (Table S2, Additional file 4). We also detected endolysosomal accumulation of proteins involved in vesicular exocytosis (e.g. RAB27A [29], SNAP25 [3, 5]) together with inflammatory mediators (e.g. IL-32 [16, 17], CCL2 [30]), suggesting that ITC may cause rerouting of endolysosomal trafficking to facilitate nonclassical secretion of inflammatory factors.

To explore this hypothesis, we developed a flow-cytometric assay to quantify in live unpermeabilized iAstrocytes the exposure of the LAMP1 luminal domain on the plasma membrane (see Methods, Additional file 8), which is known to increase in degranulating immune cells [31] and may be useful as a marker of the exocytic activity of certain endolysosomal pathways [32]. With this approach, we found that ITC-treated iAstrocytes had ~ 1.8 times the amount of cell-surface LAMP1 relative to vehicle-treated controls, despite equivalent levels of total LAMP1 (Fig. 2d, e). We were able to directly observe putative endolysosomal exocytic events from LAMP1^+^ vesicles via total internal reflection fluorescence microscopy of iAstrocytes transduced with a LAMP1-mCherry fusion construct and loaded with LysoTracker Green, where the green fluorescence from LysoTracker was lost from LAMP1-mCherry^+^ vesicles trafficked to the plasma membrane (Additional file 2). Furthermore, the abundance of cell-surface LAMP1 demonstrated a positive dose–response relationship with bafilomycin A1 (Fig. 2f), an inhibitor of v-ATPase activity and autophagosome-lysosome fusion [33] that is known to increase endolysosomal exocytosis [34–36]. Compared to cell-surface LAMP1, LysoTracker staining demonstrated a negative dose–response relationship (Fig. 2f), likely reflecting alkalinization of endolysosomal compartments due to v-ATPase inhibition.

**Fig. 3:**
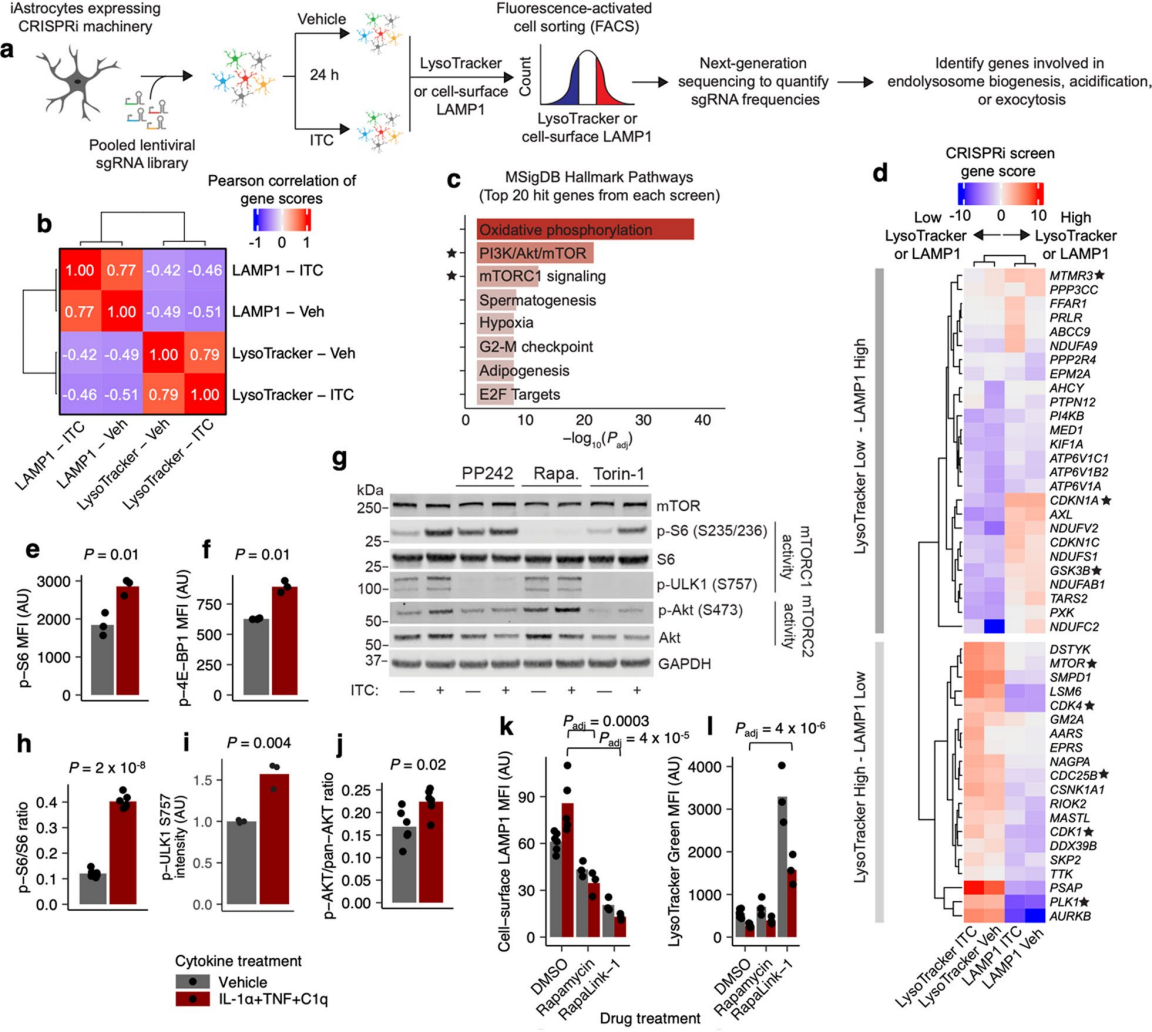
Multi-phenotypic CRISPR-based screening identifies mTOR as a central regulator of endolysosomal system function. **a** Schematic of CRISPR-based screening workflow. **b** Pearson correlation of gene scores (see Methods, Additional file 8) of hits from the LAMP1 vs LysoTracker screens (n = 2 biological replicate screens per condition). **c** Enrichment analysis against MSigDB Hallmark Pathways terms of the top 20 hits from each screen; terms pertaining to mTOR are highlighted with stars. **d** Heatmap of gene scores of the hits overlapping with the highlighted MSigDB terms in **c**. **e**, **f** Median fluorescence intensity (MFI) of phospho-S6 (**e**) or phospho-4E-BP1 (**f**) staining in ITC- vs. vehicle-treated iAstrocytes measured by flow cytometry. **g** Representative immunoblot bands corresponding to mTOR, phospho-S6, total S6, phospho-ULK1, and phospho-AKT1 in ITC- vs vehicle-treated iAstrocytes in the presence of different mTOR inhibitors. **h** Quantification of immunoblotting experiments (n = 6 wells per condition for phospho-S6/S6, n = 3 wells per condition for phospho-ULK1, n = 6 wells per condition for phospho-AKT1/pan-AKT; *P* values from two-sided Student’s t test). **k**, **l** Cell-surface LAMP1 (**k**) or LysoTracker (**l**) MFI in ITC-vs. vehicle-treated iAstrocytes in the presence of mTOR inhibitors measured by flow cytometry (n = 6 wells for DMSO treated, n = 3 wells for all other conditions; *P* values calculated only for ITC-treated conditions by linear regression with adjustment for multiple testing by Holm’s method, shown only if significant)

To identify factors downstream of ITC that mediate the perturbations to endolysosomal function we observed above, we proceeded to perform targeted CRISPR-based inhibition (CRISPRi) screens against the “druggable genome” [37] in iAstrocytes treated with vehicle or ITC (see Methods, Additional file 8) using cell-surface LAMP1 or LysoTracker staining as proxies of endolysosomal system function (Fig. 3a). Consistent with our observations from the bafilomycin A1 titration curve, the phenotype scores (labeled as “gene score”, Methods, Additional file 8) of hits from the cell-surface LAMP1 screens were on average inversely correlated with those from the LysoTracker screens (Fig. 3b, d). Notably, *MTOR* was a top hit in both screens (Fig. 3d), and we also found a strong enrichment for genes associated with the mTOR pathway in the top hits from both screens (Fig. 3c, d). These results corroborate in hiPSC-derived astrocytes the rich literature on the regulation of autophagy and lysosome function by mTOR [38, 39], much of which was based on experiments in transformed human cell lines, yeast, or animal models.

To validate our screening results, we first assessed whether ITC increased the activity of mTORC1 or mTORC2, the two protein complexes formed by mTOR with distinct regulatory subunits and downstream signaling pathways [40]. We found that ITC increased mTORC1 activity as measured by increased levels of Ser235/236-phosphoryated S6 ribosomal protein (hereon referred to as phospho-S6), Thr37/46-phosphorylated 4E-BP1, and Ser757-phosphorylated ULK1 [38, 41] (Fig. 3e–i). ITC also increased mTORC2 activity as measured by Ser473-phosphorylated AKT1 [42] (Fig. 3j). To see if there was evidence of mTOR activation in inflammatory reactive astrocytes more broadly across different contexts, models, and species, we extracted differentially expressed genes in astrocytes from a variety of published rodent and human transcriptomic datasets and found broad enrichment for mTOR pathway-associated genes among upregulated genes (Supplementary Fig. 2), including in a recently published dataset demonstrating a role for astrocyte mTOR activation in experimental autoimmune encephalitis (EAE) [43], a mouse model of multiple sclerosis. Next, we tested the effect of mTOR inhibitors on cell-surface LAMP1 and LysoTracker staining. Consistent with the phenotype scores observed for *MTOR* knockdown in our CRISPRi screens, both rapamycin and Rapalink-1 [44] decreased cell-surface LAMP1 levels and increased LysoTracker staining (Fig. 3k, l), regardless of ITC treatment, although the effect of rapamycin on LysoTracker staining was not statistically significant. Given our factorial experimental design, we also analyzed our data with two-way ANOVA (see Methods, Additional file 8). Rapamycin and Rapalink-1 decreased both baseline cell-surface LAMP1 (statistically significant main effect terms) as well as reversing the ITC-induced increase in cell-surface LAMP1 (statistically significant interaction terms) (Table S4, Additional file 6, tab Fig. 3k). For LysoTracker staining, Rapalink-1 increased both baseline LysoTracker staining as well as exacerbating the ITC-induced decrease in LysoTracker staining, but the effects of rapamycin were not statistically significant (Table S4, Additional file 6, tab Fig. 3l). Both drugs significantly decreased phospho-S6 levels at baseline as well as abrogating the ITC-induced increase (Supplementary Fig. 3a; Table S4, Additional file 6, tab SupFig3a), demonstrating target engagement.

**Fig. 4:**
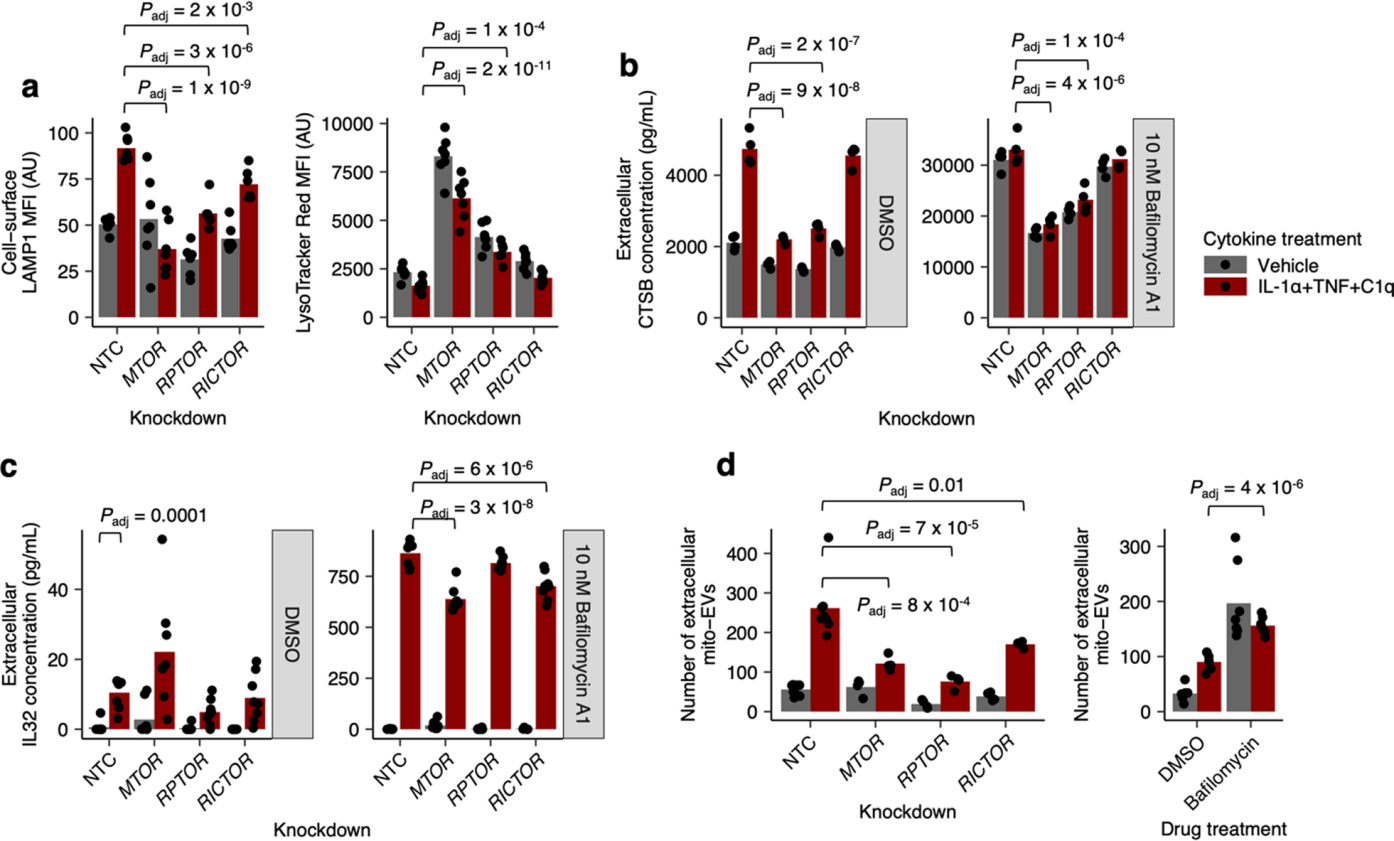
Cytokine-induced remodeling or pharmacological disruption of the endolyosomal system results in increased exocytosis of endolysosomal contents. **a**–**d** Cell-surface LAMP1 or LysoTracker median fluorescence intensity (MFI) measured by flow cytometry (**a**), extracellular CTSB concentration measured by electrochemiluminescence-based immunoassay (**b**), extracellular IL-32 concentration measured by ELISA (**c**), or abundance of extracellular mito-EVs measured by flow cytometry (**d**) in ITC- vs. vehicle-treated iAstrocytes transduced with non-targeting control (NTC) sgRNAs or sgRNAs targeting genes encoding common (*MTOR*) or unique mTORC1 (*RPTOR*) vs mTORC2 (*RICTOR*) subunits, with or without co-treatment with bafilomycin A1. *P* values were calculated by linear regression with correction for multiple testing using Holm’s method, shown only when significant

Focusing on mTOR as a central regulator of endolysosomal system function, we next explored how modulating mTORC1 or mTORC2 activity affected the exocytic activity of endolysosomal pathways. First, we tested whether the changes in cell-surface LAMP1 and LysoTracker staining caused by ITC depended on mTORC1 vs. mTORC2. We found that knockdown of *MTOR* decreased cell-surface LAMP1 and increased LysoTracker staining in ITC-treated astrocytes, which was phenocopied to a larger degree by knockdown of *RPTOR* (which encodes a subunit unique mTORC1) [45] than by knockdown of *RICTOR* (which encodes a subunit unique to mTORC2) [45] (Fig. 4a), consistent with known mTORC1-dependent mechanisms regulating autophagy and lysosome function [38, 39]. We verified that we achieved robust knockdown of mTOR by both directly measuring mTOR protein levels as well as downstream phospho-S6 levels (Supplementary Fig. 3b, c). A robust decrease in downstream phospho-S6 levels with *RPTOR* knockdown also verified Raptor protein depletion.

As we did previously with mTOR inhibitors, we also analyzed the mTORC1/2 subunit knockdown data by two-way ANOVA. Whereas the interaction between *MTOR* knockdown and ITC-treatment was statistically significant for both cell-surface LAMP1 and LysoTracker staining, the interactions between ITC treatment and *RPTOR* or *RICTOR* knockdown were not statistically significant for either cell-surface LAMP1 or LysoTracker staining (Table S4, Additional file 6, tabs Fig. 4a, b). This discrepancy suggests that knockdown of *RPTOR* or *RICTOR* may not cleanly inhibit mTORC1 or mTORC2 activity respectively without causing second-order changes. Indeed, we saw that *RICTOR* knockdown caused an increase in phospho-S6 levels and mTOR protein levels (Supplementary Fig. 3b, c), demonstrating that blocking mTORC2 activity can cause a compensatory increase in mTORC1 activity. Thus, phenotypes associated with *RPTOR* or *RICTOR* knockdown need to be interpreted with caution, as they likely do not reflect the effect of perturbing *only* mTORC1 or mTORC2 activity, respectively.

In addition to decreasing mTORC1 activity with *RPTOR* knockdown, we also tested the effect of increasing mTORC1 activity through knockdown of *TSC1*, an upstream inhibitor of mTORC1 [41]. We found that *TSC1* knockdown increased cell-surface LAMP1 and decreased LysoTracker staining (Supplementary Fig. 4a, b), as one would predict. However, similar to what we observed with *RPTOR* knockdown, the interaction term between *TSC1* knockdown and ITC-treatment was not statistically significant (Table S4, Additional file 6, tabs SupFig4a, b).

Next, we assessed how mTORC1 vs. mTORC2 controlled the exocytic activity of endolysosomal pathways by measuring the extracellular levels of three different endolysosomal cargos (see Methods, Additional file 8): cathepsin B (CTSB), a lysosome-enriched protease known to be released extracellularly by astrocytes [46]; IL-32, which we identified as being enriched in endolysosomal compartments from our proteomics dataset, consistent with prior observations [47]; and mitochondria-containing extracellular vesicles (mito-EVs) (see Methods, Additional file 8 and Supplementary Fig. 5), which are known to be released by astrocytes and are thought to be derived from mitophagy [48, 49]. We found that ITC increased extracellular levels of CTSB, IL-32, as well as mito-EVs, all of which were dramatically boosted by addition of bafilomycin A1 (Fig. 4b–d). Furthermore, *MTOR* knockdown decreased the exocytosis of CTSB, IL-32, and mito-EVs, although for IL-32 we only observed a statistically significant effect in the presence of bafilomycin A1 (Fig. 4b–d). *RPTOR* knockdown phenocopied *MTOR* knockdown to a larger degree than *RICTOR* knockdown for CTSB and mito-EVs, whereas for IL-32 *RICTOR* knockdown had a stronger effect than *RPTOR* knockdown (Fig. 4b–d). This trend was also evident by examining the interaction terms for *MTOR* vs *RICTOR* vs *RPTOR* knockdown with ITC treatment: for CTSB and mito-EVs, the interaction between ITC treatment and *MTOR* or *RPTOR* knockdown, but not *RICTOR* knockdown, was statistically significant (in the absence of bafilomycin A1); whereas for IL-32, the interaction ITC treatment and *RICTOR* knockdown, but not *MTOR* or *RPTOR* knockdown, was statistically significant (in the presence of bafilomycin A1) (Table S4, Additional file 6, tabs Fig. 4b–d). On the other hand, *TSC1* knockdown increased the exocytosis of mito-EVs and CTSB (Supplementary Fig. 4c, d), as expected, with statistically significant interaction terms as well (Table S4, Additional file 6, tabs SupFig4c-d). Overall, the stronger dependence on mTORC1 for the exocytosis of CTSB and mito-EVs compared to IL-32 suggests that IL-32 exocytosis may occur via a different endolysosomal pathway compared to CTSB and mito-EVs.

**Fig. 5:**
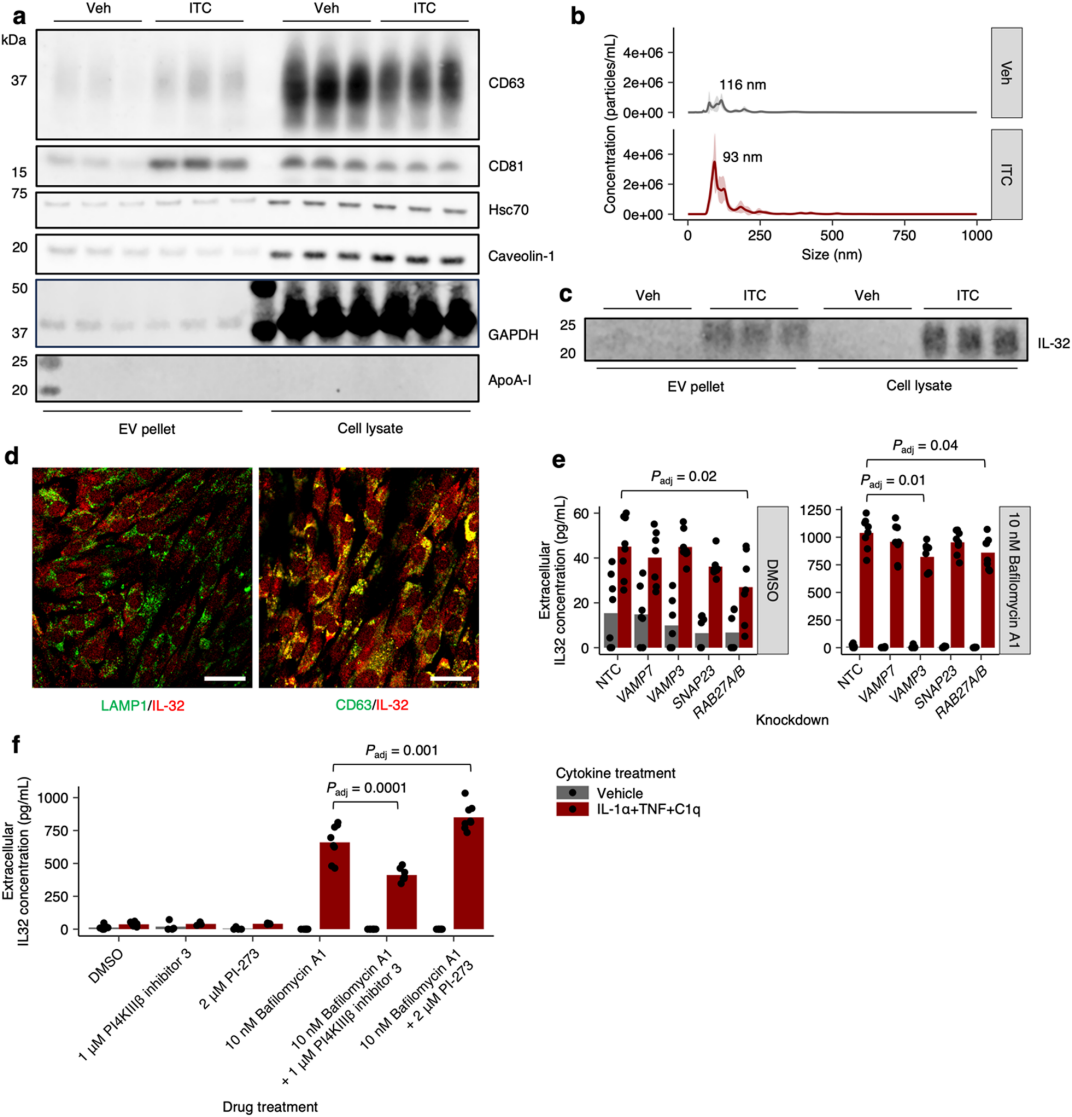
Extracellular IL-32 co-fractionates with extracellular vesicles likely derived from multivesicular body exocytosis. **a** Immunoblots against consensus extracellular vesicle (EV) markers (CD63, CD81, Hsc70, Caveolin-1) or common contaminants (e.g. ApoA-I) in EVs isolated from iAstrocyte conditioned media or total cell lysate after vehicle vs. ITC treatment. **b** EV size distribution measured by nanoparticle tracking analysis. **c** Immunoblot against IL-32 in EVs isolated from iAstrocyte conditioned media or total cell lysate. **d** Representative images of dual immunostaining against LAMP1 together with IL-32 or CD63 together with IL-32; scale bar = 60 μm. **e**, **f** Extracellular IL-32 concentration measured by ELISA in conditioned media from ITC- vs. vehicle-treated iAstrocytes transduced with non-targeting (NTC) sgRNAs or sgRNAs targeting genes encoding proteins involved in multivesicular body exocytosis (**e**), or treated with small molecules known to inhibit (PI4KIIIβ inhibitor 3) or not inhibit (PI-273) exosome biogenesis (**f**). *P* values were calculated by linear regression with correction for multiple testing by Holm’s method, shown only when significant

Given prior work which suggested that extracellular IL-32 is membrane-associated and possibly a component of extracellular vesicles [47, 50, 51], we proceeded to further characterize the secretion mechanism of IL-32 by isolating extracellular vesicles (EVs) from iAstrocyte conditioned media via differential ultracentrifugation (see Methods, Additional file 8), with or without ITC treatment. By western blotting, we verified that our EV preparation contained high levels of consensus EV markers such as CD63, CD81, Hsc70, and caveolin-1 [52] and was of acceptable purity, containing undetectable levels of apo-lipoproteins such as ApoA-I (Fig. 5a, Additional file 1), in accordance with the MISEV guidelines [52]. On nanoparticle tracking analysis (see Methods, Additional file 8), we saw that the size distribution of EVs from our preparations centered around a diameter of 100 nm, as expected [52] (Fig. 5b). Both western blotting and nanoparticle tracking analysis suggested an increase in the concentration of EVs with ITC treatment (Fig. 5a, b, Supplementary Fig. 6).

Having validated our EV preparation, we proceeded to blot for IL-32 and found that IL-32 was present in EVs isolated from ITC-treated but not vehicle-treated iAstrocytes (Fig. 5c). To see if extracellular IL-32 was exclusively EV-associated, we analyzed the EV pellet and supernatant by western blotting and ELISA. On western blot, IL-32 was detectable only in the EV pellet, as were EV markers (Supplementary Fig. 7a). However, by ELISA, we saw that extracellular IL-32 partitioned roughly equally between the EV pellet and supernatant (Supplementary Fig. 7b). In comparison, CTSB exclusively partitioned into the supernatant (Supplementary Fig. 7c). The discrepancy between the western blot and ELISA data for IL-32 could potentially be explained by the fact that the western blot and ELISA antibodies are polyclonal antibodies likely raised against different immunogens. Perhaps the ELISA antibodies detect an IL-32 isoform not detected by the western blot antibody.

Since EVs can be derived from direct budding of the plasma membrane or multivesicular body exocytosis (which would then be referred to as exosomes) [52], we performed immunostaining of ITC-treated iAstrocytes and visualized the subcellular localization of IL-32 with confocal microscopy. We found that IL-32 co-localized strongly with CD63 (Fig. 5d, Supplementary Fig. 8), which is also a marker of multivesicular bodies [53], suggesting that extracellular IL-32 is derived from multivesicular body exocytosis. We proceeded to further test this hypothesis by observing the effect of bafilomycin A1 or knockdown of genes encoding proteins involved in multivesicular body exocytosis on extracellular IL-32 levels. In agreement with prior reports which demonstrated increased exosome release with bafilomycin A1 [34, 36, 54], we found that treatment with bafilomycin A1 dramatically increased extracellular IL-32 levels (Fig. 5e). Furthermore, knockdown of *RAB27A/B* decreased extracellular IL-32 levels (Fig. 5e), consistent with the known role of RAB27A/B in exosome secretion [55]. Lastly, corroborating a prior report which identified a role for PI4KIIIβ in exosome biogenesis [54], we found that inhibition of PI4KIIIβ (using PI4KIIIβ inhibitor 3 [56]) but not PI4KIIα (using PI-273 [57]) decreased extracellular IL-32 levels in the presence of bafilomycin A1 (Fig. 5f).

**Fig. 6:**
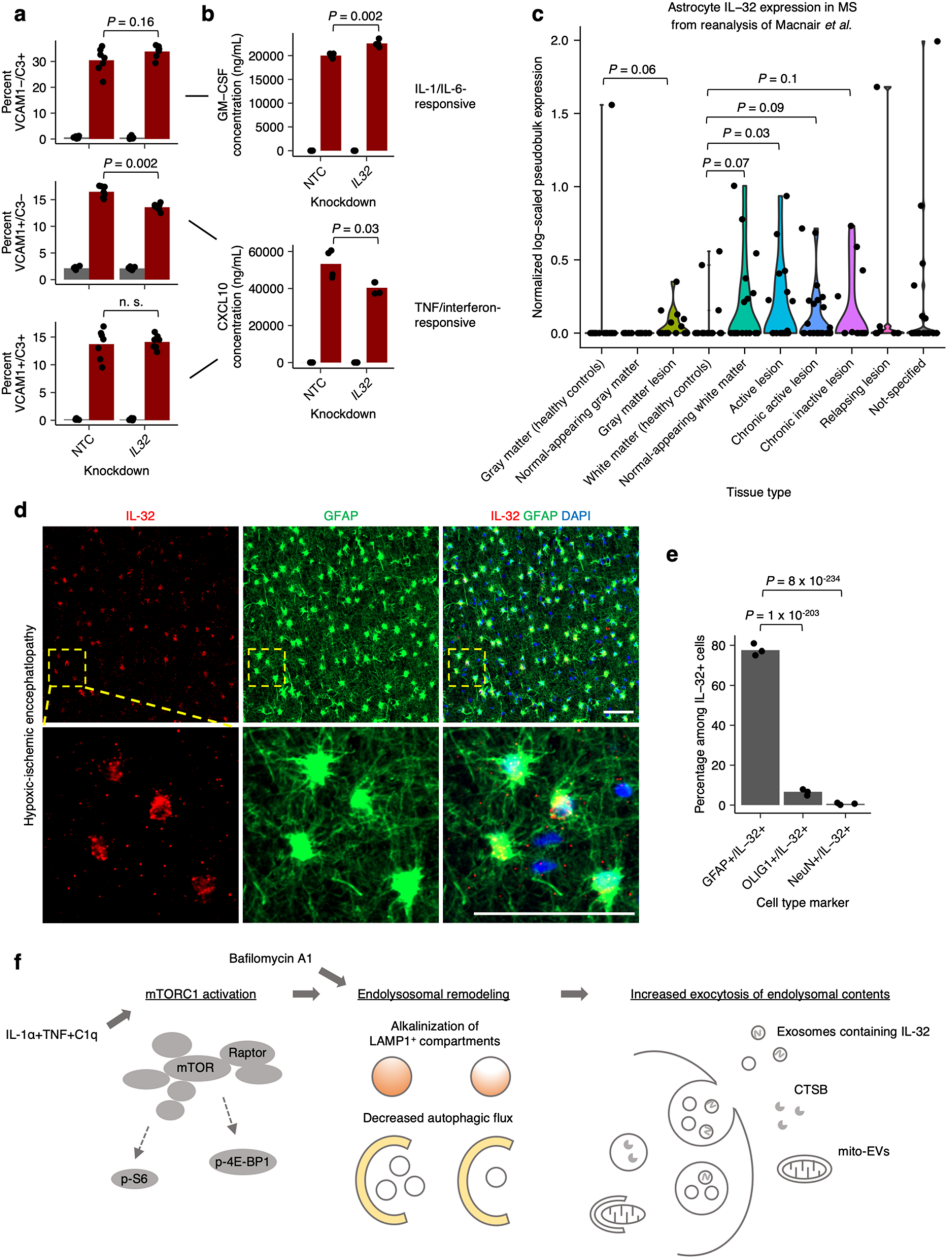
IL-32 regulates the polarization of inflammatory reactive astrocytes and is upregulated in astrocytes in neuroinflammatory conditions. **a**, **b** Proportion of IL-1/IL-6-responsive (VCAM1-/C3 +) or TNF/interferon-responsive (VCAM1 + /C3-, VCAM1 + /C3 +) inflammatory reactive astrocyte polarizations (**a**) or their associated cytokines (**b**) in ITC- vs. vehicle-treated iAstrocytes transduced with non-targeting (NTC) sgRNAs or sgRNAs targeting *IL32*. **c** Log-scaled *IL32* expression in astrocytes found in normal tissue vs. multiple-sclerosis lesions derived from pseudobulk analysis of snRNA-seq data from Macnair et al*.*; n = 15 for healthy control gray matter, n = 15 for normal-appearing gray matter, n = 15 for gray matter lesion, n = 22 for healthy control white matter, n = 18 for normal-appearing white matter, n = 17 for active lesion, n = 27 for chronic active lesion, n = 13 for chronic inactive lesion, n = 8 for relapsing lesion, n = 23 for not specified. **d** Representative immunostaining of IL-32 and GFAP in white matter brain tissue from patients with hypoxic-ischemic encephalopathy (HIE); scale bar 50 μm. **e** Percent GFAP + , OLIG1 + , or NeuN + cells among IL-32 + cells in HIE brain tissue (n = 3 patients); *P* values calculated via beta regression. **f**, Schematic of ITC-induced, mTOR-dependent endolysosomal remodeling and associated exocytic activity. *P* values were calculated using the Mann–Whitney U test in **a**, **c**, and **e**, and using the two-sided Student’s t test in **b**

Having explored the secretion mechanism of IL-32, we subsequently characterized its function in inflammatory astrocyte reactivity. Our prior work established two distinct polarizations of inflammatory reactive astrocytes after ITC treatment—a VCAM1^+^ interferon/TNF-responsive polarization associated with CXCL10 secretion vs. a VCAM1^−^/C3^+^ IL-1/IL-6-responsive polarization associated with GM-CSF secretion [9]. After verifying that knockdown of *IL32* resulted in robust depletion of IL-32 at the protein level (Supplementary Fig. 9a, b), we found that *IL32* knockdown subtly decreased the proportion of VCAM1^+^/C3^−^ astrocytes and noticeably decreased CXCL10 secretion (Fig. 6a, b), suggesting that IL-32 promotes the interferon/TNF-responsive polarization. Also, IL-32 induction by ITC was greater in VCAM1^+^/C3^+^ interferon-responsive astrocytes compared to IL-1/IL-6-rresponsive VCAM1^−^/C3^+^ astrocytes (Supplementary Fig. 9c–e). Furthermore, we found that IFN-β increased the upregulation of IL-32 by ITC (Supplementary Fig. 9f), consistent with prior work demonstrating a role of IL-32 in antiviral responses [58–60].

Given our finding that extracellular IL-32 is associated with EVs, we wondered if free extracellular IL-32 could influence inflammatory reactive astrocyte states. We found that treatment with recombinant IL-32β or IL-32γ at 200 ng/mL did not appreciably alter the proportion of VCAM1^+^/C3^−^ astrocytes, regardless of *IL32* knockdown (Supplementary Fig. 9f). As the concentration of IL-32β and IL-32γ we used here is well above the range where immune cells could be robustly activated by recombinant IL-32 [61], our data suggests that the effect of IL-32 on inflammatory reactive astrocytes states may be mediated intracellularly, either through uptake of IL-32-containing EVs or cell-autonomously through IL-32 that has not been secreted.

Next, given that IL-32 levels have been reported to be elevated in the cerebrospinal fluid of patients with multiple sclerosis and neuro-Behcet’s disease [15], we wanted to see if IL-32 was upregulated in astrocytes under neuroinflammatory conditions. Although the fact that IL-32 does not have an ortholog in rodents precluded us from analyzing the vast amount of published transcriptomic data on mouse models of neuroinflammation, we found that *IL32* transcript levels were indeed upregulated in astrocytes in various types of multiple sclerosis lesions in humans (Fig. 6c) by reanalyzing the comprehensive single-nucleus RNA-seq dataset from Macnair et al*.* [62] (121 subjects total) at the pseudobulk level [63] (see Methods, Additional file 8). There was also evidence of *IL32* upregulation in oligodendrocytes, microglia, and endothelial cells, but only in white matter lesions (Supplementary Fig. 10). Interestingly, astrocytes were the only cell type with evidence of *IL32* upregulation in gray matter lesions (Fig. 6c). To examine whether IL-32 could be found in other neuroinflammatory conditions, we stained for IL-32 and cell type markers in post-mortem brain tissue from pediatric patients diagnosed with hypoxic-ischemic encephalopathy, a condition involving significant neuroinflammation [63] where we had previously identified upregulation of inflammatory reactive astrocyte markers [9]. We found that IL-32 preferentially colocalized with GFAP^+^ astrocytes and to a much lesser degree with OLIG1 + oligodendrocytes (Fig. 6d, e). IL-32 was not detected in NeuN + neurons (Fig. 6e), consistent with our observations from the multiple sclerosis snRNA-seq data.

Finally, to ensure the overall robustness of our results, we validated key findings in iAstrocytes derived from an independent hiPSC line of different sex (162D). We confirmed that after ITC treatment, 162D iAstrocytes upregulated cell-surface LAMP1 (Supplementary Fig. 11a), accumulated LC3 and p62 puncta (Supplementary Fig. 11b, c), upregulated mTORC1 activity as measured by phospho-S6 staining (Supplementary Fig. 11d), upregulated IL-32 (Supplementary Fig. 11e), and secreted a greater number of mito-EVs (Supplementary Fig. 11 h). Furthermore, we also directly characterized lysosome function by assaying intracellular CTSB activity in both 162D iAstrocytes and WTC11 iAstrocytes; ITC treatment decreased intracellular CTSB activity in both 162D and WTC11 iAstrocytes (Supplementary Fig. 11 g).

## Discussion

Our results establish mTOR activation as a key feature of inflammatory astrocyte reactivity induced by IL-1α + TNF + C1q (ITC), driving endolysosomal remodeling manifesting as alkalinization of LAMP1^+^ compartments and reduced autophagic flux (Fig. 6e). While we have focused on mTOR-dependent endolysosomal remodeling driven by acute ITC treatment, other cellular processes may also contribute to endolysosomal remodeling. For example, cellular senescence is associated with a profound remodeling of lysosome function and content [64], which in fact may partially occur through mTORC1 hyperactivation [65]. Although acute treatment with inflammatory cytokines is unlikely to induce cellular senescence, we nevertheless found overlap of senescence-associated genesets with genes upregulated by ITC (Supplementary Fig. 2b). We speculate that perhaps chronic inflammatory activation of astrocytes could lead to cellular senescence which would further contribute to endolysosomal remodeling.

Connecting our results here with the broader literature on phenotypes associated with inflammatory astrocyte reactivity, we suspect that mTOR-induced endolysosomal remodeling may account for the loss of phagocytic activity observed in inflammatory reactive astrocytes [14]. Although we did not measure phagocytic activity here, we observed in our previously published CRISPRi screens on inflammatory reactivity that *MTOR* knockdown rescued the phagocytic deficit induced by ITC [9].

Here, we found that mTOR-dependent endolysosomal remodeling also resulted in the increased exocytosis of certain endolysosomal cargos (Fig. 6e), with increased cell-surface LAMP1 likely acting as a non-specific marker of endolysosomal exocytic activity. Interestingly, LAMP1^+^ astrocytes have been shown to modify disease progression in EAE [66]. Furthermore, all three of the endolysosomal cargos we have characterized—CTSB, IL-32, and mito-EVs—have been reported to be involved in neuroinflammatory conditions. CTSB levels are elevated in the CSF as well as brain parenchyma of patients with Alzheimer’s disease [67–70], and knockout of *Ctsb* has been shown to ameliorate the neuropathology and behavioral deficits in mouse models of Alzheimer’s disease [71–73]. Mito-EVs [74] have been shown to mediate the transfer of mitochondria from astrocytes to neurons after experimentally induced stroke in mice [48]. Lastly, IL-32 levels are elevated in the cerebrospinal fluid of patients with multiple sclerosis or neuro-Behcet’s disease [15], and a polymorphism in the IL-32 promoter has been associated with increased risk of multiple sclerosis in two independent studies [75, 76].

Given that IL-32 does not have an ortholog in rodents and that its secretion mechanism is still incompletely characterized, we focused on elucidating IL-32 secretion, taking advantage of our in vitro hiPSC-derived astrocyte platform. We found that after ITC treatment, intracellular IL-32 colocalized with multivesicular bodies, and that extracellular IL-32 co-fractionated with EVs. Whether extracellular IL-32 is exclusively associated with EVs remains to be seen, as we obtained conflicting data by western blotting vs. ELISA. Given that the western blot and ELISA antibodies are polyclonal antibodies likely raised against different immunogens, it is possible that the ELISA antibodies recognize a non-EV-associated IL-32 isoform not detected by the western blot antibody. Alternatively, if extracellular IL-32 consists predominantly of one isoform, it may be cleaved in a way such that the western blot antibody recognizes only the EV-associated fragment whereas the ELISA antibodies recognize both the EV-associated and free fragments. Further biochemical experiments will be necessary to elucidate whether IL-32 isoforms may be differentially associated with EVs or if extracellular cleavage of IL-32 occurs.

With respect to the secretion mechanism of extracellular IL-32, we found that knockdown of genes encoding proteins involved in multivesicular body exocytosis such as RAB27A/B decreased extracellular levels of IL-32, as did pharmacological inhibition of exosome biogenesis. Overall, our results corroborate previous reports demonstrating that a portion of extracellular IL-32 is vesicle-associated [50, 51], and we establish exosomes as the likely candidate. With respect to extracellular IL-32 not contained within EVs, it is possible that IL-32 may be loaded directly into multivesicular body lumens and subsequently exocytosed, or that intracellular IL-32 may be released directly through plasma membrane leakage in dying or dead cells [77].

As for the role of IL-32 in neuroinflammation, we found that knockdown of *IL32* affected the polarization of inflammatory reactive states induced by ITC [9], decreasing the abundance of the interferon/TNF-responsive state and its associated cytokine CXCL10. A limitation of knocking down *IL32* with DNA-targeting CRISPRi [78] is that we could not distinguish the contribution of the many splice isoforms of IL-32, which have been reported to have distinct activities [79]; future work could elucidate this using RNA interference or RNA-targeting CRISPR-based systems [80].

Regarding how IL-32 acts upon cells, it is an open question whether extracellular IL-32 signals through cell-surface receptors or exerts its effects intracellularly, for example, after EV-mediated uptake or cell-autonomously when it is not secreted. Depending on the cell type and biological context, there is evidence for both extracellular and intracellular activity [16]. In our hands, free extracellular recombinant IL-32 at concentrations capable of activating immune cells [61] did not appreciably influence inflammatory reactive astrocyte states, suggesting that our IL-32-associated phenotypes may be mediated intracellularly.

In addition to its effects on the polarization of inflammatory reactive astrocyte states, we also found that IL-32 was induced by IFN-β (an old disease-modifying treatment for multiple sclerosis), and that *IL32* transcript levels were upregulated in astrocytes in various multiple sclerosis lesions. Considering the human genetics data demonstrating the importance of IL-32 to the pathogenesis of multiple sclerosis, studying how astrocyte IL-32 contributes to multiple sclerosis would be a worthwhile future research direction. Lastly, we found preferential colocalization of IL-32 with astrocytes in a different neuroinflammatory condition—hypoxic-ischemic encephalopathy (HIE), suggesting that IL-32 may play a role in HIE as well.

In conclusion, we believe that our results highlight mTOR-dependent endolysosomal remodeling as an important and previously underappreciated aspect of inflammatory astrocyte reactivity which can be targeted therapeutically. We also clarified the secretion mechanism and functional role of an important disease-associated cytokine, IL-32, in astrocytes, a cell type in which IL-32 has rarely been studied [81]. Since the receptor for IL-32 is still unknown [16], we believe that our results establish a strong foundation for future studies focused on the how IL-32 mediates its biological effects and contributes to neuroinflammation.

### Supplementary Information

Additional file 1. Raw western blot images and associated metadata.

Additional file 2. TIRF microscopy movie of LysoTracker Green-loaded iAstrocytes expressing LAMP1-mCherry.

Additional file 3. Table S1: Gene-level log-scaled fold-change and p-value information derived from RNA-seq of multiple hiPSC-derived astrocyte models treated with ITC or similar treatments.

Additional file 4. Table S2: Endolysosomal-IP and total cell lysate proteomics data.

Additional file 5. Table S3: Metadata for studies included in gene set overlap analyses shown in Supplementary Figures 1-2.

Additional file 6. Table S4: Two-way ANOVA analysis results for experiments with factorial design.

Additional file 7: Supplementary Figures.

Additional file 8: Methods.

## Data Availability

The source data and code used to analyze the data presented in this study will be shared upon request. The source data for all western blots shown in this study can be found in Additional file 1.

## Abbreviations

CRISPR: Clustered regularly interspaced short palindromic repeats
CRISPRi: CRISPR-based gene expression inhibition
hiPSC: Human induced pluripotent stem cell
ITC: IL-1α + TNF + C1q
ELISA: Enzyme-linked immunosorbent assay
EV: Extracellular vesicle
EAE: Experimental autoimmune encephalitis
