# Supplementary material for: mTOR activation induces endolysosomal remodeling and nonclassical secretion of IL-32 via exosomes in inflammatory reactive astrocytes
Source: J Neuroinflammation. 2024 Aug 8;21:198. doi: 10.1186/s12974-024-03165-w (PMC11312292; doi:10.1186/s12974-024-03165-w)
Supplement: Supplementary file 1 — Additional file 1. Raw western blot images and associated metadata. [file 12974_2024_3165_MOESM1_ESM.zip › SupplementaryFile1_rawWBimages/Fig3/pS6_S6/BRExp225g_metadata.docx]

**BRExp225g:**

Samples 1-6: Veh

Samples 7-12: ITC

Blot 1:

Lanes 2-13: samples 1-12

Blot 2:

Lanes 3-14: samples 1-12

Round 1

Blot 1, top (>37 kDa):

Rb anti p-ULK1 (CST 6888), 1:1000 -> Gt anti Rb IRDye680 1:10,000

Ms anti LAMP1 (Abcam ab25630), 1:1000 -> Gt anti Ms IRDye800 1:10,000

Blot 1, bot (<37 kDa):

Rb anti p-S6 (CST 4858), 1:2000 -> Gt anti Rb IRDye680 1:10,000

Ms anti GAPDH (sc-47724), 1:500 -> Gt anti Ms IRDye800 1:10,000

Blot 2, top (>37 kDa):

Rb anti p-Akt (CST 9271), 1:1000 -> Gt anti Rb IRDye680 1:10,000

Ms anti pan-Akt (CST 2920), 1:2000 -> Gt anti Ms IRDye800 1:10,000

Blot 2 bot (<37 kDa):

None

Round 2

Blot 1, top (>37 kDa):

Rb anti p-ULK1 (CST 14202), 1:1000 -> Gt anti Rb IRDye680 1:10,000

Blot 1, bot (<37 kDa):

Rb anti S6 (CST 2217), 1:1000 -> Gt anti Rb IRDye680 1:10,000

Blot 2, top (>37 kDa):

Rb anti p-Akt (9271), 1:1000 -> Gt anti Rb IRDye680 1:10,000

Blot 2 bot (<37 kDa):

Ms anti GAPDH (sc-47724), 1:500 Gt anti Ms IRDye800 1:10,000
