# Supplementary figures and images for: mTOR activation induces endolysosomal remodeling and nonclassical secretion of IL-32 via exosomes in inflammatory reactive astrocytes
Source: J Neuroinflammation. 2024 Aug 8;21:198. doi: 10.1186/s12974-024-03165-w (PMC11312292; doi:10.1186/s12974-024-03165-w)

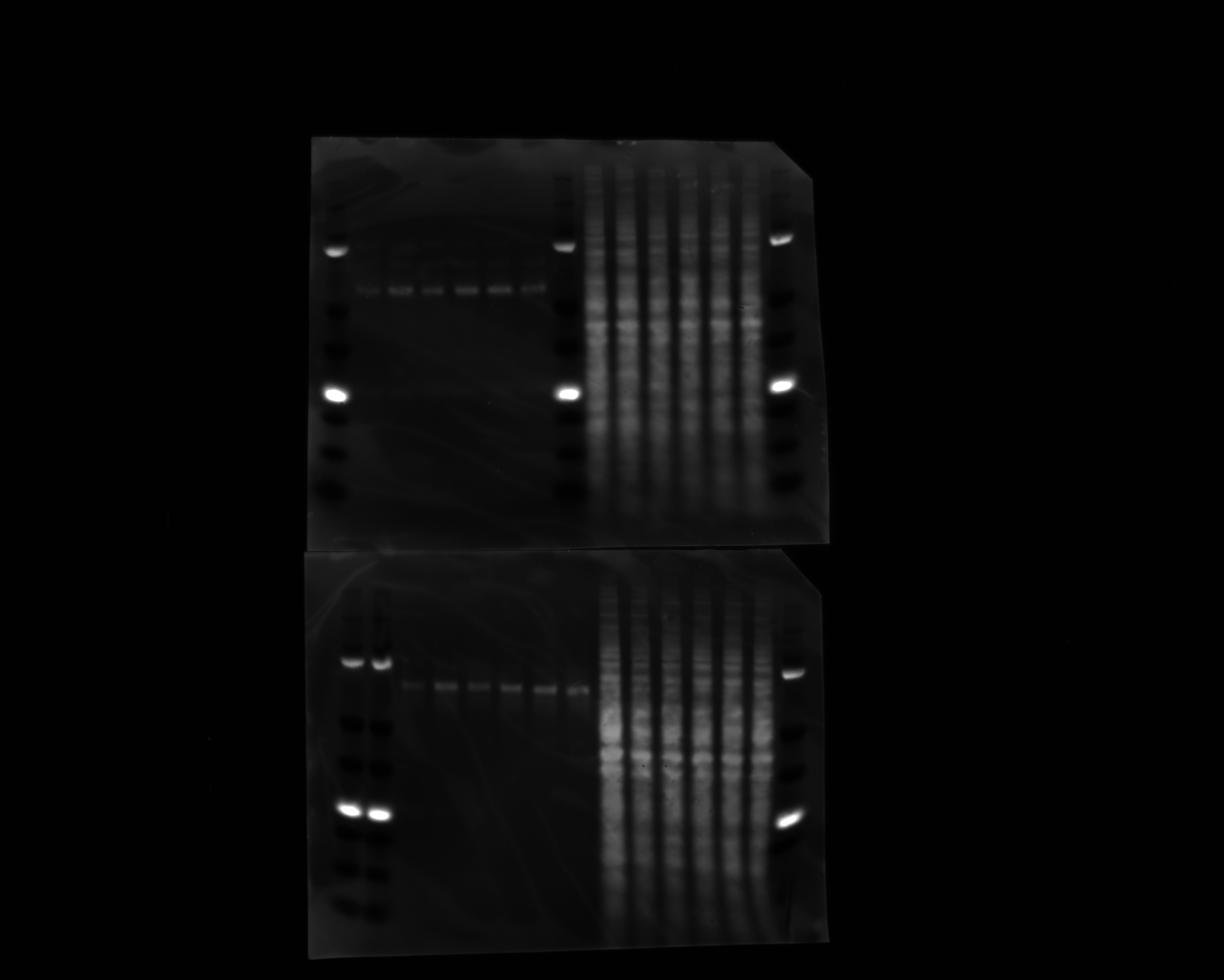

Supplement: Supplementary file 1 — Additional file 1. Raw western blot images and associated metadata. [file 12974_2024_3165_MOESM1_ESM.zip › SupplementaryFile1_rawWBimages/Fig5/Exp247b_set2_SyproRuby.raw16.tif]

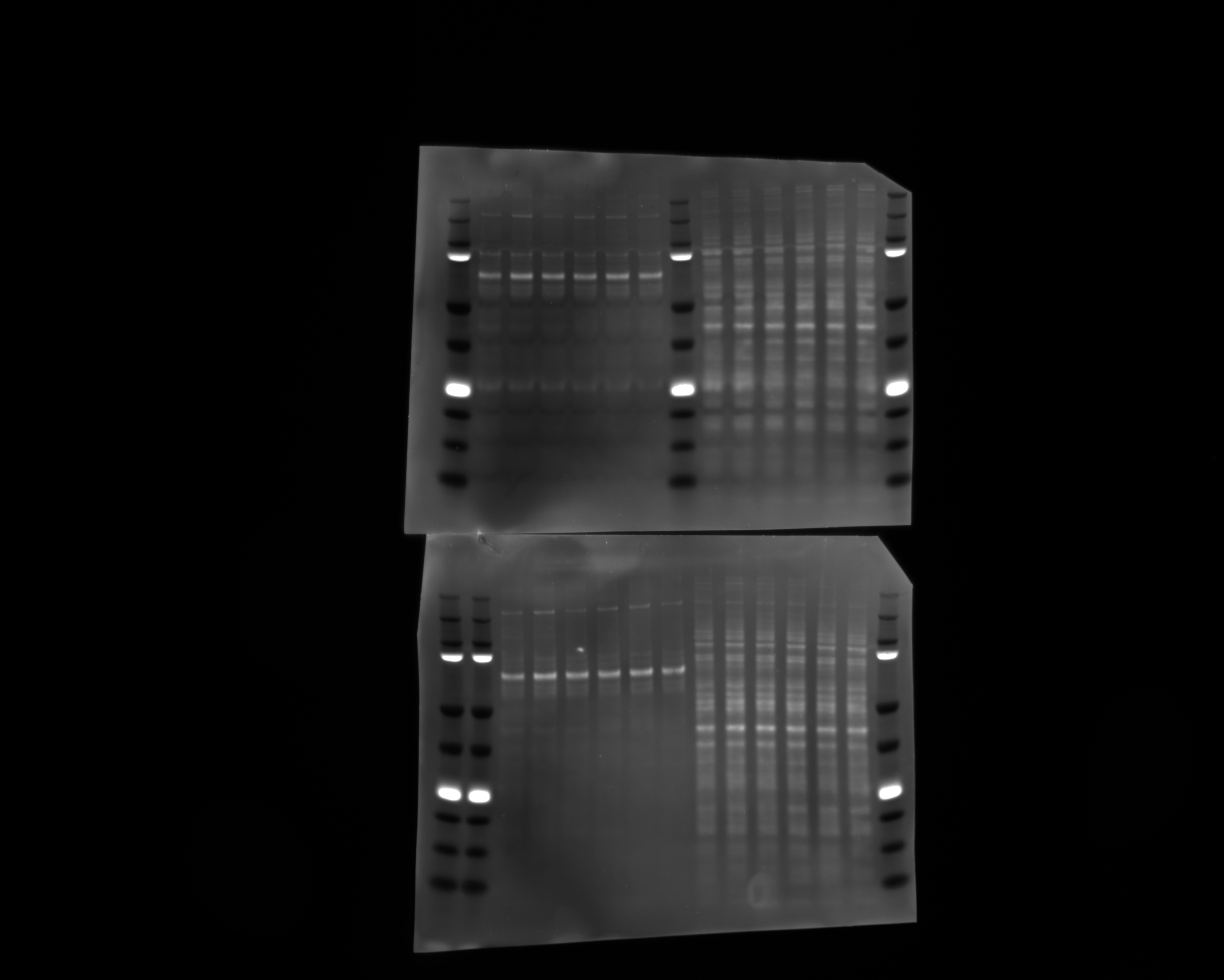

Supplement: Supplementary file 1 — Additional file 1. Raw western blot images and associated metadata. [file 12974_2024_3165_MOESM1_ESM.zip › SupplementaryFile1_rawWBimages/Fig5/Exp247c_set1_SyproRuby.raw16.tif]

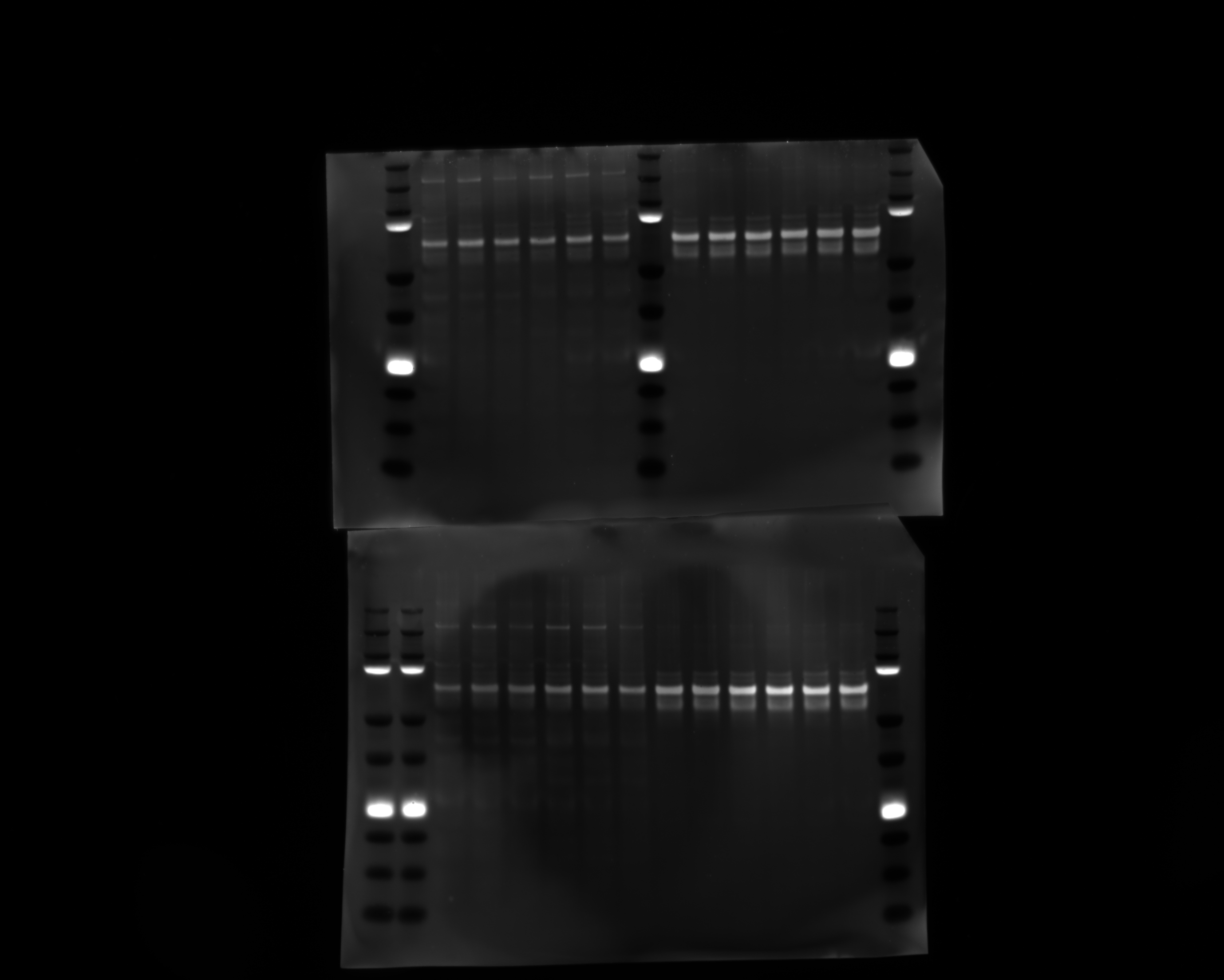

Supplement: Supplementary file 1 — Additional file 1. Raw western blot images and associated metadata. [file 12974_2024_3165_MOESM1_ESM.zip › SupplementaryFile1_rawWBimages/SuppFig7/Exp247c_set2_SyproRuby_ChemiDoc Images 2024-03-19_15.20.03/syproRUBY.raw16.tif]

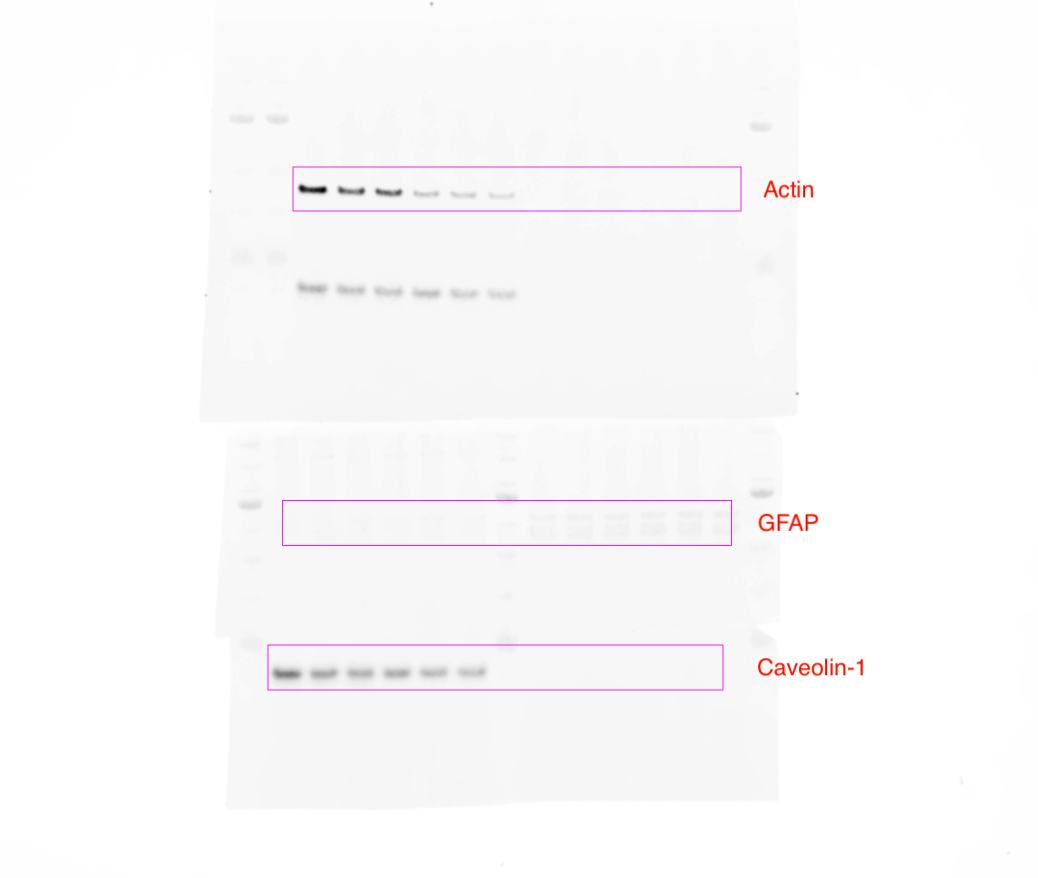

Supplement: Supplementary file 1 — Additional file 1. Raw western blot images and associated metadata. [file 12974_2024_3165_MOESM1_ESM.zip › SupplementaryFile1_rawWBimages/SuppFig7/KL032024_exp247c_set2_blots1and2_round1_0000615_01/0000615_01_800_croppedBands_Actin_GFAP_Cav1.png]

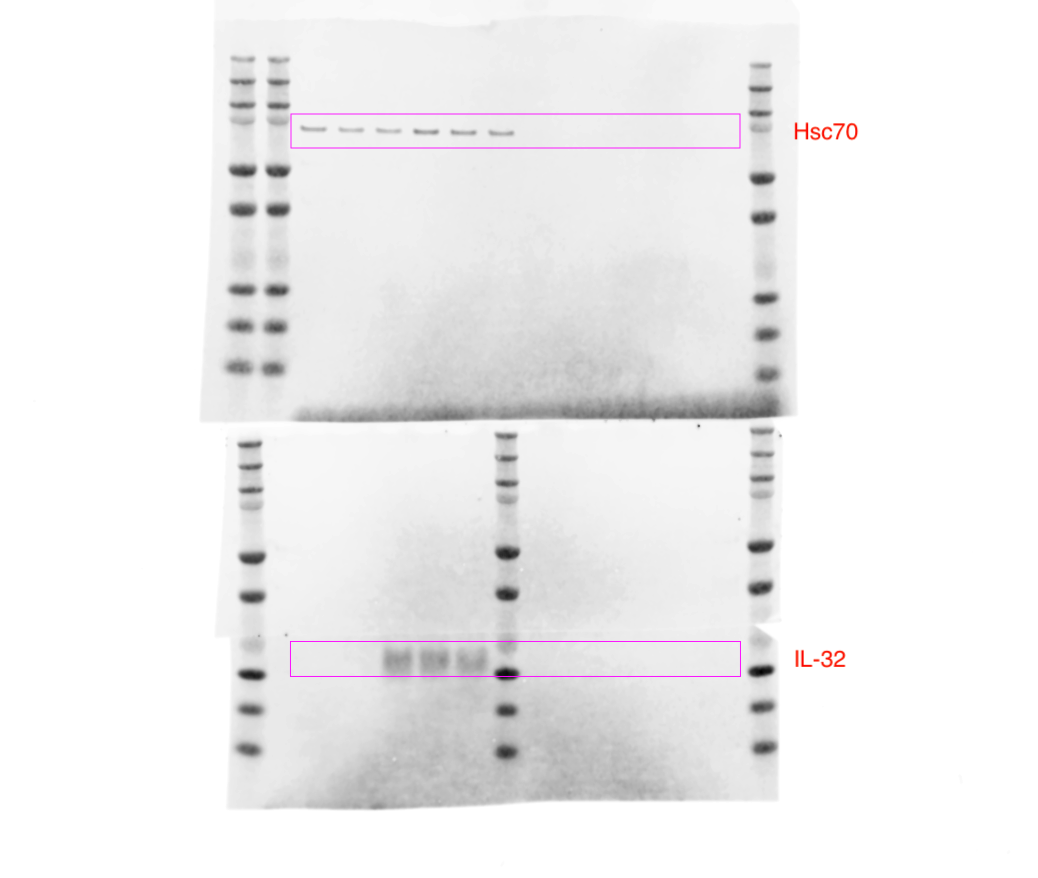

Supplement: Supplementary file 1 — Additional file 1. Raw western blot images and associated metadata. [file 12974_2024_3165_MOESM1_ESM.zip › SupplementaryFile1_rawWBimages/SuppFig7/KL032024_exp247c_set2_blots1and2_round1_0000615_01/0000615_01_700_croppedBands_Hsc70_IL32.png]

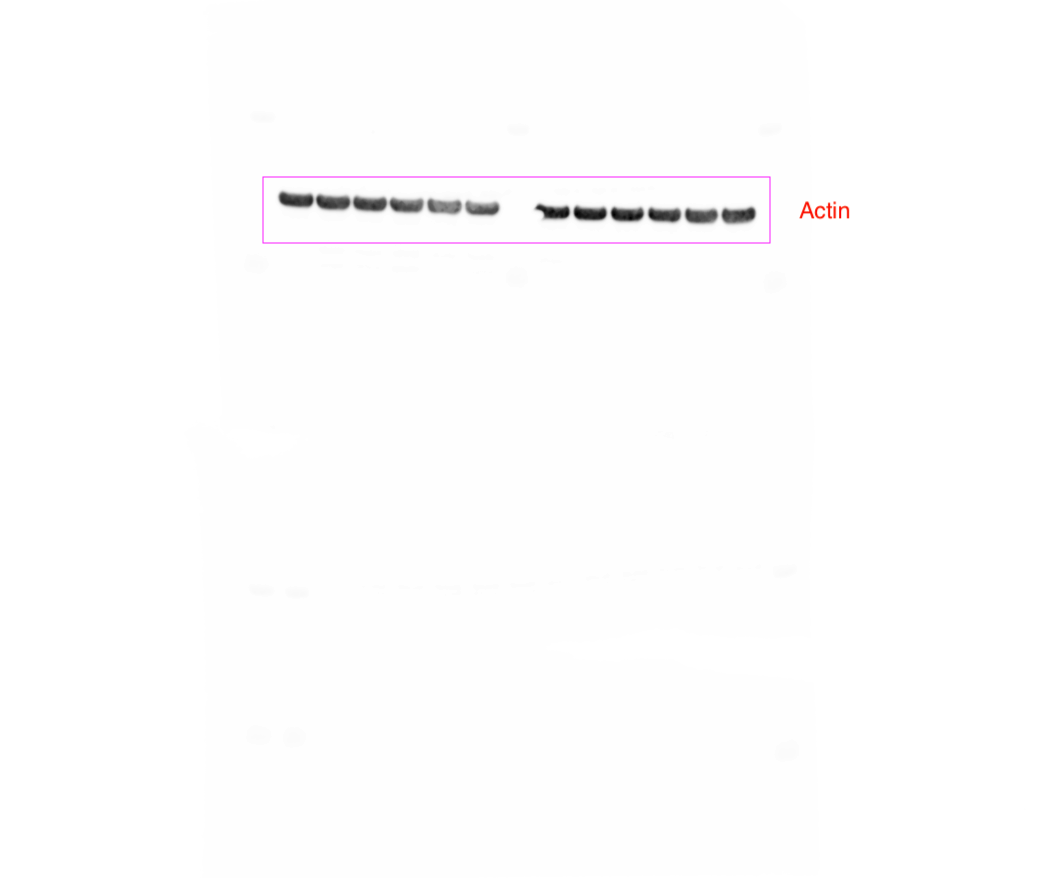

Supplement: Supplementary file 1 — Additional file 1. Raw western blot images and associated metadata. [file 12974_2024_3165_MOESM1_ESM.zip › SupplementaryFile1_rawWBimages/SuppFig9/KL032224_exp250a_WB_cellLysates_set1_blots1and2_round1_0000625_01/0000625_01_800_croppedBands_actin.png]

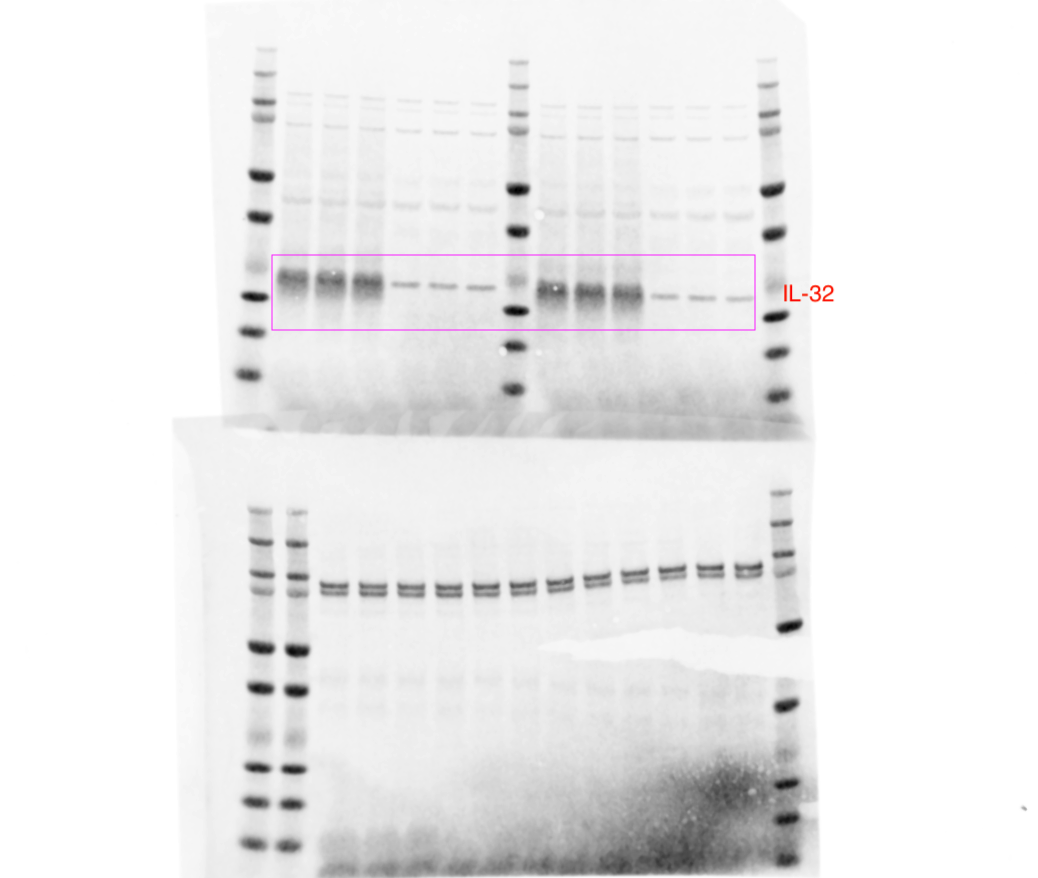

Supplement: Supplementary file 1 — Additional file 1. Raw western blot images and associated metadata. [file 12974_2024_3165_MOESM1_ESM.zip › SupplementaryFile1_rawWBimages/SuppFig9/KL032224_exp250a_WB_cellLysates_set1_blots1and2_round1_0000625_01/0000625_01_700_croppedBands_IL32.png]

Probe: anti-LAMP2 (top), anti-GAPDH (bottom)

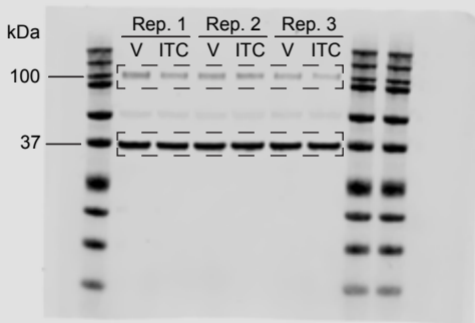

Supplement: Supplementary file 1 — Additional file 1. Raw western blot images and associated metadata. [file 12974_2024_3165_MOESM1_ESM.zip › SupplementaryFile1_rawWBimages/Fig1/LAMP1_LAMP2/LAMP2.pdf]

Probe: anti-LAMP1 (top), anti-MITF (bottom)

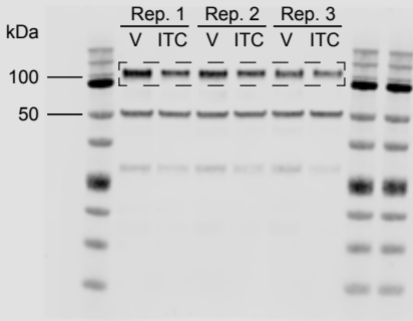

Supplement: Supplementary file 1 — Additional file 1. Raw western blot images and associated metadata. [file 12974_2024_3165_MOESM1_ESM.zip › SupplementaryFile1_rawWBimages/Fig1/LAMP1_LAMP2/LAMP1.pdf]

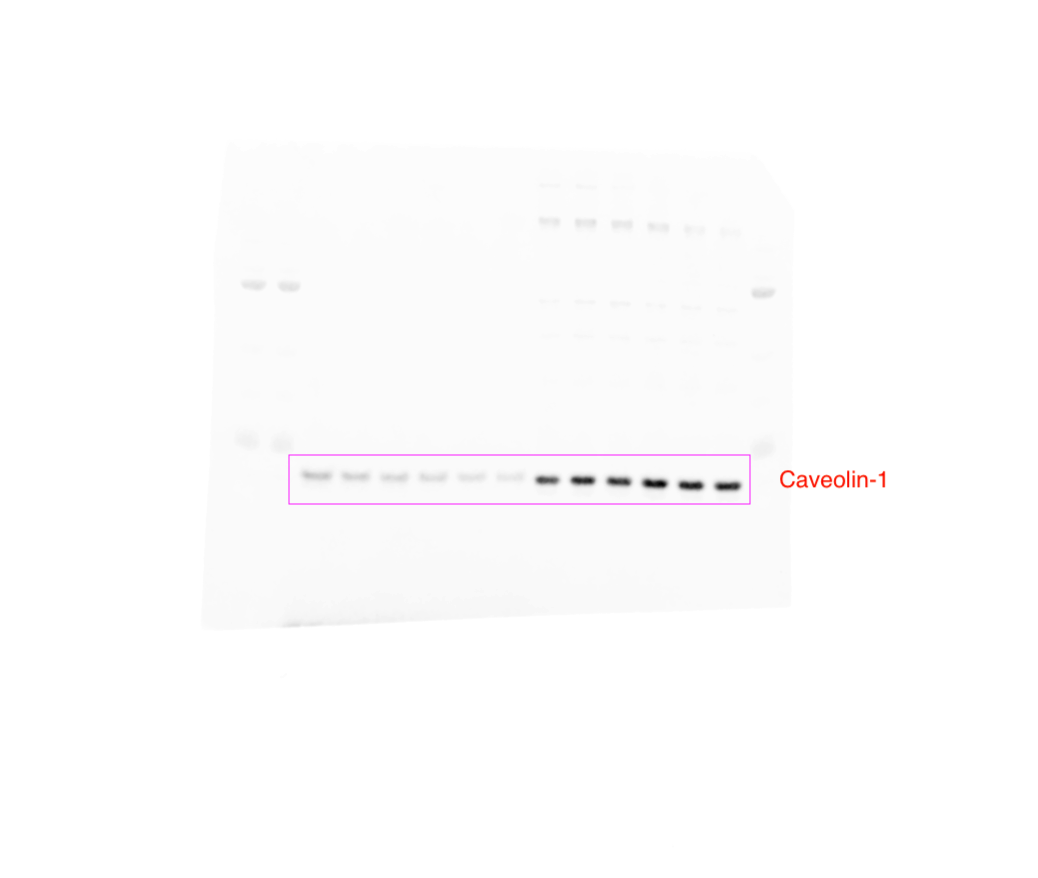

Supplement: Supplementary file 1 — Additional file 1. Raw western blot images and associated metadata. [file 12974_2024_3165_MOESM1_ESM.zip › SupplementaryFile1_rawWBimages/Fig5/KL030624_exp247c_set1_blot2_round1_0000546_01/0000546_01_800_croppedBands_Caveolin1.png]

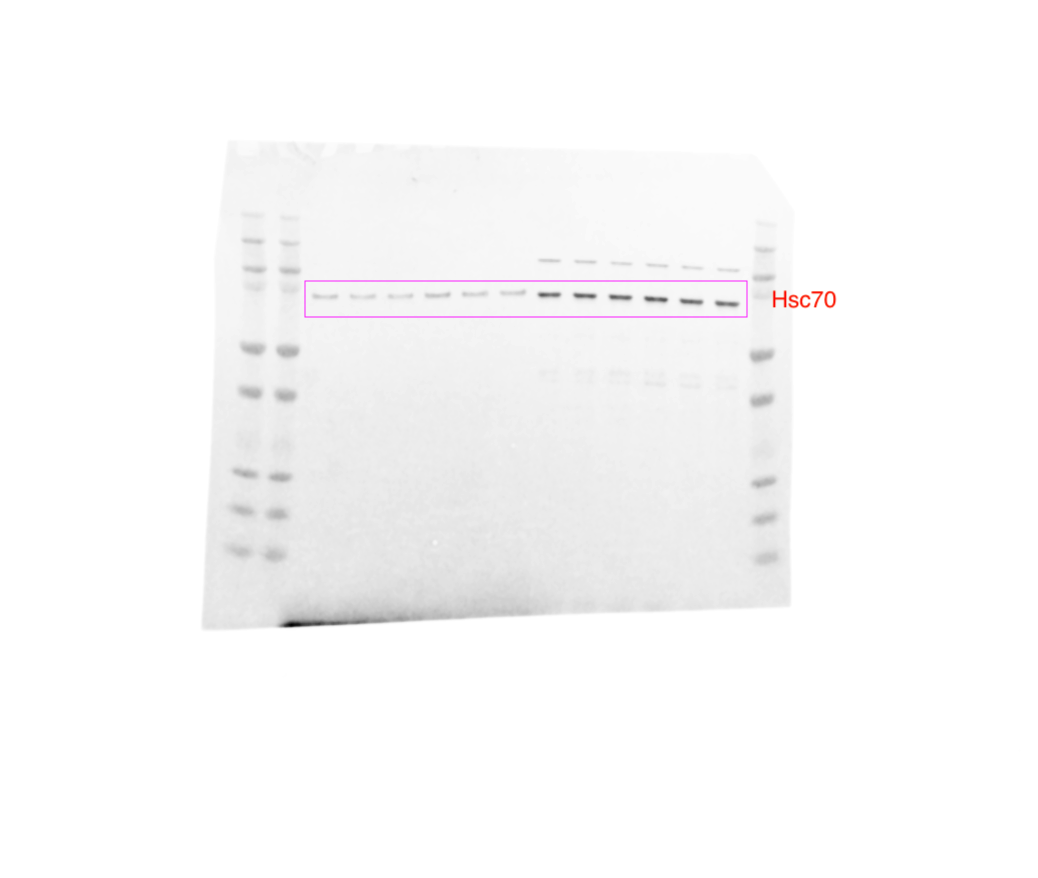

Supplement: Supplementary file 1 — Additional file 1. Raw western blot images and associated metadata. [file 12974_2024_3165_MOESM1_ESM.zip › SupplementaryFile1_rawWBimages/Fig5/KL030624_exp247c_set1_blot2_round1_0000546_01/0000546_01_700_croppedBands_Hsc70.png]

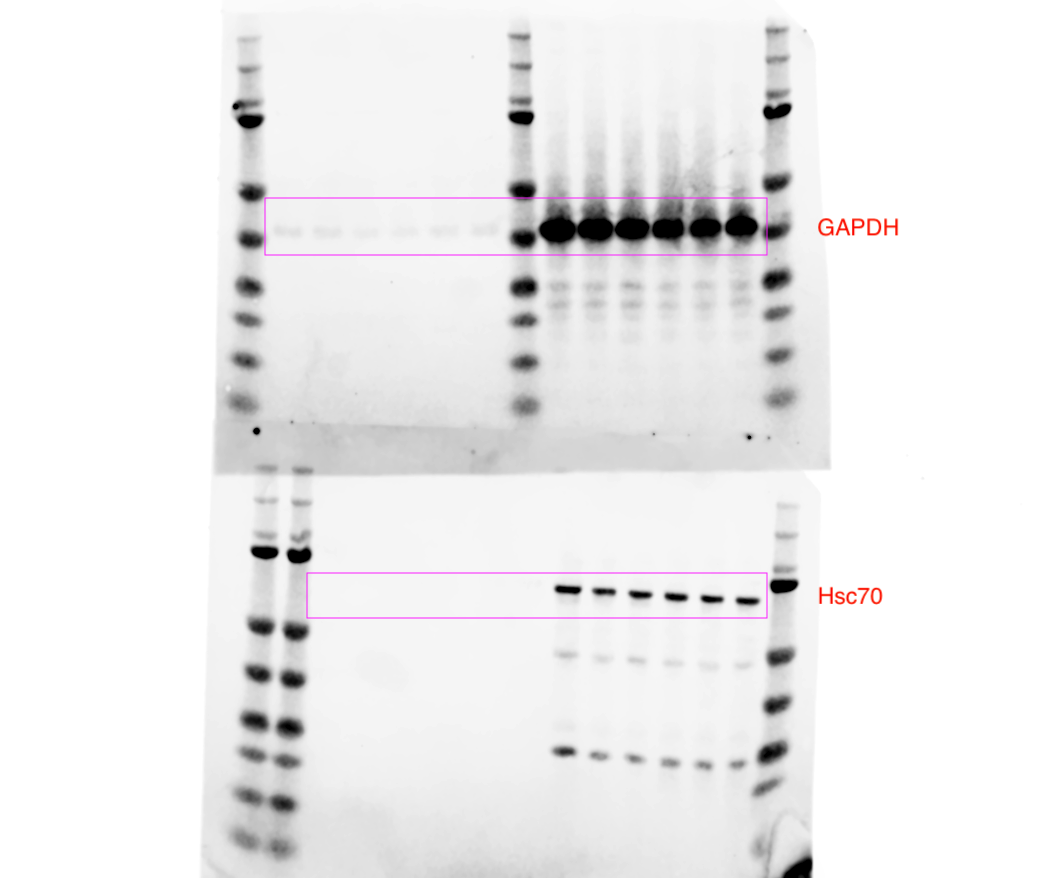

Supplement: Supplementary file 1 — Additional file 1. Raw western blot images and associated metadata. [file 12974_2024_3165_MOESM1_ESM.zip › SupplementaryFile1_rawWBimages/Fig5/KL011724_Exp247b_set2_blots1and2_round2_0000414_01/0000414_01_700_croppedBands_GAPDH.png]

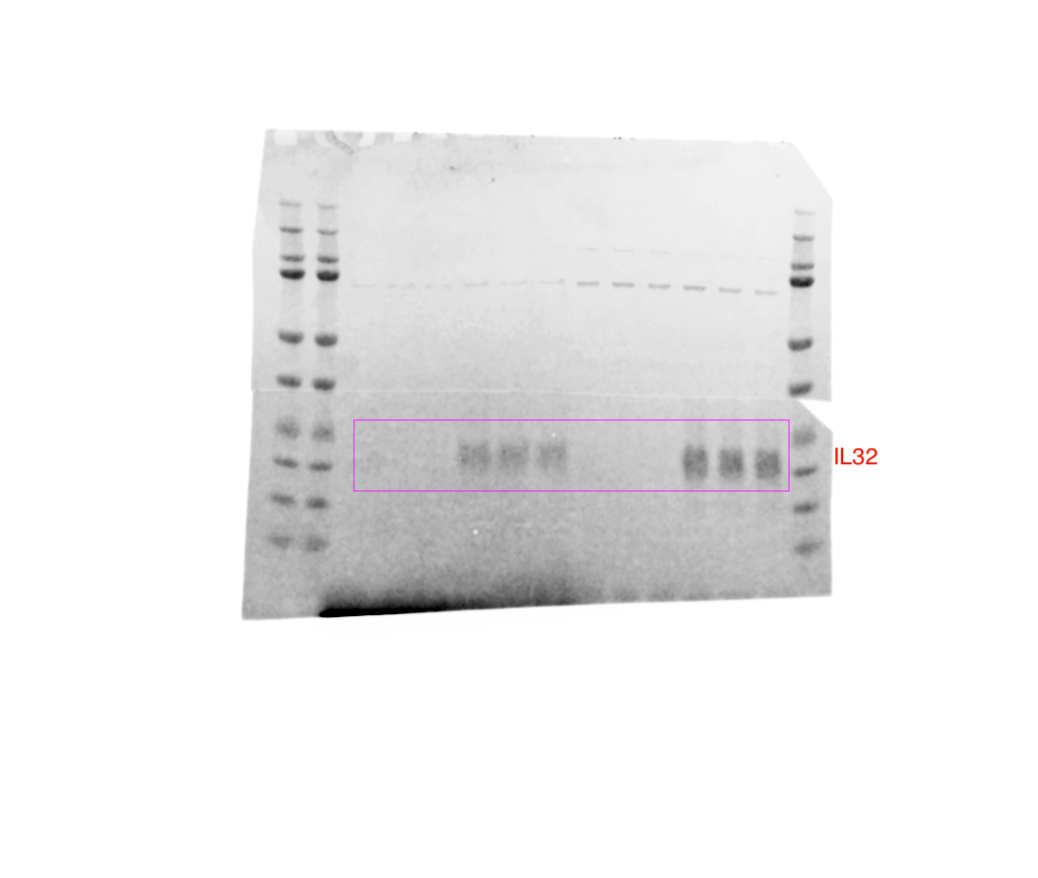

Supplement: Supplementary file 1 — Additional file 1. Raw western blot images and associated metadata. [file 12974_2024_3165_MOESM1_ESM.zip › SupplementaryFile1_rawWBimages/Fig5/KL030724_exp247c_set1_blot2_round2_0000548_01/0000548_01_700_croppedBands_IL32.png]

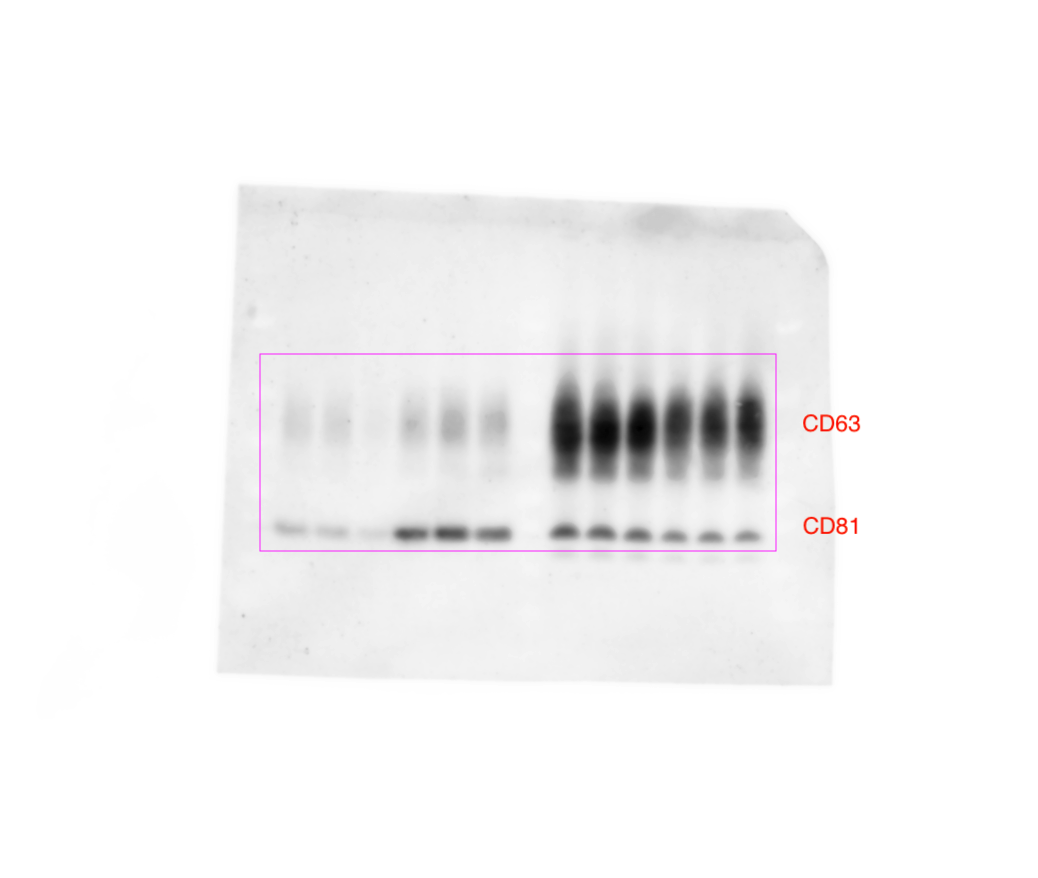

Supplement: Supplementary file 1 — Additional file 1. Raw western blot images and associated metadata. [file 12974_2024_3165_MOESM1_ESM.zip › SupplementaryFile1_rawWBimages/Fig5/KL011624_Exp247b_set2_blot1_round1_0000409_01/0000409_01_Chemi_croppedBands_CD63_CD81.png]

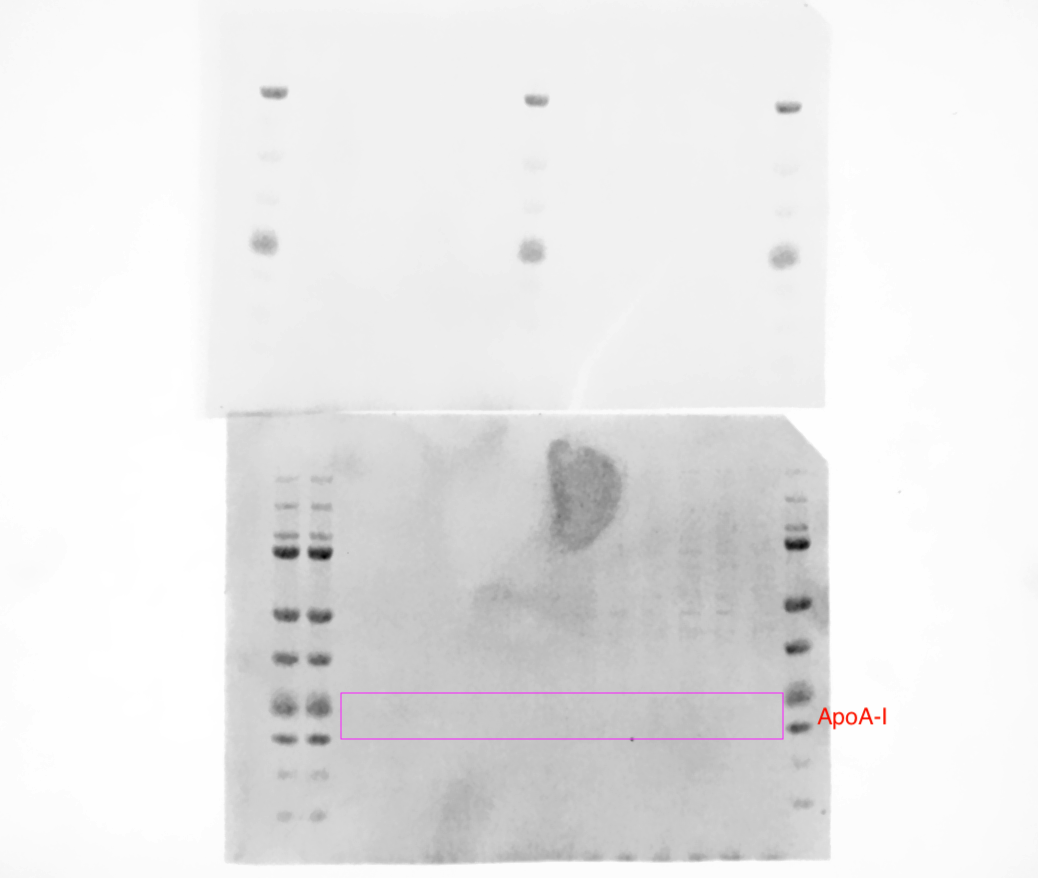

Supplement: Supplementary file 1 — Additional file 1. Raw western blot images and associated metadata. [file 12974_2024_3165_MOESM1_ESM.zip › SupplementaryFile1_rawWBimages/Fig5/KL020924_Exp247b_set1_blots1and2_round1_0000493_01/0000493_01_800_croppedBands_ApoAI.png]

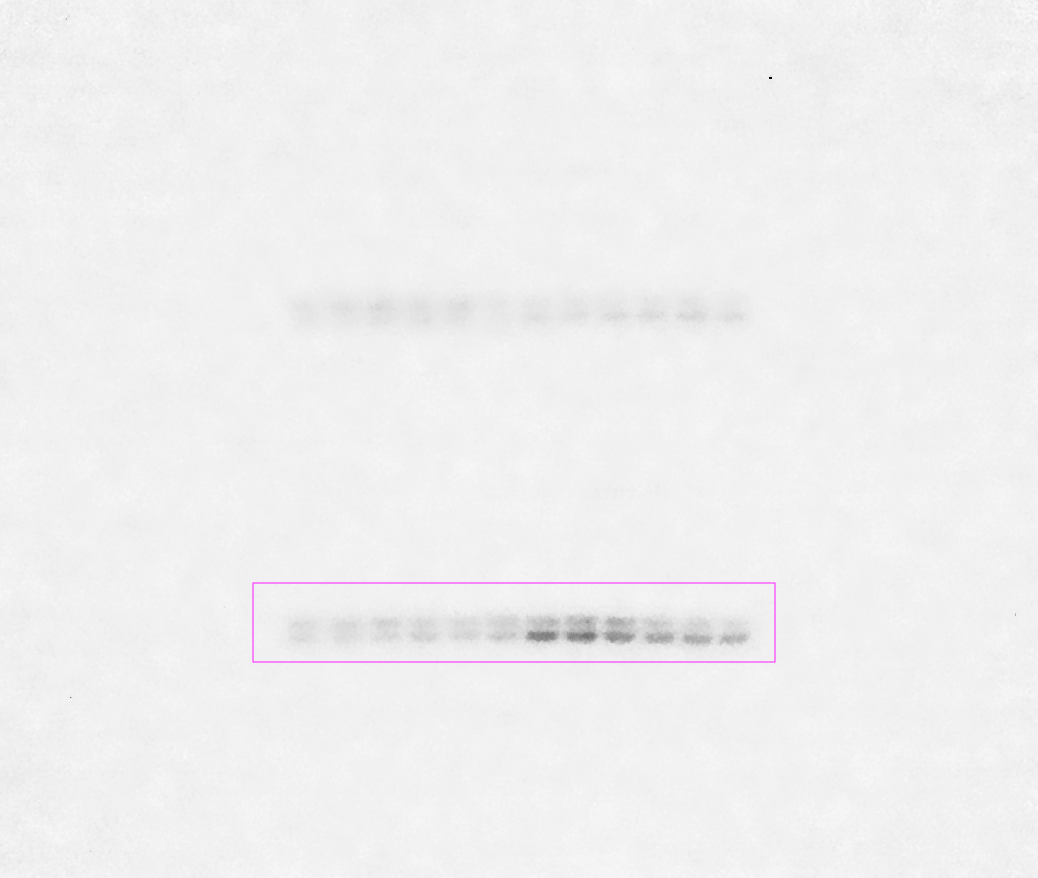

Supplement: Supplementary file 1 — Additional file 1. Raw western blot images and associated metadata. [file 12974_2024_3165_MOESM1_ESM.zip › SupplementaryFile1_rawWBimages/Fig1/LC3/0002191_01/0002191_01_Chemi_croppedBands_LC3I-II.png]

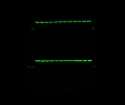

Supplement: Supplementary file 1 — Additional file 1. Raw western blot images and associated metadata. [file 12974_2024_3165_MOESM1_ESM.zip › SupplementaryFile1_rawWBimages/Fig1/LC3/0002191_01/0002191_01_TH.jpg]

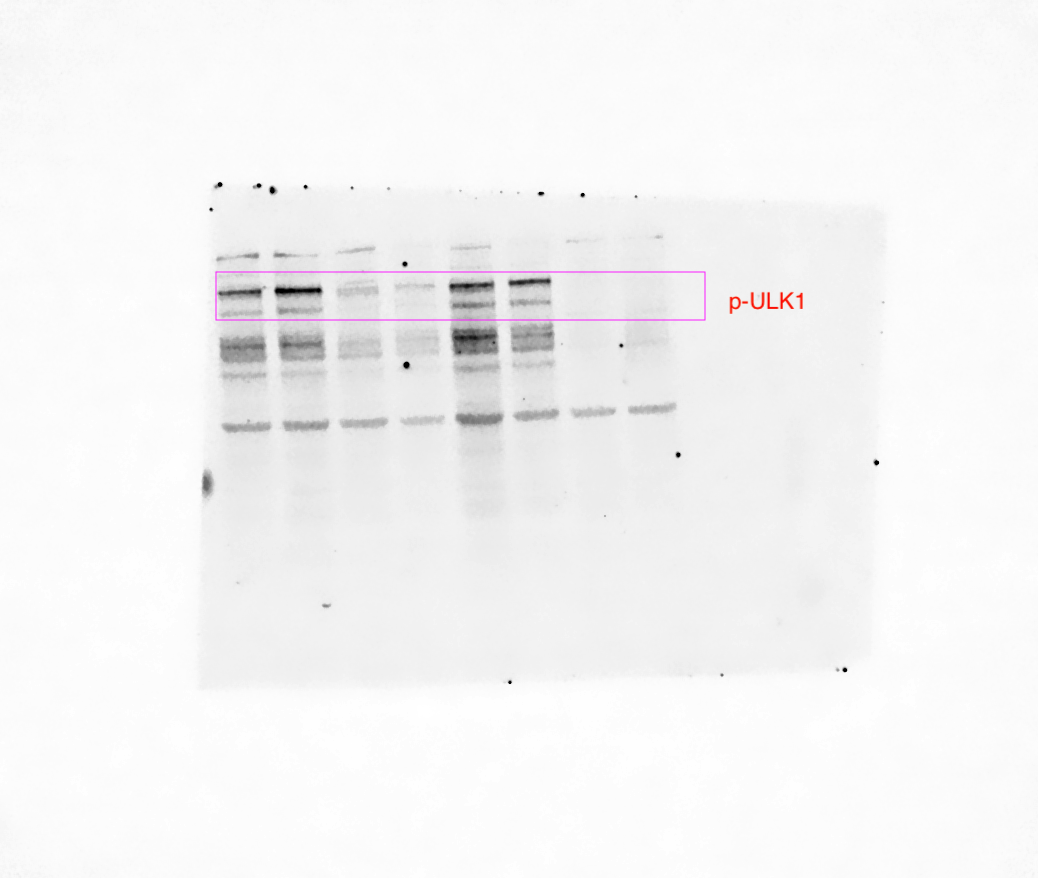

Supplement: Supplementary file 1 — Additional file 1. Raw western blot images and associated metadata. [file 12974_2024_3165_MOESM1_ESM.zip › SupplementaryFile1_rawWBimages/Fig3/mTOR_GAPDH_pULK1/0001394_01_pULK1_Rep3/0001394_01_800_croppedBands_pULK1.png]

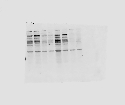

Supplement: Supplementary file 1 — Additional file 1. Raw western blot images and associated metadata. [file 12974_2024_3165_MOESM1_ESM.zip › SupplementaryFile1_rawWBimages/Fig3/mTOR_GAPDH_pULK1/0001394_01_pULK1_Rep3/0001394_01_TH.jpg]

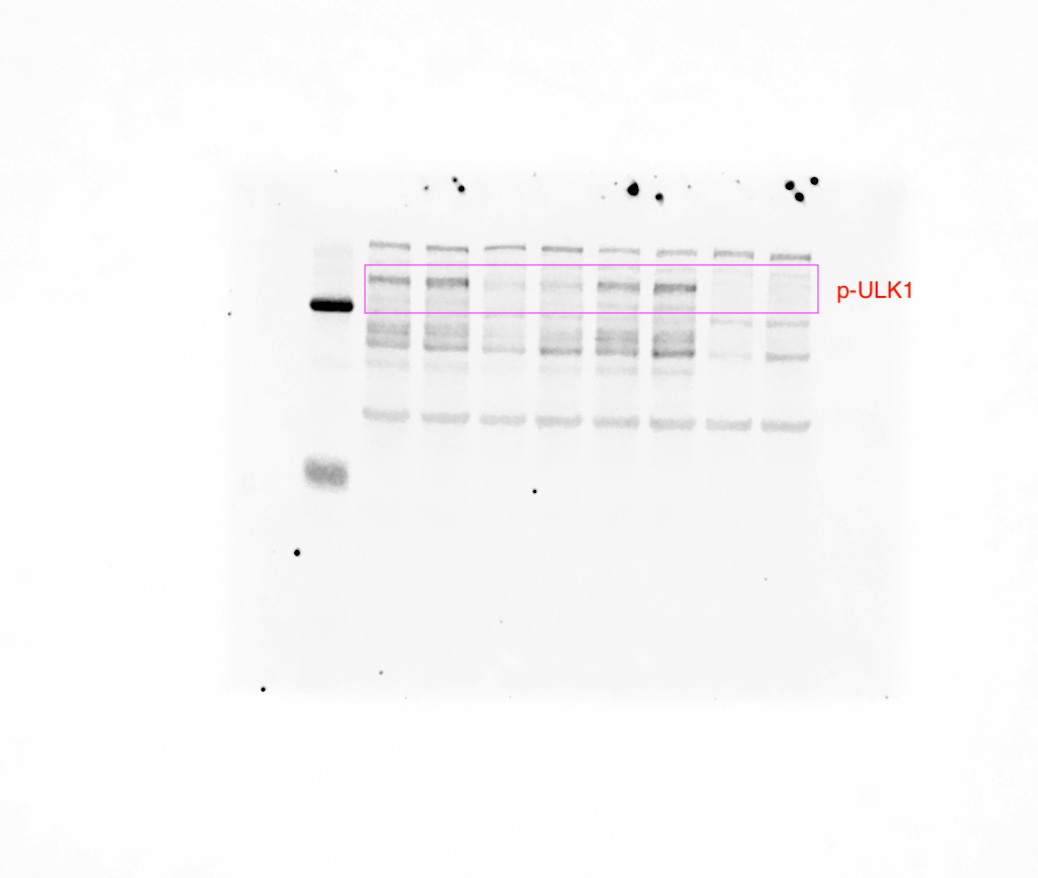

Supplement: Supplementary file 1 — Additional file 1. Raw western blot images and associated metadata. [file 12974_2024_3165_MOESM1_ESM.zip › SupplementaryFile1_rawWBimages/Fig3/mTOR_GAPDH_pULK1/0001393_01_pULK1_Rep2/0001393_01_800_croppedBands_pULK1.png]

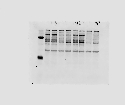

Supplement: Supplementary file 1 — Additional file 1. Raw western blot images and associated metadata. [file 12974_2024_3165_MOESM1_ESM.zip › SupplementaryFile1_rawWBimages/Fig3/mTOR_GAPDH_pULK1/0001393_01_pULK1_Rep2/0001393_01_TH.jpg]

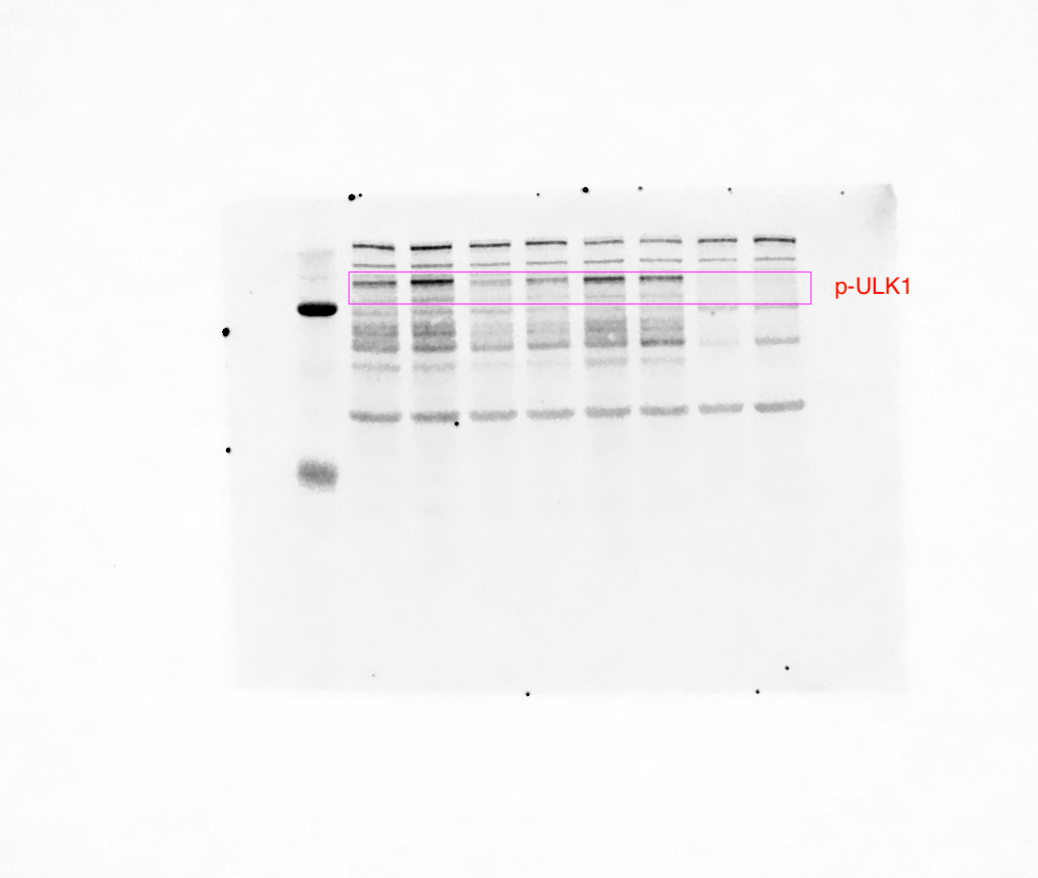

Supplement: Supplementary file 1 — Additional file 1. Raw western blot images and associated metadata. [file 12974_2024_3165_MOESM1_ESM.zip › SupplementaryFile1_rawWBimages/Fig3/mTOR_GAPDH_pULK1/0001392_01_pULK1_Rep1/0001392_01_800_croppedBands_pULK1.png]

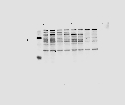

Supplement: Supplementary file 1 — Additional file 1. Raw western blot images and associated metadata. [file 12974_2024_3165_MOESM1_ESM.zip › SupplementaryFile1_rawWBimages/Fig3/mTOR_GAPDH_pULK1/0001392_01_pULK1_Rep1/0001392_01_TH.jpg]

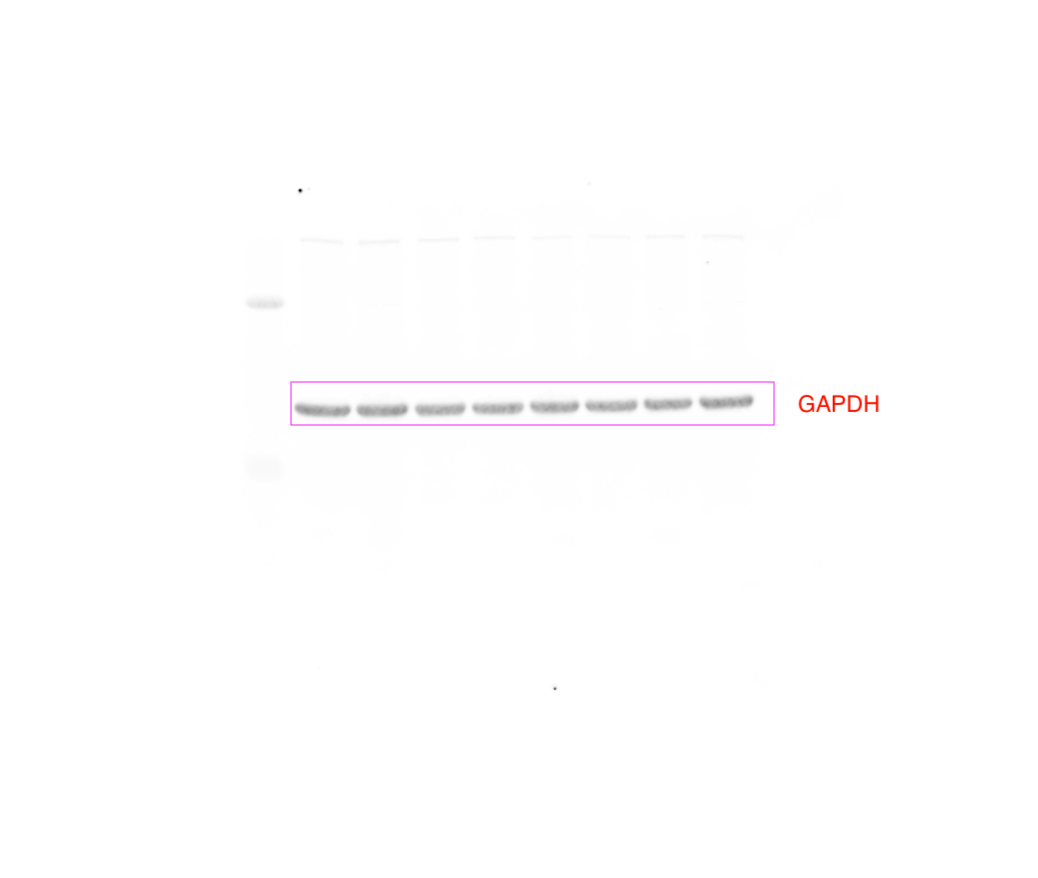

Supplement: Supplementary file 1 — Additional file 1. Raw western blot images and associated metadata. [file 12974_2024_3165_MOESM1_ESM.zip › SupplementaryFile1_rawWBimages/Fig3/mTOR_GAPDH_pULK1/0001379_01_MTOR_GADPH_Rep2/0001379_01_800_croppedBands_GAPDH.png]

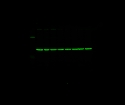

Supplement: Supplementary file 1 — Additional file 1. Raw western blot images and associated metadata. [file 12974_2024_3165_MOESM1_ESM.zip › SupplementaryFile1_rawWBimages/Fig3/mTOR_GAPDH_pULK1/0001379_01_MTOR_GADPH_Rep2/0001379_01_TH.jpg]

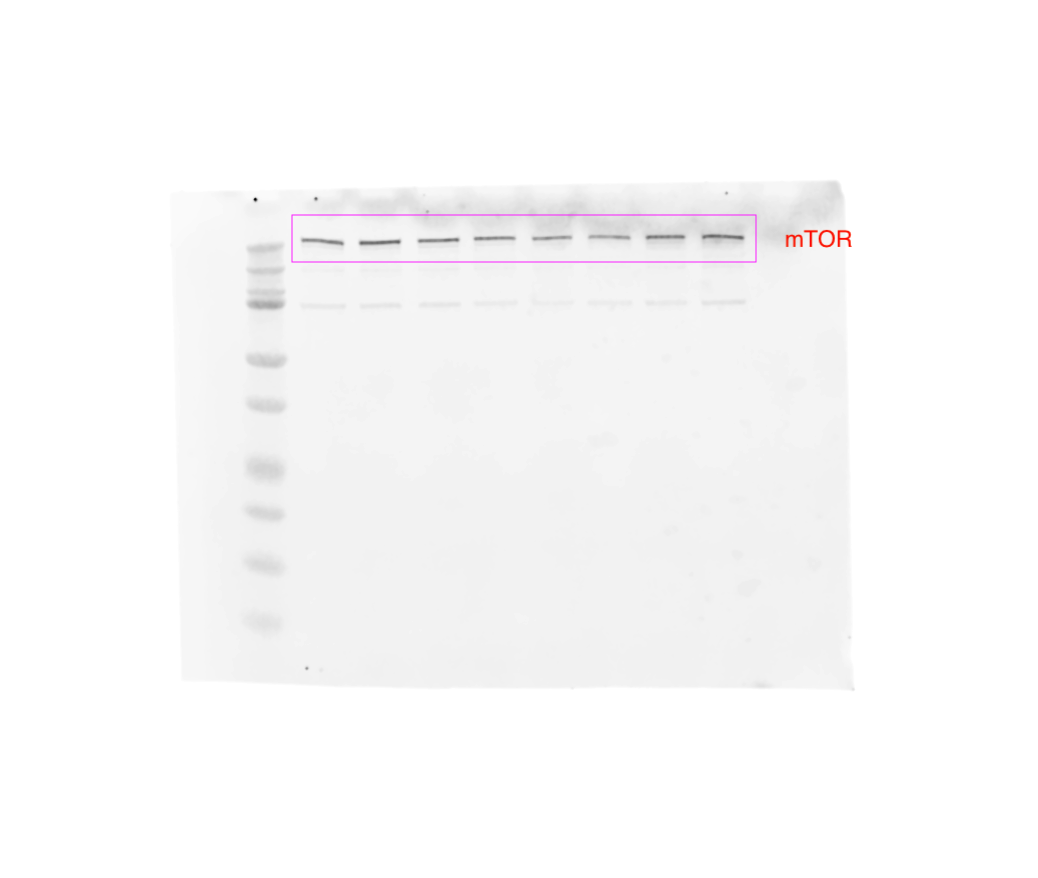

Supplement: Supplementary file 1 — Additional file 1. Raw western blot images and associated metadata. [file 12974_2024_3165_MOESM1_ESM.zip › SupplementaryFile1_rawWBimages/Fig3/mTOR_GAPDH_pULK1/0001379_01_MTOR_GADPH_Rep2/0001379_01_700_croppedBands_mTOR.png]

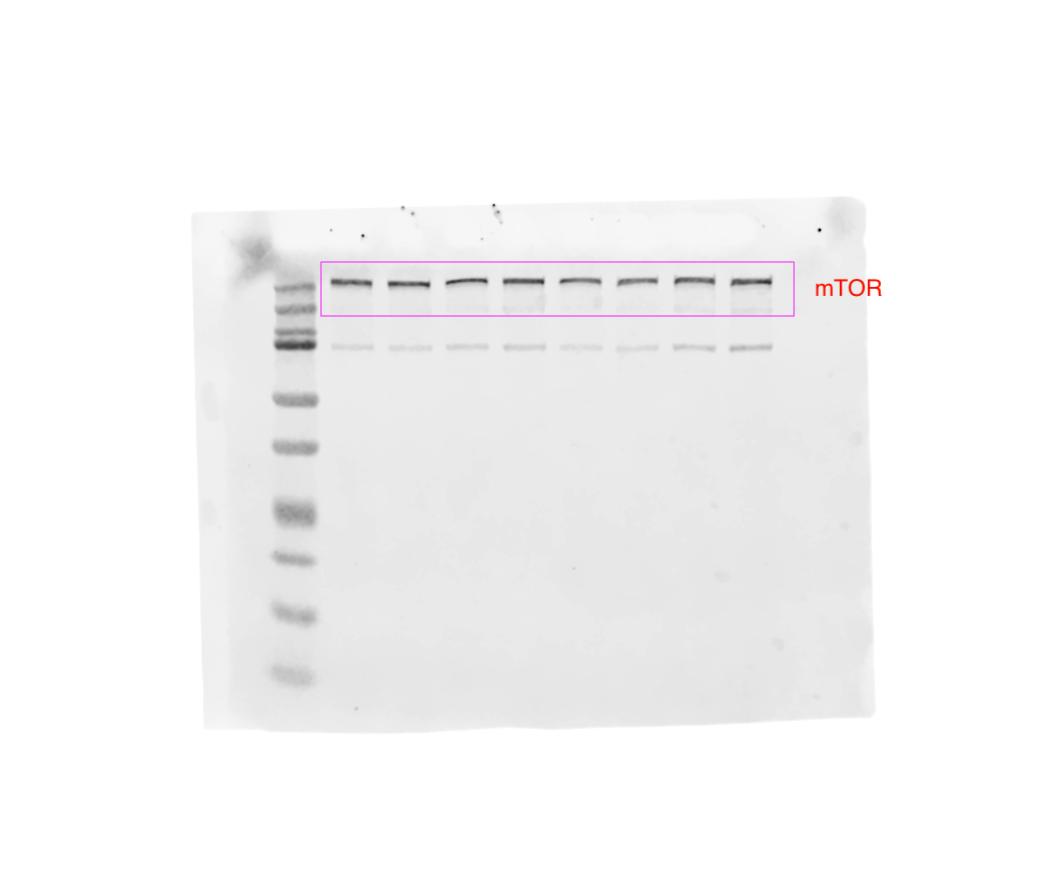

Supplement: Supplementary file 1 — Additional file 1. Raw western blot images and associated metadata. [file 12974_2024_3165_MOESM1_ESM.zip › SupplementaryFile1_rawWBimages/Fig3/mTOR_GAPDH_pULK1/0001380_01_MTOR_GAPDH_Rep1/0001380_01_700_croppedBands_mTOR.png]

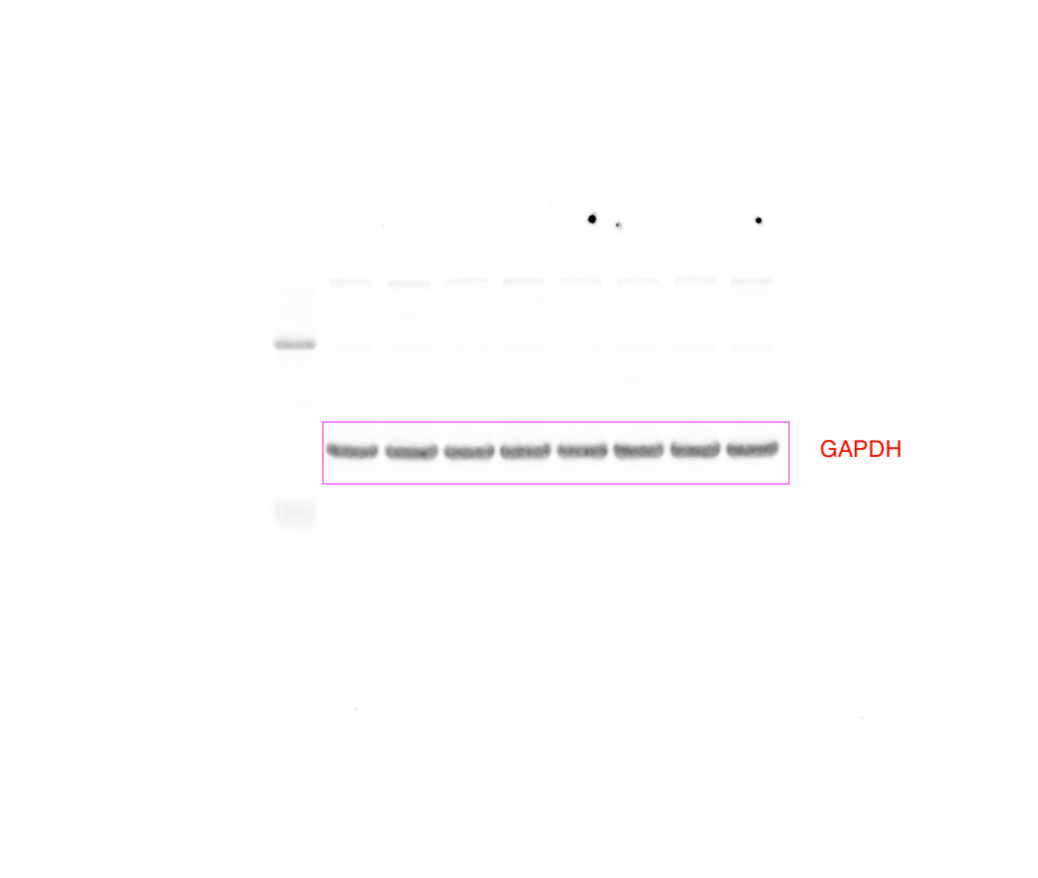

Supplement: Supplementary file 1 — Additional file 1. Raw western blot images and associated metadata. [file 12974_2024_3165_MOESM1_ESM.zip › SupplementaryFile1_rawWBimages/Fig3/mTOR_GAPDH_pULK1/0001380_01_MTOR_GAPDH_Rep1/0001380_01_800_croppedBands_GAPDH.png]

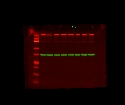

Supplement: Supplementary file 1 — Additional file 1. Raw western blot images and associated metadata. [file 12974_2024_3165_MOESM1_ESM.zip › SupplementaryFile1_rawWBimages/Fig3/mTOR_GAPDH_pULK1/0001380_01_MTOR_GAPDH_Rep1/0001380_01_TH.jpg]

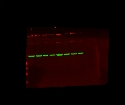

Supplement: Supplementary file 1 — Additional file 1. Raw western blot images and associated metadata. [file 12974_2024_3165_MOESM1_ESM.zip › SupplementaryFile1_rawWBimages/Fig3/mTOR_GAPDH_pULK1/0001381_01_MTOR_GAPDH_Rep3/0001381_01_TH.jpg]

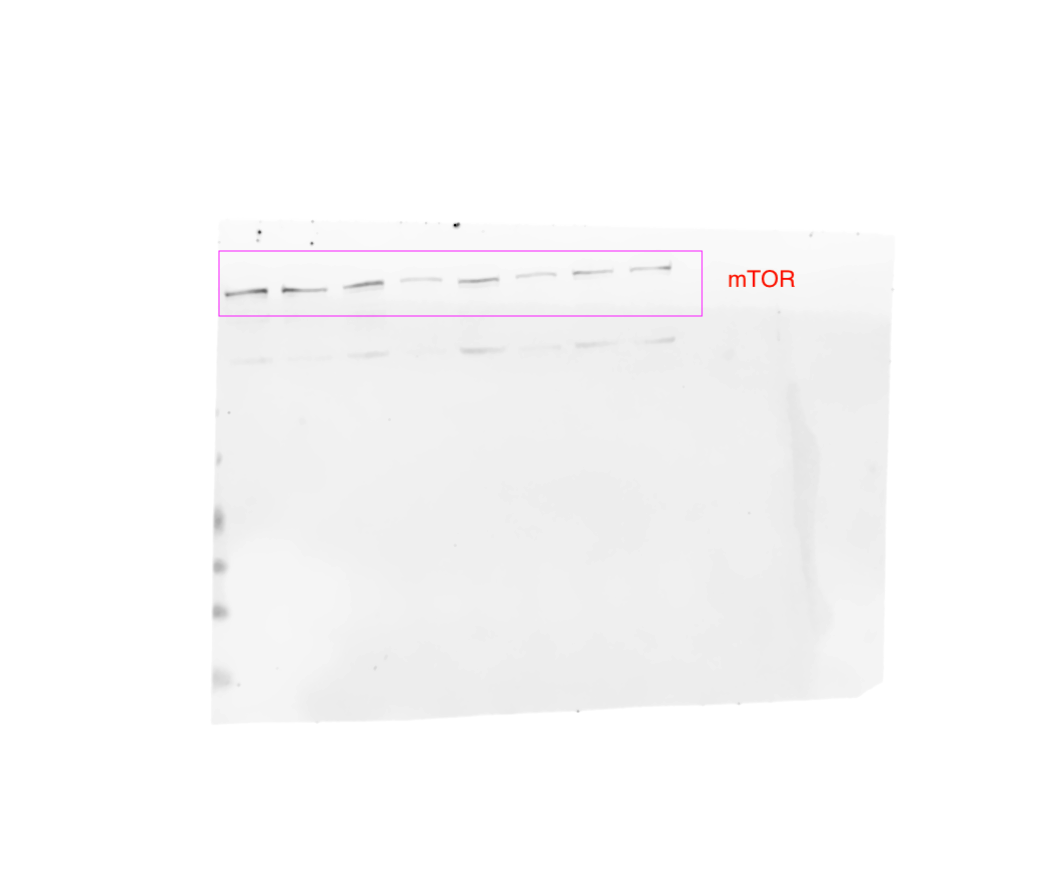

Supplement: Supplementary file 1 — Additional file 1. Raw western blot images and associated metadata. [file 12974_2024_3165_MOESM1_ESM.zip › SupplementaryFile1_rawWBimages/Fig3/mTOR_GAPDH_pULK1/0001381_01_MTOR_GAPDH_Rep3/0001381_01_700_croppedBands_mTOR.png]

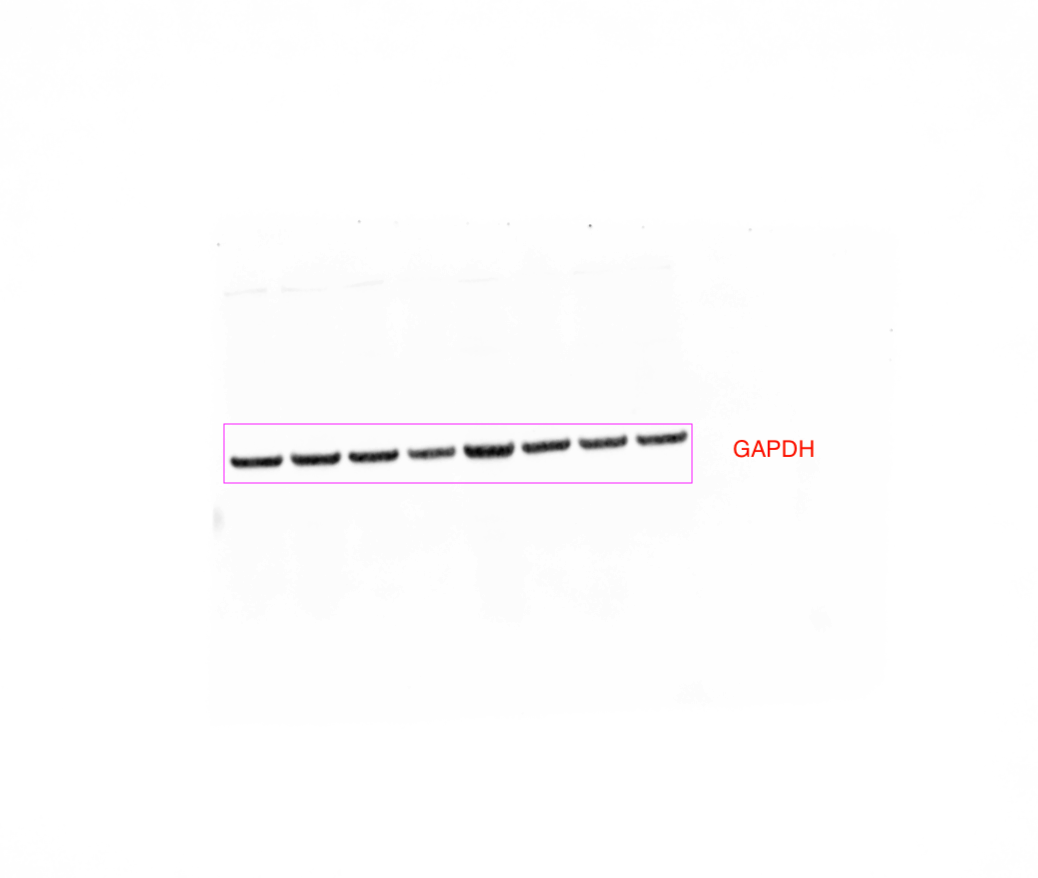

Supplement: Supplementary file 1 — Additional file 1. Raw western blot images and associated metadata. [file 12974_2024_3165_MOESM1_ESM.zip › SupplementaryFile1_rawWBimages/Fig3/mTOR_GAPDH_pULK1/0001381_01_MTOR_GAPDH_Rep3/0001381_01_800_croppedBands_GAPDH.png]

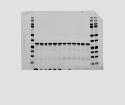

Supplement: Supplementary file 1 — Additional file 1. Raw western blot images and associated metadata. [file 12974_2024_3165_MOESM1_ESM.zip › SupplementaryFile1_rawWBimages/Fig3/pS6_S6/0001953_01/0001953_01_TH.jpg]

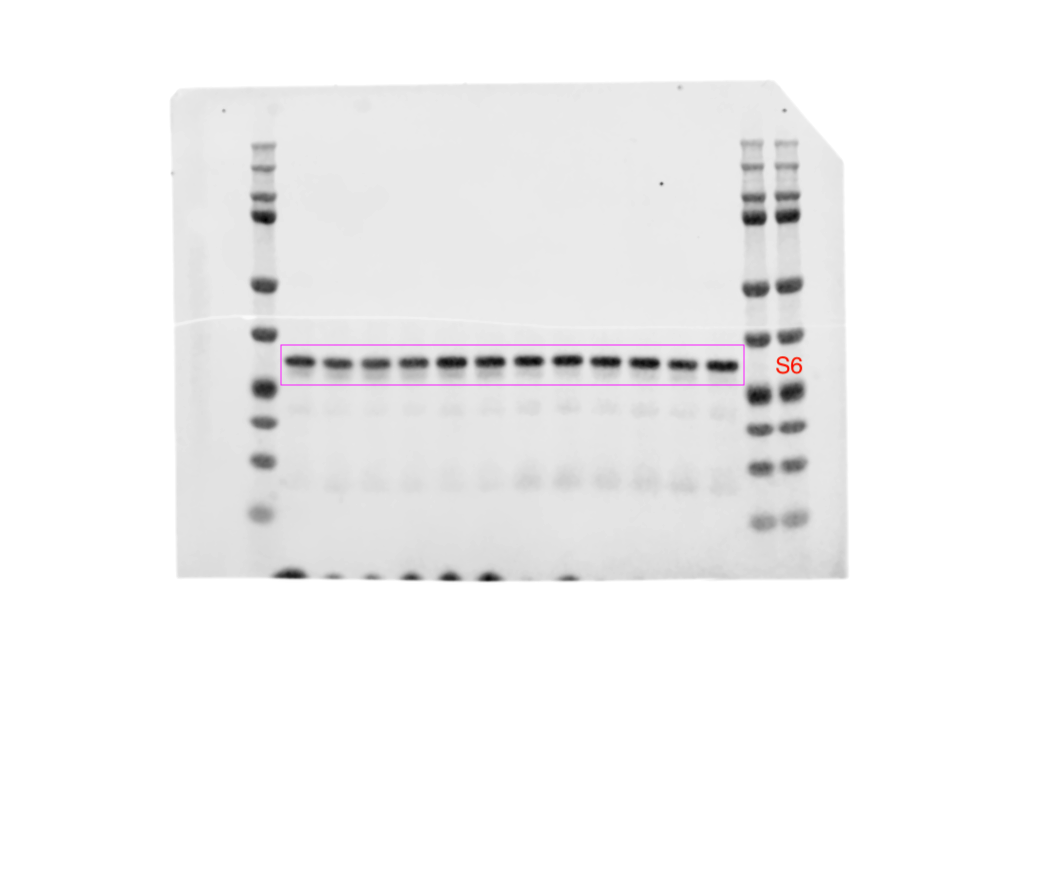

Supplement: Supplementary file 1 — Additional file 1. Raw western blot images and associated metadata. [file 12974_2024_3165_MOESM1_ESM.zip › SupplementaryFile1_rawWBimages/Fig3/pS6_S6/0001953_01/0001953_01_700_croppedBands_S6.png]

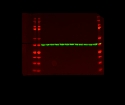

Supplement: Supplementary file 1 — Additional file 1. Raw western blot images and associated metadata. [file 12974_2024_3165_MOESM1_ESM.zip › SupplementaryFile1_rawWBimages/Fig3/pS6_S6/0001954_01/0001954_01_TH.jpg]

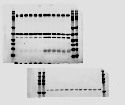

Supplement: Supplementary file 1 — Additional file 1. Raw western blot images and associated metadata. [file 12974_2024_3165_MOESM1_ESM.zip › SupplementaryFile1_rawWBimages/Fig3/pS6_S6/0001940_01/0001940_01_TH.jpg]

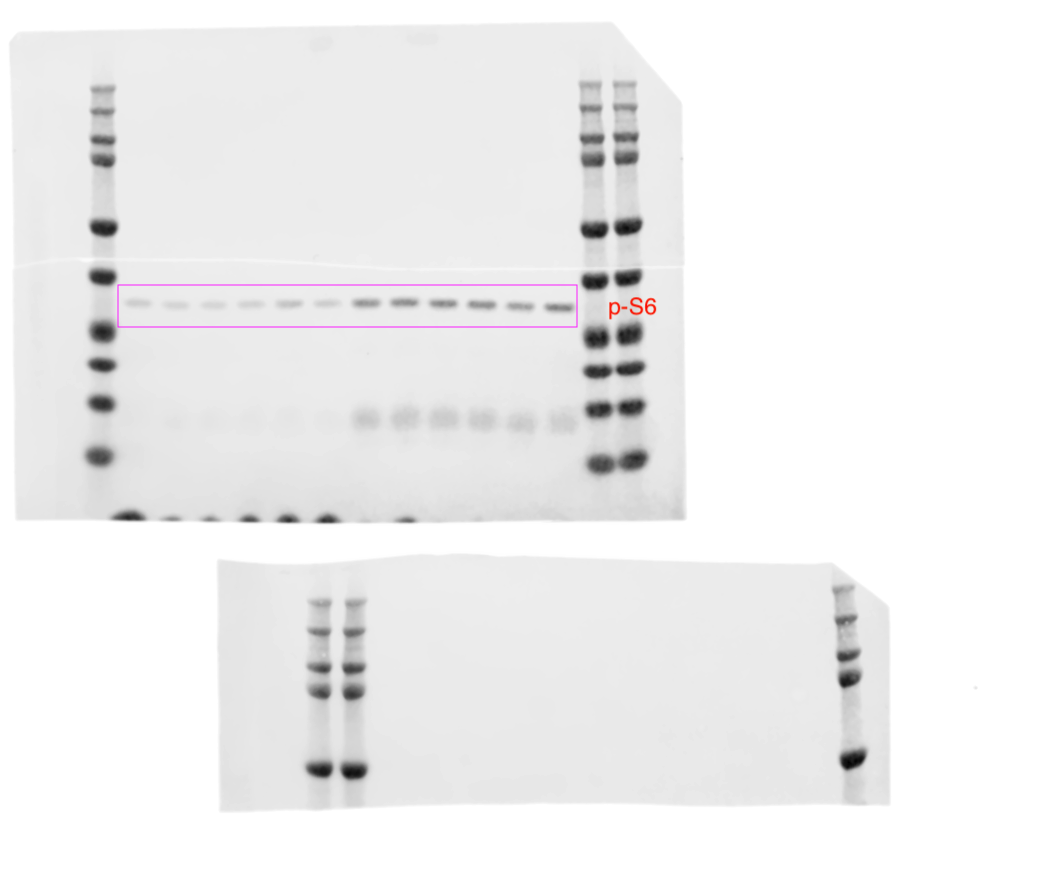

Supplement: Supplementary file 1 — Additional file 1. Raw western blot images and associated metadata. [file 12974_2024_3165_MOESM1_ESM.zip › SupplementaryFile1_rawWBimages/Fig3/pS6_S6/0001940_01/0001940_01_700_croppedBands_pS6.png]

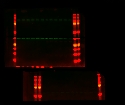

Supplement: Supplementary file 1 — Additional file 1. Raw western blot images and associated metadata. [file 12974_2024_3165_MOESM1_ESM.zip › SupplementaryFile1_rawWBimages/Fig3/pS6_S6/0001941_01/0001941_01_TH.jpg]

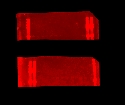

Supplement: Supplementary file 1 — Additional file 1. Raw western blot images and associated metadata. [file 12974_2024_3165_MOESM1_ESM.zip › SupplementaryFile1_rawWBimages/Fig3/pS6_S6/0001955_01/0001955_01_TH.jpg]

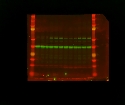

Supplement: Supplementary file 1 — Additional file 1. Raw western blot images and associated metadata. [file 12974_2024_3165_MOESM1_ESM.zip › SupplementaryFile1_rawWBimages/Fig3/pAKT_AKT/0002120_01/0002120_01_TH.jpg]

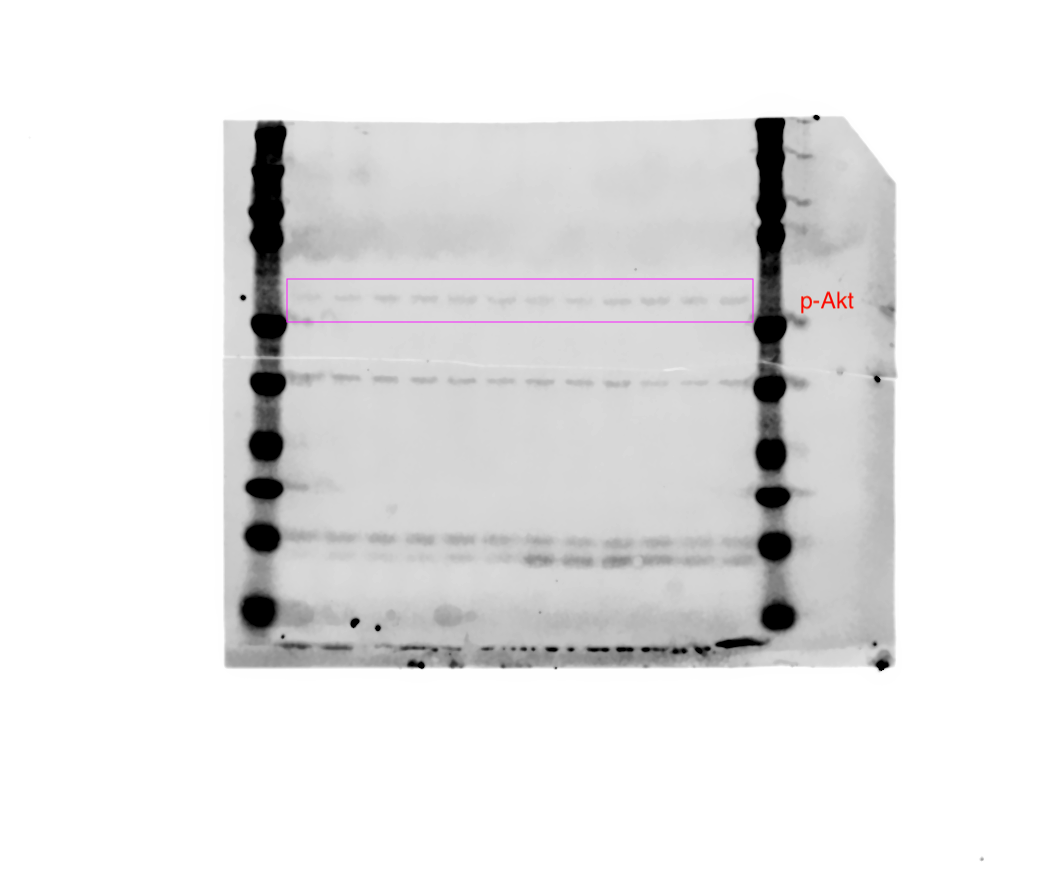

Supplement: Supplementary file 1 — Additional file 1. Raw western blot images and associated metadata. [file 12974_2024_3165_MOESM1_ESM.zip › SupplementaryFile1_rawWBimages/Fig3/pAKT_AKT/0002120_01/0002120_01_700_croppedBands_pAKT.png]

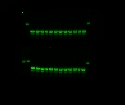

Supplement: Supplementary file 1 — Additional file 1. Raw western blot images and associated metadata. [file 12974_2024_3165_MOESM1_ESM.zip › SupplementaryFile1_rawWBimages/Fig3/pAKT_AKT/0002127_01/0002127_01_TH.jpg]

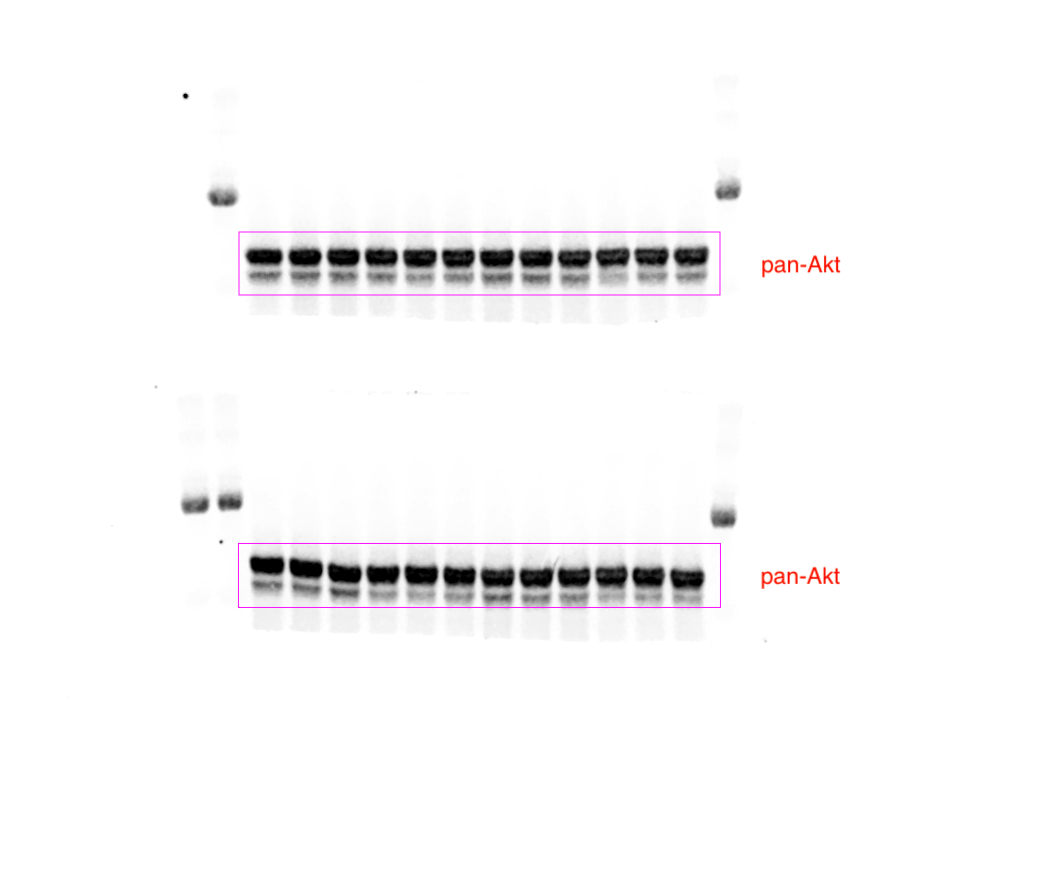

Supplement: Supplementary file 1 — Additional file 1. Raw western blot images and associated metadata. [file 12974_2024_3165_MOESM1_ESM.zip › SupplementaryFile1_rawWBimages/Fig3/pAKT_AKT/0002127_01/0002127_01_800_croppedBands_panAKT.png]

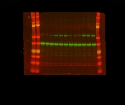

Supplement: Supplementary file 1 — Additional file 1. Raw western blot images and associated metadata. [file 12974_2024_3165_MOESM1_ESM.zip › SupplementaryFile1_rawWBimages/Fig3/pAKT_AKT/0002122_01/0002122_01_TH.jpg]

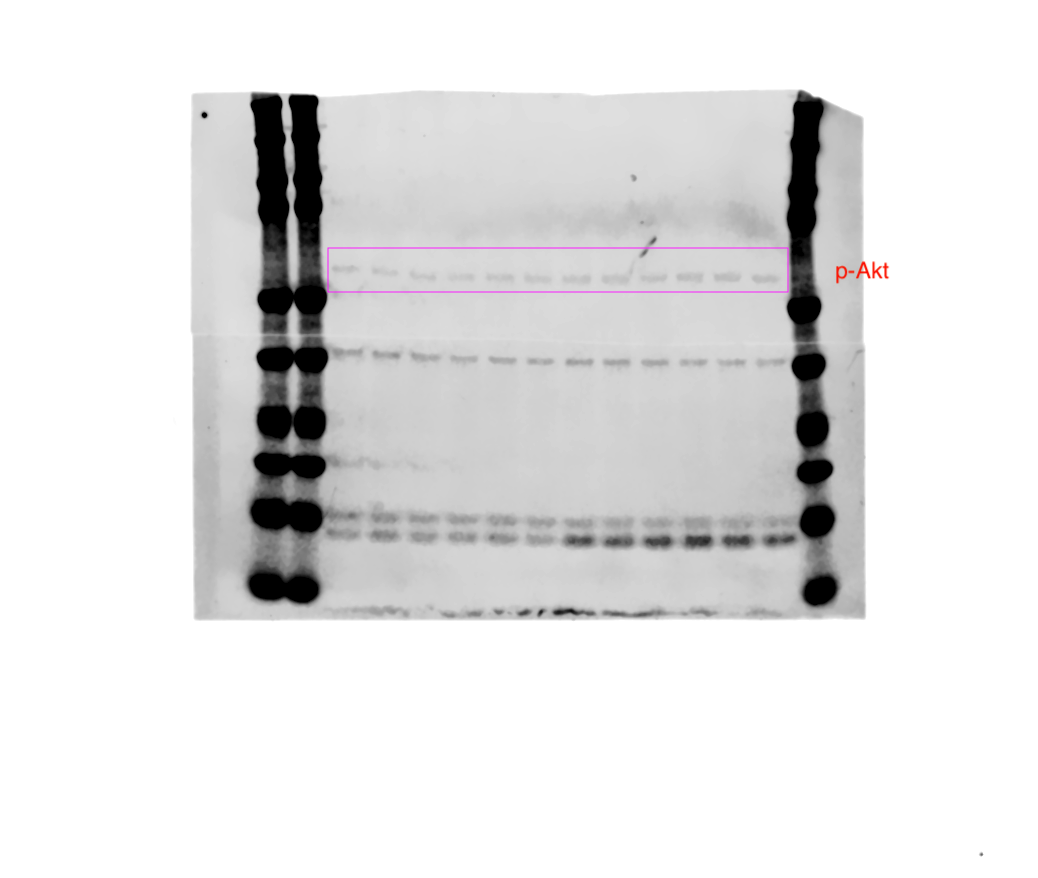

Supplement: Supplementary file 1 — Additional file 1. Raw western blot images and associated metadata. [file 12974_2024_3165_MOESM1_ESM.zip › SupplementaryFile1_rawWBimages/Fig3/pAKT_AKT/0002122_01/0002122_01_700_croppedBands_pAKT.png]

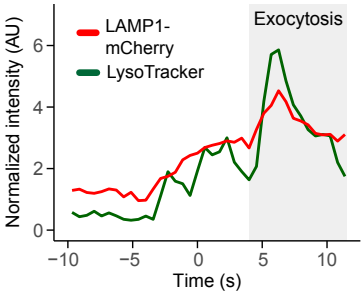

Supplement: Supplementary file 2 — Additional file 2. TIRF microscopy movie of LysoTracker Green-loaded iAstrocytes expressing LAMP1-mCherry. [file 12974_2024_3165_MOESM2_ESM.zip › SupplementaryFile2_TIRFmovies/movie2_quantification.pdf]

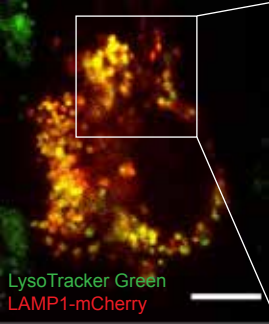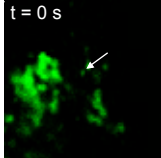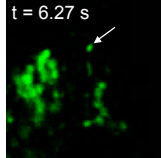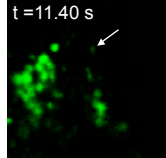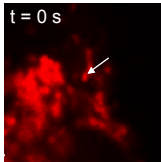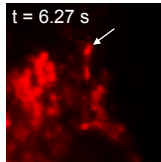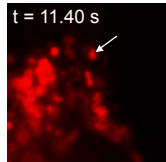

Supplement: Supplementary file 2 — Additional file 2. TIRF microscopy movie of LysoTracker Green-loaded iAstrocytes expressing LAMP1-mCherry. [file 12974_2024_3165_MOESM2_ESM.zip › SupplementaryFile2_TIRFmovies/movie2_selectedFrames.pdf]
