# Supplementary material for: mTOR activation induces endolysosomal remodeling and nonclassical secretion of IL-32 via exosomes in inflammatory reactive astrocytes
Source: J Neuroinflammation. 2024 Aug 8;21:198. doi: 10.1186/s12974-024-03165-w (PMC11312292; doi:10.1186/s12974-024-03165-w)
Supplement: Supplementary file 7 — Additional file 7: Supplementary Figures. [file 12974_2024_3165_MOESM7_ESM.docx]

**
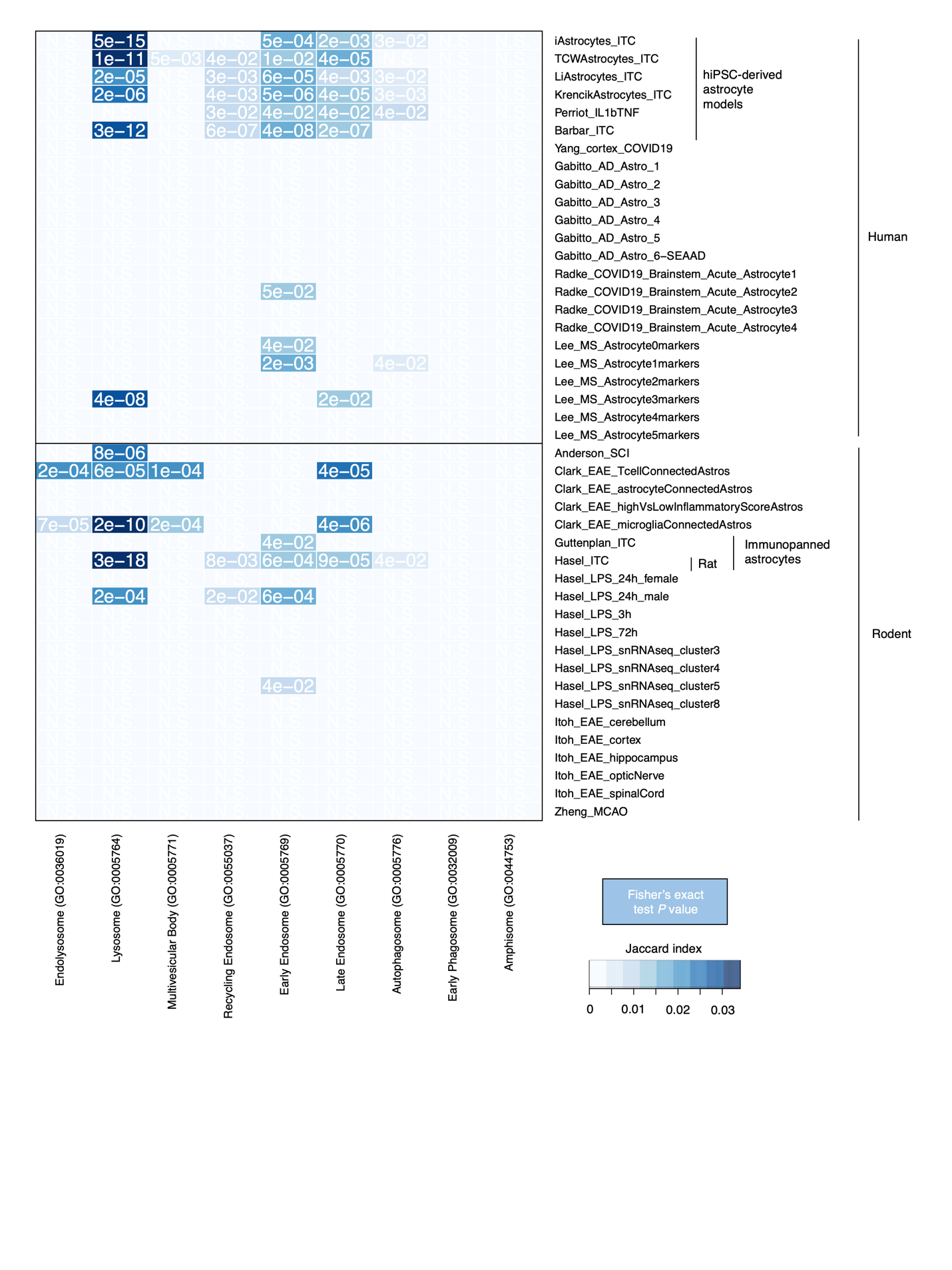
**

**Supplementary figure 1 | Overlap analysis of downregulated genes with endolysosomal-related terms in inflammatory reactive astrocytes across different contexts and species**. GO Cellular Component terms corresponding to different components of the endolysosomal system were analyzed for overlap with downregulated genes in human or rodent astrocytes (all from mouse except where noted) found in various neuroinflammatory models and conditions. See Supplementary Table 3 for metadata corresponding to the studies analyzed. *P* values were adjusted for multiple comparisons with the Benjamini-Hochberg procedure.

**
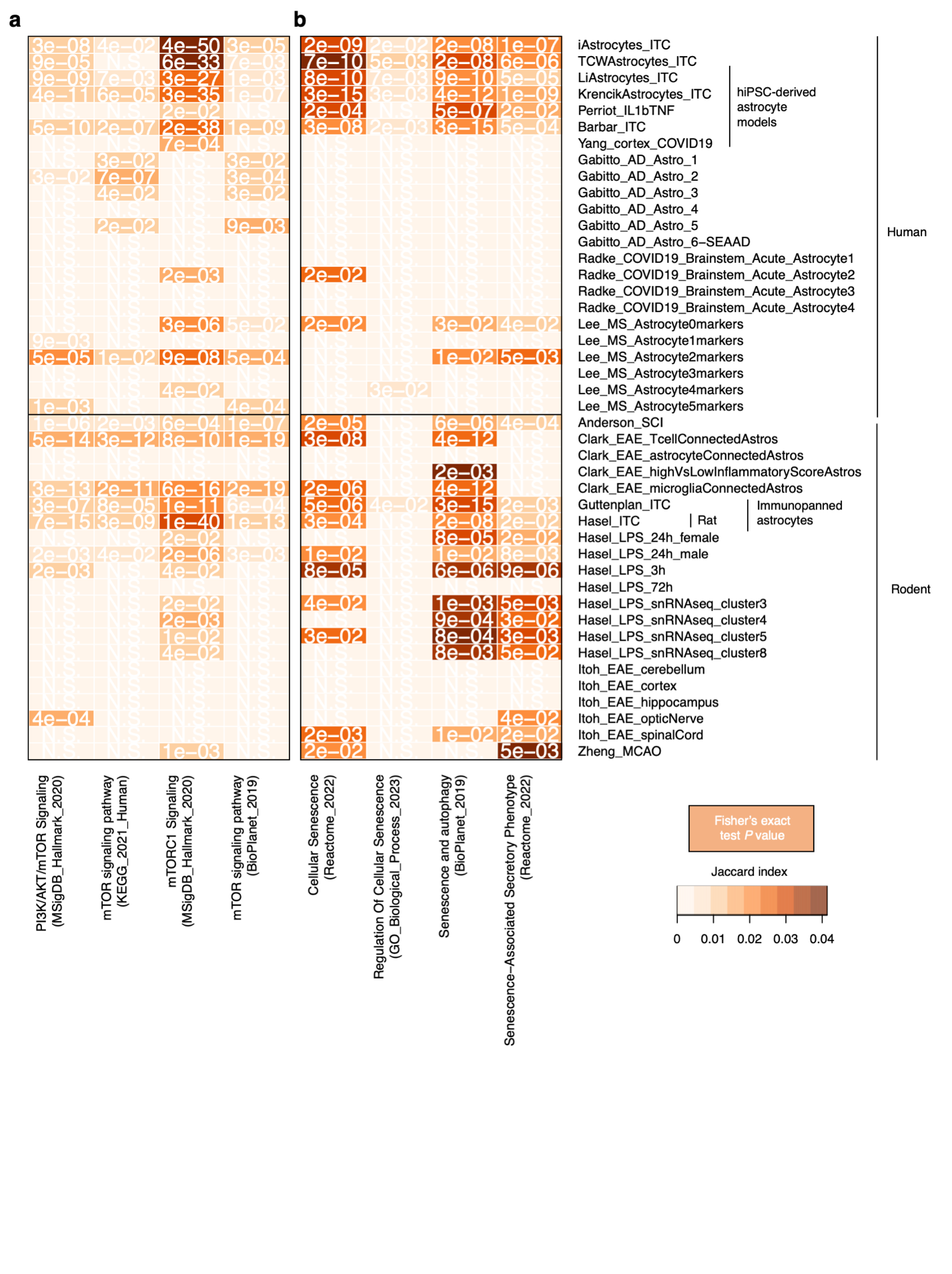
**

**Supplementary figure 2 | Overlap analysis of upregulated in inflammatory reactive astrocytes across different contexts and species**. mTOR-related terms (**a**) or cellular senescence-related terms (**b**) were analyzed for overlap with upregulated genes in human or rodent astrocytes (all from mouse except where noted) found in various neuroinflammatory models and conditions. See Table S3 (Additional file 5) for metadata corresponding to the studies analyzed. *P* values were adjusted for multiple comparisons with the Benjamini-Hochberg procedure.

**
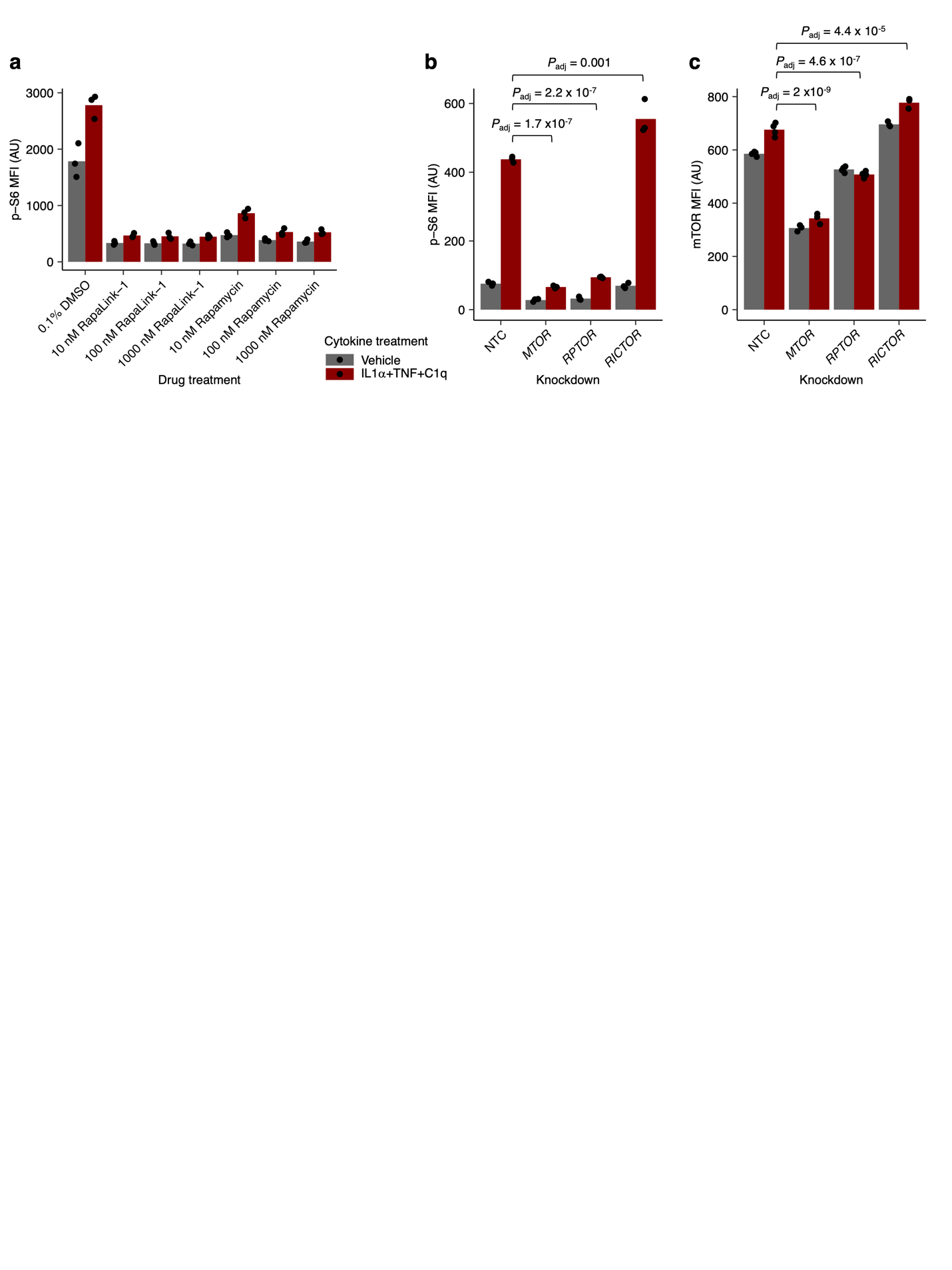
**

**Supplementary figure 3 | Validation of mTOR inhibition and knockdown**. **a**, Median fluorescence intensity (MFI) of phospho-S6 measured by flow cytometry in ITC- vs. vehicle-treated iAstrocytes co-treated with rapamycin or Rapalink-1. **b**-**c**, Phospho-S6 (**b**) or mTOR (**c**) MFI measured by flow cytometry in vehicle- or ITC-treated iAstrocytes transduced with a non-targeting sgRNA (NTC) or sgRNAs targeting genes encoding mTORC1/2 subunits; n = 3 wells for all conditions; *P* values calculated by linear regression with Holm’s correction for multiple testing, shown for selected comparisons).

**
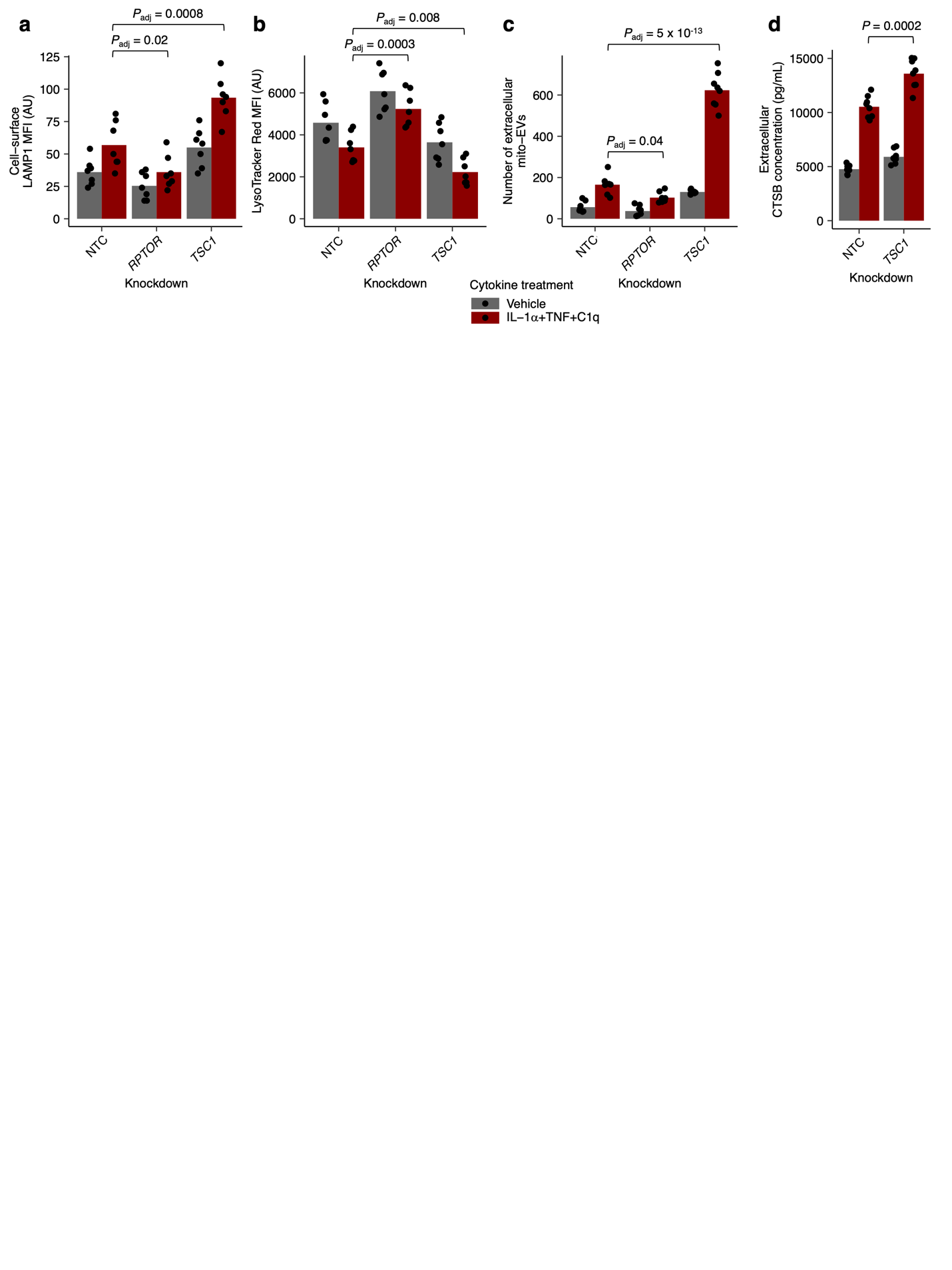
**

**Supplementary figure 4 | Modulation of mTORC1 activity affects endolysosomal exocytosis bidirectionally**. **a**-**d**, Median fluorescence intensity (MFI) of cell-surface LAMP1 (**a**) or LysoTracker (**b**) staining measured by flow cytometry, abundance of mito-EVs in conditioned media (**c**), or extracellular CTSB concentration measured by electrochemiluminescence-based immunoassay (**d**) in ITC- vs. vehicle-treated iAstrocytes transduced with non-targeting (NTC) sgRNAs or sgRNAs targeting *RPTOR* or *TSC1* (n = 7 wells per condition for LAMP1 and LysoTracker data, n = 8 wells per condition for mito-EV and CTSB data; *P* values calculated by linear regression with Holm’s correction for multiple testing, shown only if significant).

**
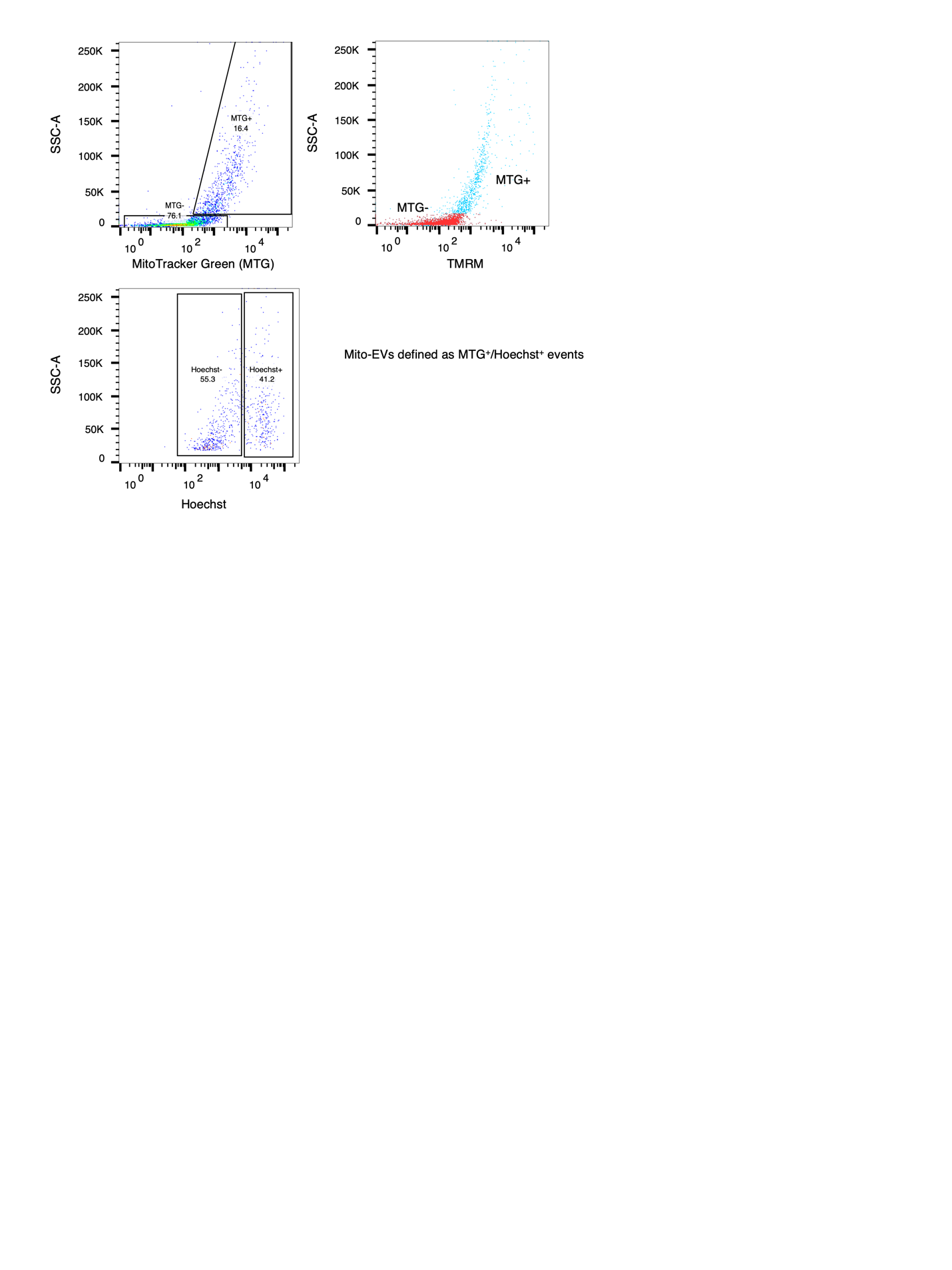
**

**Supplementary figure 5 | Gating strategy for quantifying mito-EVs from iAstrocyte conditioned media**. Events were first gated on MitoTracker Green fluorescence, with MTG^+^ events subsequently gated by Hoechst staining. Mito-EVs were defined as MTG^+^/Hoechst^+^ events.

**
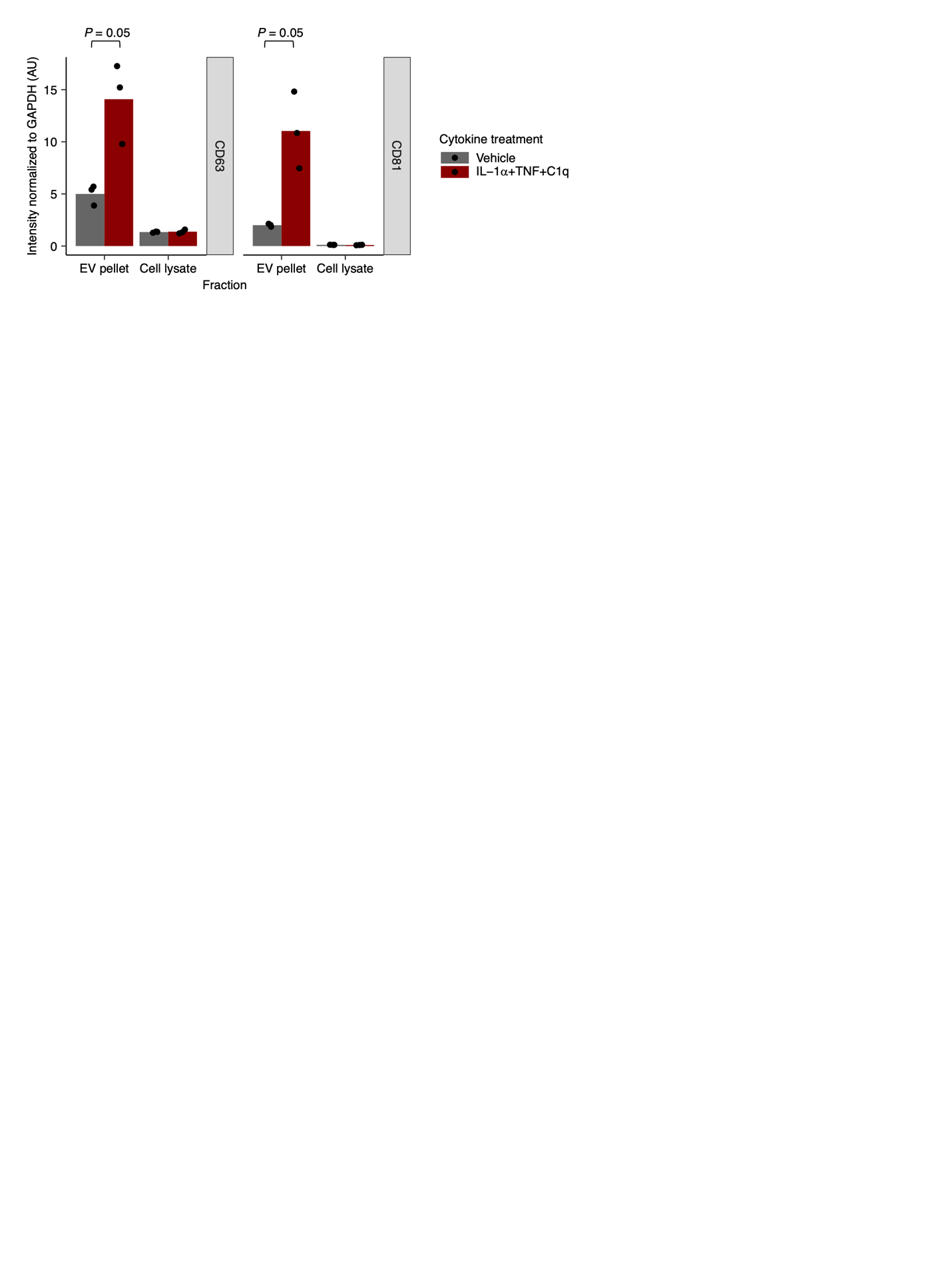
**

**Supplementary figure 6 | ITC-treated astrocytes release more extracellular vesicles**. Quantification of CD63 or CD81 immunoblot bands from total cell lysate or extracellular vesicles from ITC- vs. vehicle-treated iAstrocytes shown in Fig. 5a (n = 3 wells per condition; *P* values by two-sided Student’s t test).

**
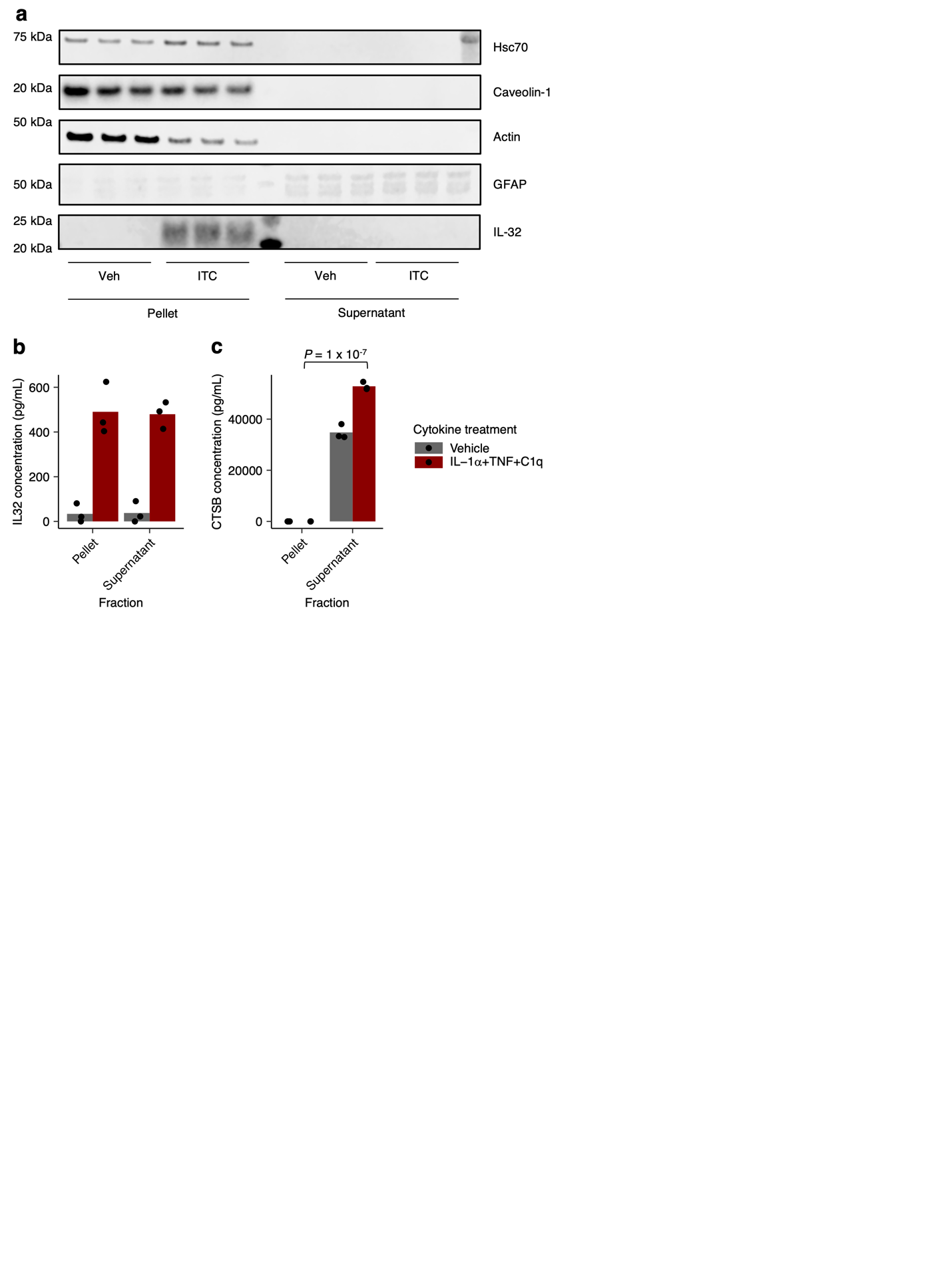
**

**Supplementary figure 7 | Partition analysis of IL-32 or CTSB**. Concentration of IL-32 measured by ELISA (**a**) or CTSB measured by electrochemiluminescence-based immunoassay (**b**) in resuspended extracellular vesicle pellets (lysed with detergent) vs the supernatant (see Methods); n = 3 per condition, *P* values calculated only for ITC-treated conditions by two-sided Student’s t test, shown only when significant.

**
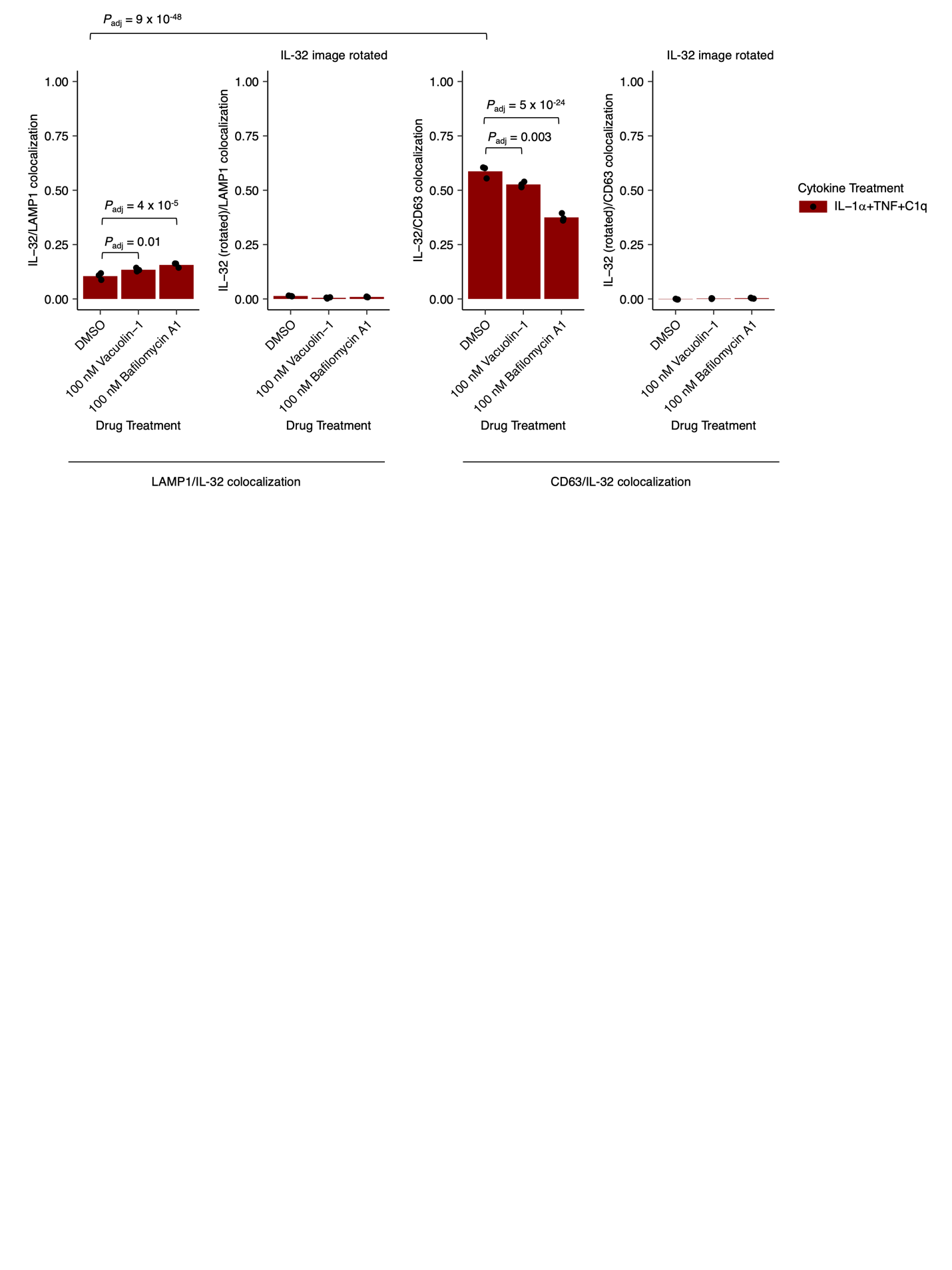
**

**Supplementary figure 8 | Colocalization analysis of IL-32 with LAMP1 or CD63**. Pearson’s colocalization coefficient between IL-32 and LAMP1 (left) or between IL-32 and CD63 (right) in ITC-treated iAstrocytes co-treated with vacuolin-1 or bafilomycin A1, corresponding to images from the immunostaining experiment shown in Fig. 5d (n = 3 wells per condition; *P* values calculated by beta regression with correction for multiple testing by Holm’s method, shown only if significant).

**
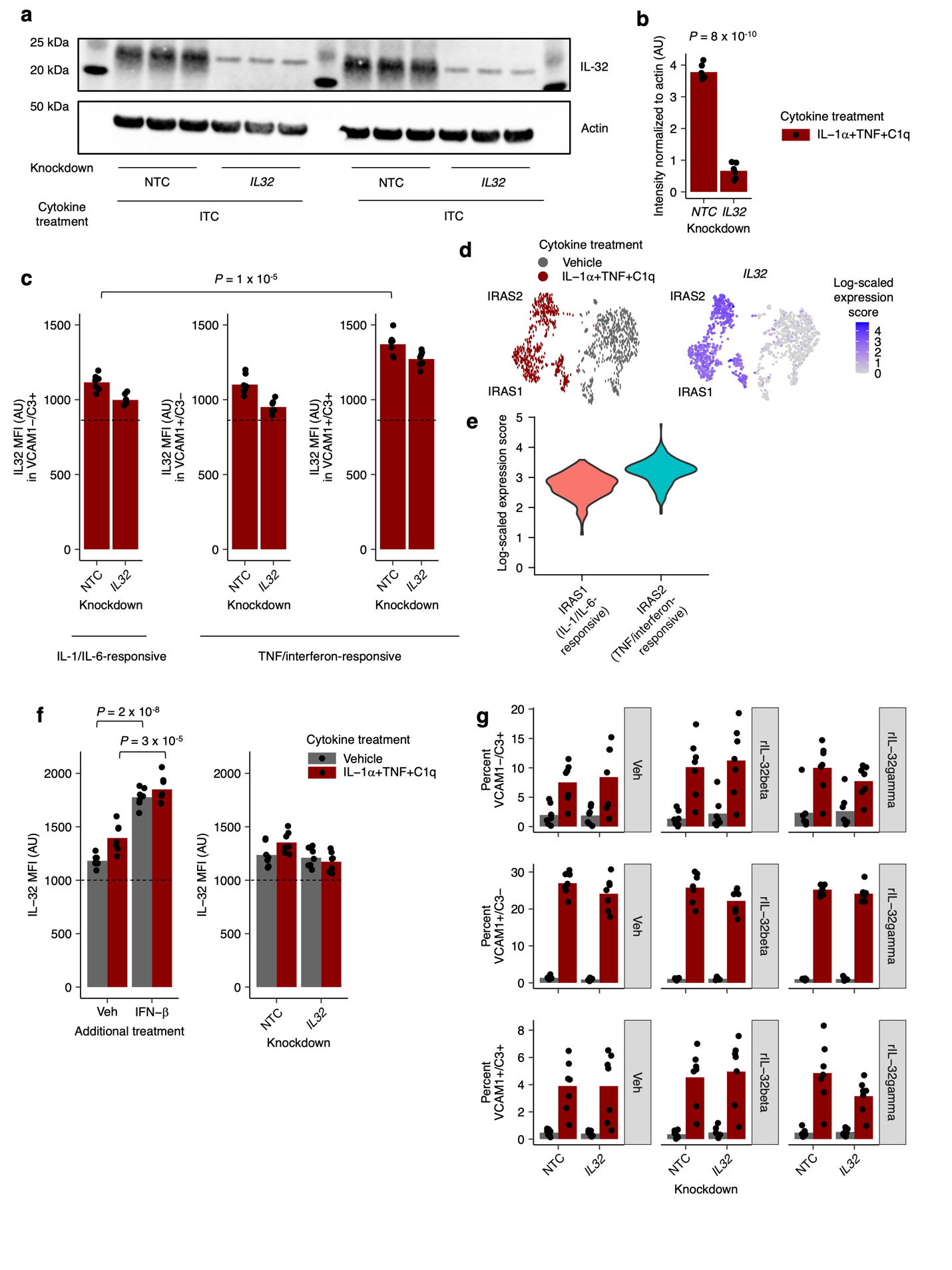
**

**Supplementary Figure 9 (legend overleaf)**

**Supplementary Figure 9 | IL-32 levels are higher in interferon/TNF-responsive inflammatory reactive astrocytes and after treatment with IFN-β**. **a**, Immunoblot against IL-32 in ITC-treated iAstrocytes transduced with non-targeting (NTC) sgRNAs or sgRNAs targeting *IL32.* **b**, Quantification of IL-32 levels from immunoblot shown in **a**; n = 6 wells per condition. **c**, Median fluorescence intensity (MFI) of IL-32 in IL-1/IL-6-responsive (VCAM1-/C3+) or TNF/interferon-responsive (VCAM1+/C3-, VCAM1+/C3+) inflammatory reactive astrocytes as defined in Leng *et al.* [9]; n = 7 wells per condition. **d**-**e**, *IL32* transcript levels from single-cell RNA-seq data on ITC- vs. vehicle-treated iAstrocytes from Leng *et al.* [9] visualized on uniform manifold approximation embedding (**d**) or as violin plots (**e**). **f**, IL-32 MFI in ITC- vs. vehicle-treated iAstrocytes treated concurrently with vehicle or IFN-β transduced with non-targeting (NTC) sgRNAs or sgRNAs targeting *IL32*; n = 7 wells per condition. g, Proportion of VCAM1-/C3+, VCAM1+/C3-, or VCAM1+/C3+ iAstrocytes after vehicle vs. ITC treatment with or without IL-32 knockdown or treatment with free recombinant IL-32β or IL-32𝛾 (200 ng/mL); n = 7 wells per condition. Dotted lines in **c** and **f** reflect estimated level of non-specific staining based on degree of IL-32 knockdown from **b.** *P* values where shown were calculated by two-sided Student’s t test.

**
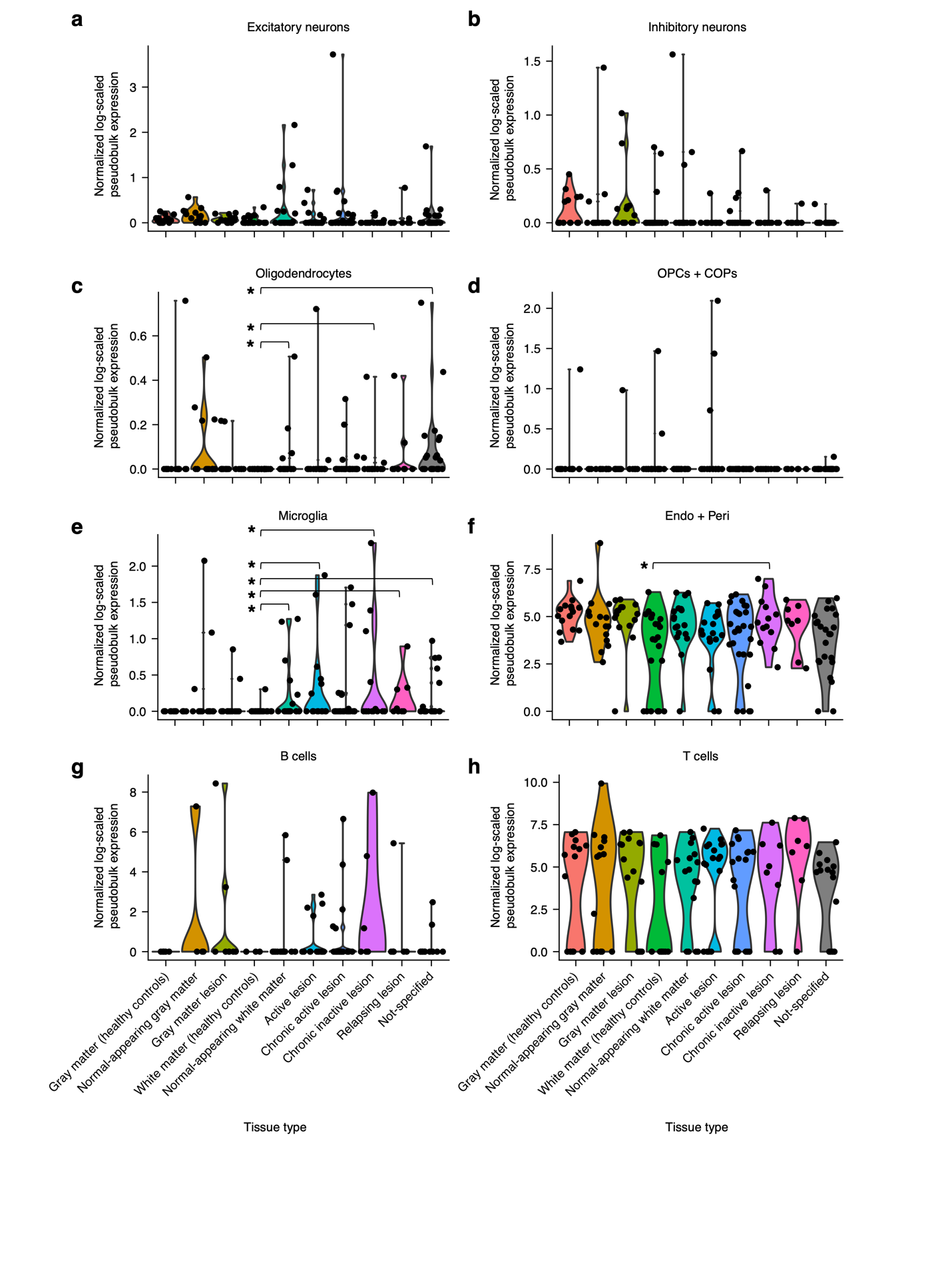
**

**Supplementary Figure 10 | *IL32* expression in non-astrocyte cell types in snRNA-seq data from Macnair *et al****.* **a**-**f**, Log-scaled normalized pseudobulk counts of *IL32* in excitatory neurons (**a**), inhibitory neurons (**b**), oligodendrocytes (**c**), oligodendrocyte precursor cells (OPCs) and differentiation-committed oligodendrocyte precursors (COPs) (**d**), microglia (**e**), or endothelial cells (endo) and pericytes (peri) (**f**). *P* values were calculated with the Mann-Whitney U test and were not adjusted for multiple comparisons; * *P* < 0.05. n = 15 for healthy control gray matter, n = 15 for normal-appearing gray matter, n = 15 for gray matter lesion, n = 22 for healthy control white matter, n = 18 for normal-appearing white matter, n = 17 for active lesion, n = 27 for chronic active lesion, n = 13 for chronic inactive lesion, n = 8 for relapsing lesion, n = 23 for not specified.

**
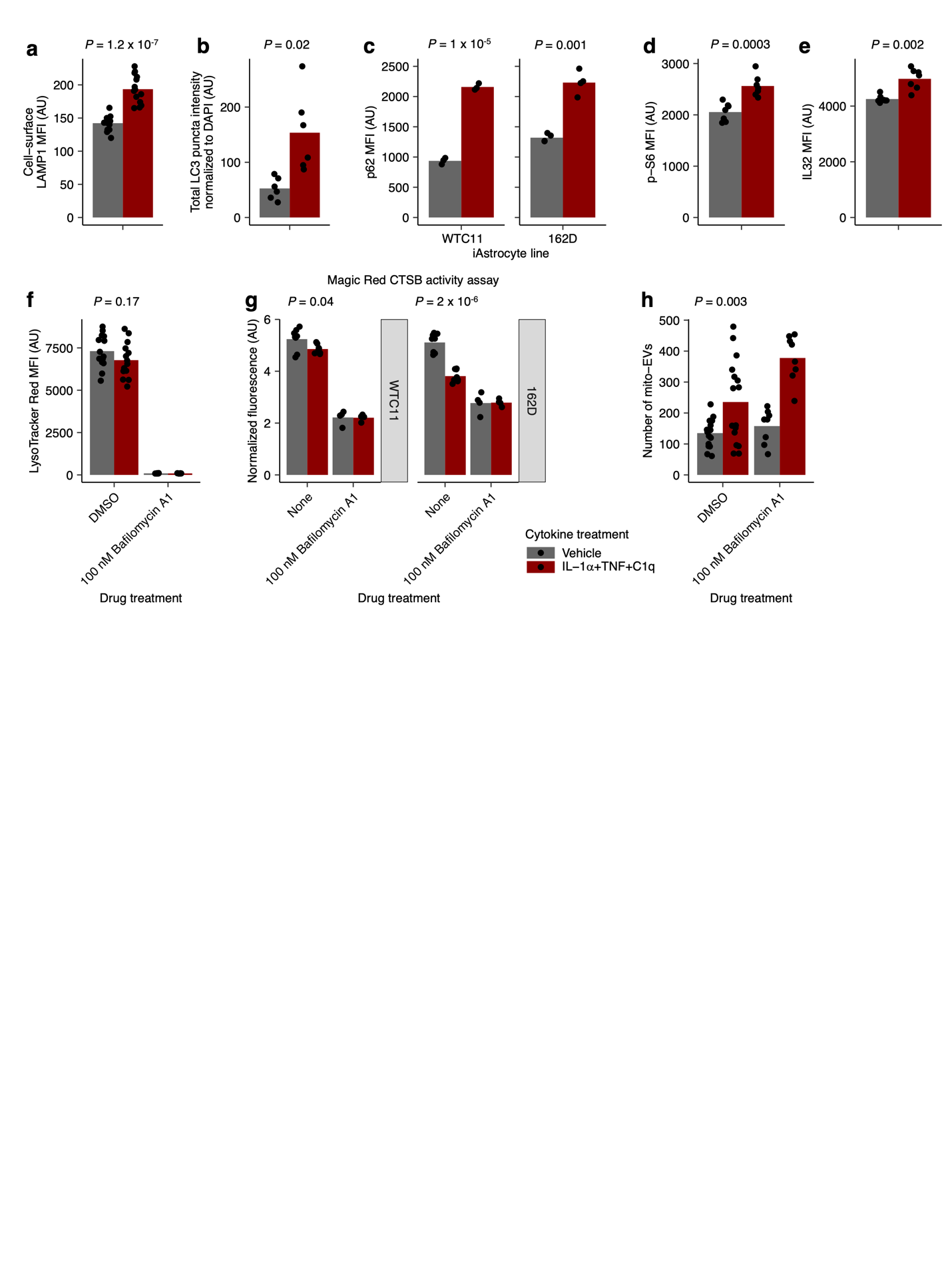
**

**Supplementary Figure 11 | Validation experiments using iAstrocytes derived from the 162D hiPSC line**. **a**, Median fluorescence intensity (MFI) of cell-surface LAMP1 measured by flow cytometry in iAstrocytes treated with vehicle vs. ITC; n = 14 wells per condition. **b**, Total intensity of LC3+ puncta measured by immunofluorescence imaging in iAstrocytes treated with vehicle vs. ITC; n = 6 wells per condition. **c**-**e**, MFI of p62 (**c**), phospho-S6 (**d**), or IL-32 (**e**) measured by flow cytometry in iAstrocytes treated with vehicle or ITC; n = 4 wells per condition for p62, n = 7 wells per condition for phospho-S6 and IL-32. **f**, MFI of LysoTracker staining in iAstrocytes treated with vehicle vs ITC with or without bafilomycin A1 co-treatment; n = 14 wells per condition for DMSO, n = 7 wells per condition for bafilomycin A1. **g**, Intracellular CTSB activity assayed using the Magic Red CSTB substrate, measured by fluorescence plate reader, in iAstrocytes treated with vehicle vs. ITC with or without bafilomycin A1 pre-treatment (3 hours) prior to the CTSB activity assay; n = 8 wells per condition in the absence of bafilomycin A1 pre-treatment, n = 4 wells per condition in the presence of bafilomycin A1 pre-treatment. **h**, Number of mito-EVs measured by flow cytometry in conditioned media from iAstrocytes treated with vehicle vs. ITC with or without bafilomycin A1 co-treatment; n = 16 wells per condition for DMSO, 8 wells per condition for bafilomycin A1. *P* values were calculated with the two-sided Student’s t test.
