# Supplementary material for: mTOR activation induces endolysosomal remodeling and nonclassical secretion of IL-32 via exosomes in inflammatory reactive astrocytes
Source: J Neuroinflammation. 2024 Aug 8;21:198. doi: 10.1186/s12974-024-03165-w (PMC11312292; doi:10.1186/s12974-024-03165-w)
Supplement: Supplementary file 8 — Additional file 8: Methods. [file 12974_2024_3165_MOESM8_ESM.docx]

**Methods**

**hiPSC culture**

Human iPSCs (male WTC11 background [82], female 162D background [83]) were cultured in Essential 8 (E8) Medium (ThermoFisher Scientific cat. no. A1517001) on BioLite Cell Culture Treated Dishes (ThermoFisher Scientific) coated with Growth Factor Reduced, Phenol Red-Free, LDEV-Free Matrigel Basement Membrane Matrix (Corning cat. no. 356231) diluted 1:100 in DMEM/F12 (ThermoFisher Scientific cat. no. 11330032). Essential 8 Medium was replaced daily. When hiPSC colonies demonstrated mature morphology, the hiPSCs were either clump passaged with EDTA for routine maintenance or dissociated to a near single-cell suspension with Accutase Cell Dissociation Reagent (ThermoFisher Scientific cat. no. A11105-01) for applications requiring cell counting. For clump passaging with EDTA, hiPSCs were washed with Dulbecco’s phosphate buffered saline (DPBS; Milipore Sigma cat. no. D8537) and then incubated with Versene (ThermoFisher Scientific cat. no. 15040066) for 5-7 min at room temperature; the Versene solution was then aspirated and replaced with E8 + 10 nM Y-27632 dihydrochloride ROCK inhibitor (Tocris cat. no. 125410); hiPSC colonies were then gently detached mechanically using a cell scraper, resuspended gently, and passaged at 1:10-1:30 dilution in E8 + Y-27632, with Y-27632 removed the next day. For near single-cell dissociation, hiPSCs were washed with DPBS, incubated with Accutase for 5-10 min at 37 °C, and then gently triturated with a P1000 pipette tip; the cell suspension was then diluted with PBS, collected into conical tubes, and spun down at 300 g for 3 min; hiPSCs were then resuspended in E8 + Y-27632, counted, and plated onto Matrigel-coated plates at the desired density in E8 + Y-27632; Y-27632 would be maintained until the hiPSC colonies reached the appropriate size (> ∼40 cells). Studies with hiPSCs at UCSF were approved by the The Human Gamete, Embryo and Stem Cell Research (GESCR) Committee. Informed consent was obtained from the human subject when the WTC11 line was originally derived.

**Generation of iAstrocytes**

iAstrocytes were generated as detailed in Leng *et al*. [9]. Briefly, we generated hiPSCs (male WTC11 background and female 162D background) used for iAstrocyte differentiation by inserting a CRISPRi machinery cassette (pC13N-dCas9-BFP-KRAB, Addgene plasmid no. 127968) into the CLYBL safe-harbor locus and a doxycycline-inducible *NFIA* and *SOX9* cDNA cassette into the AAVS1 safe-harbor locus using TALEN-based editing. After recovering a monoclonal CRISPRi-*NFIA*-*SOX9* line, we differentiated hiPSCs to the neuroectoderm lineage via an embryoid body (EB)-based neural induction protocol. After dissociating plating EB-derived cells, we selected and expanded those forming neural rosettes. We used fluorescence activated cell sorting (FACS) to enrich for pure CD133^+^/CD271^-^ neural progenitor cell (NPC) populations for expansion.

For iAstrocyte differentiation, these NPCs were re-plated on 1:200 Matrigel-coated dishes and cultured with ScienCell Astrocyte Media (ScienCell Research Laboratories cat. no. 1801) + 2 μg/mL doxycycline (Millipore Sigma cat. no. D9891) to initiate iAstrocyte differentiation. A full media change was completed every other day (maintaining doxycycline at 2 μg/mL) until NPCs reached confluency, at which time they were dissociated with Accutase and split 1:10 onto new Matrigel coated dishes (or cryopreserved) for continued differentiation and expansion. Expansion was continued until day 20 (d20) of differentiation, yielding iAstrocytes. See Leng *et al*. for full details of iAstrocyte hiPSC generation and differentiation protocols.

**Induction of inflammatory reactivity in iAstrocytes**

For all iAstrocyte experiments in this study, d20 iAstrocytes were plated at 20,000 cells/cm^2^ on 1:200 Matrigel-coated BioLite Cell Culture Treated Dishes (ThermoFisher Scientific) in ScienCell Astrocyte Media (without doxycycline) on d0. Full media changes were completed on d1, d3, and d5. On d5, media was supplemented with IL-1α (3 ng/mL; Peprotech cat. no. AF-200-01A), TNF (30 ng/mL; Peprotech cat. no. AF-300-01A), and C1q (400 ng/mL; Complement Technology cat. no. A099) to induce inflammatory reactivity according to Liddelow *et al*. [14]. All experiments were conducted 24 hours after cytokine treatment (day 6 after plating). For experiments involving addition of additional IFN-β, 5 ng/mL of IFN-β was added concurrently with ITC. For experiments involving addition of recombinant IL-32, 200 ng/mL of recombinant IL-32β (R&D Systems cat. no. 6769-IL) or IL-32γ (R&D Systems cat. no. 4690-IL/CF) was added concurrently with ITC.

**CRISPRi-mediated gene knockdown using lentiviral transduction of individual sgRNAs**

For knocking down target genes with a single sgRNA, we cloned CRISPRi sgRNAs into pMK1334 [84] (Addgene cat. no. 127965), as previously described [85]. For knocking down target genes using two sgRNAs to achieve greater degree of knockdown, we cloned sgRNAs into pMK1334 with an additional hU6 insert as described in Replogle *et al.* [86]. We generated lentivirus of the resulting constructs by co-transfecting them with 3^rd^ generation lentiviral packaging plasmids with TransIT-Lenti Transfection Reagent (Mirus cat. no. MIR6606) according to the manufacturer’s protocol. The next day, lentivirus was precipitated using Lentivirus Precipitation Solution (ALSTEM cat. no. VC150) according to the manufacturer’s protocol, resuspended in DPBS, and aliquoted and stored at -80 °C. iAstrocytes were transduced with lentivirus at the time of plating. When necessary, the functional titer of the lentivirus was determined by measuring BFP+ cells 48 hours after transducing iAstrocytes with a serial dilution of lentivirus. The protospacer sequences of the sgRNAs used in this study were selected from those generated in Horlbeck *et al.* [37] and are listed below (when two protospacers are listed, the sgRNA was cloned into pMK1334 using the Replogle *et al.* method):

Non-targeting controls (NTC):

GGAGTTAAGGCCTCGTCTAG, GTGCGGGGGCATGGCCCCGC

*MTOR*:

GGGACAGCGGGGAAGGCGGG

*RPTOR*:

GACCCCAGGTCCCAAGCCAC, GTGGGGGCCGCTAGGAAATG

*RICTOR*:

GGCGCAGGGCGGAATGACAG, GAGCGGGCTTACCTCGTACT

*TSC1*:

GGGCCTTGGCCCTTTCACGA

*VAMP7*:

GGCCAACTGCCCGCTCCCAG, GAGGGACGCGGGTCAGTGCA

*VAMP3*:

GCGGCAGCGGCGACGAGAGA, GGAAGCGAAGTTGGGACCGG

*SNAP23*:

GAACTCGGACACCCCAACAC, GTAGGGTGCAGCGCCAGGTC

*RAB27A/B*:

GACCCGAGCCAGGCGGGGAC (*RAB27A*), GCCCATCCGGAGCCACAGGT (*RAB27B*)

*IL32*:

GGAGCTGGGTCATCTCAGGT, GCGAAGGTGAGGACCCTCTG

**Generation and lentiviral transduction of reporter constructs**

To subclone the original FIRE-pHLy construct [24] (pLBR10, Ubc:FIRE-pHLy) to make pLBR11 (GfaABC1D:FIRE-pHLy), we excised the FIRE-pHLy cassette from pLBR10 and linearized the backbone vector (pKL20; pHIV gfaABC1D:mRuby:WPRE) with BamH1-HF (New England BioLabs, cat. no. R3136) and ClaI (New England BioLabs cat. no. R0197). We then ran these reactions on a 1% agarose gel, gel extracted the relevant fragments, and ligated them together with T4 DNA Ligase (New England BioLabs cat. no. M0202) per manufacturer’s protocol to generate pLBR11. Both pLBR11 (all FIRE-pHLy experiments), and GFP-LC3-RFP-LC3ΔG (reporter cassette from Addgene plasmid #84572) [25] reporters were transduced into iAstrocytes with lentivirus at low multiplicity of infection (MOI < 1) during plating to prevent high expression of exogenous lysosomal proteins.

**Immunofluorescence imaging**

For immunofluorescence imaging experiments involving iAstrocytes, iAstrocytes plated in Greiner μClear 96-well plates (Greiner Bio-One cat. no. 655087) were fixed with 4% paraformaldehyde (diluted from a 16% solution in DPBS; Electron Microscopy Sciences cat. no. 15710) for 15 min at RT. After washing three times with DPBS, we blocked and permeabilized cells with DPBS (Milipore Sigma cat. no. D8537) + 3% BSA (Milipore Sigma cat. no. A9647) + 0.1% Triton X-100 (Millipore Sigma cat. no. X100) for 30 min at RT. Primary antibodies against LC3 (1:50; Cell Signaling Technology cat. no. 2775), LAMP1 (1:50; Abcam cat. no. ab25630), LAMP2 (1:500; Abcam cat. no. ab25631), IL-32 (1:250; ProteinTech cat. no. 11079-1-AP), or CD63 (1:1420; BioLegend cat. no. 353039) were added to blocking buffer and incubated with iAstrocytes overnight at 4 °C. Afterwards, the samples were washed with DPBS three times, incubated with pre-adsorbed secondary antibodies (1:500 goat anti-mouse IgG Alexa Fluor 488, 1:500 goat anti-rabbit IgG Alexa Fluor 555; Abcam cat. no. ab150117 and ab150086) for 1 hour at RT, washed three times with DPBS, incubated with 1 μg/mL Hoechst 33342 (ThermoFisher Scientific cat. no. H3570) for 10 minutes and then washed two additional times before imaging on an IN Cell Analyzer 6000, using a 60X 0.7 NA objective, 2×2 binning, 100-400 ms exposure, an aperture width of ∼1 Airy unit, and 9-16 fields per well, or on an ImageExpress Confocal HT.ai (Molecular Devices), using an APO 20x 0.95 NA water-immersion objective (Nikon), 89 North LDI lasers 405 nm, 475 nm, and 555 nm with emission filters 452/32 nm 520/428 nm and 598/25 nm for Hoechst 333342, AlexaFluor 488 and 555, respectively, 1x1 binning (325 x 325 nm pixel size), 100-400 ms exposure, 60 μm pinhole spinning disk, and 12 fields per well captured on a Zyla 4.5 sCMOS camera (Andor).

For immunofluorescence imaging experiments involving HIE samples, all human HIE tissue was collected with informed consent and in accordance with guidelines established by the UCSF Committee on Human Research (H11170-19113-07), as previously described [87]. Immediately after procurement, all brains were immersed in PBS with 4% paraformaldehyde for 3 days. On day 3, the brain was cut in the coronal plane at the level of the mammillary body and immersed in fresh 4% paraformaldehyde and PBS for an additional 3 days. After fixation, all tissue samples were equilibrated in PBS with 30% sucrose for at least 2 days. After sucrose equilibration, tissue was placed into molds and embedded with OCT medium for 30 minutes at room temperature, followed by freezing in dry-ice-chilled ethanol. UCSF neuropathology staff performed brain dissection and its evaluation. The diagnosis of HIE requires clinical and pathological correlation; no widely accepted diagnostic criteria are present for the pathological diagnosis of HIE. HIE cases showed consistent evidence of diffuse white matter gliosis, as evaluated by the qualitative increase in the number of GFAP^+^ cells in addition to the increased intensity of GFAP staining. For immunostaining of reactive astrocyte markers, tissue slides were bleached with UV overnight, rinsed with PBS for 10 minutes and incubated with blocking solution (10% normal goat serum + 0.2% Triton X-100 in PBS) for 1 hour at room temperature and then incubated with the following primary antibodies overnight at 4 °C: chicken anti-GFAP (1:500 dilution; BioLegend cat. no. 829401), mouse anti-NeuN (1:500 dilution; Millipore Sigma cat. no. MAB377), mouse anti-OLIG1 (1:200; Millipore Sigma cat. no. MAB2417), or rabbit anti IL32 (1:200 dilution; ProteinTech cat. no. 11079-1-AP). Afterwards, the samples were rinsed three times with PBS + 0.2% Triton X-100 for 10 minutes each time and then incubated with the appropriate secondary antibodies for 1 hour at room temperature in the dark.

**LysoTracker staining and imaging**

iAstrocytes were washed with DPBS and incubated with a 50 nM solution of LysoTracker Green DND-26 (ThermoFisher Scientific cat. no. L7526) or LysoTracker Red DND-99 (ThermoFisher Scientific cat. no. L7528) in DPBS for 5 minutes at 37°C, washed two times with DPBS, and processed for downstream experiments. For imaging, iAstrocytes were imaged at 37°C and 5% CO_2_ using an IN Cell Analyzer 6000 as above. For flow cytometry (CRISPRi screens), iAstrocytes were dissociated with Accutase for 10 mins at 37°C after LysoTracker staining, and resuspended in FACS buffer (DPBS + 1% BSA + 2 mM EDTA (Milipore Sigma cat. no. 324506)).

**CTSB activity assay**

Intracellular CTSB activity was measured using the Cathepsin B Assay Kit (Magic Red) from Abcam (ab270772) following the manufacturer’s instructions. Vehicle vs ITC-treated iAstrocytes plated on a Matrigel-coated Greiner μClear 96-well plates (Greiner Bio-One cat. no. 655087) were pre-treated with vehicle or 100 nM Bafilomycin A1 for 3 hours and then incubated with 7 uL of 1:10 diluted Magic Red CTSB substrate per well for 1.5 hours. Fluorescence from cleaved Magic Red CTSB substrate was then measured on a SpectraMax M5 fluorescence plate reader with excitation set to 592 nm and emission set to 628 nm with auto-cutoff enabled. iAstrocytes were then incubated with 10 μg/mL Hoechst for 15 min at 37°C and then read on the plate reader with excitation set to 359 nm and emission set to 461 nm with auto-cutoff enabled. CTSB activity normalized to cell number was calculated by dividing the Magic Red fluorescence by the Hoechst fluorescence.

**Total internal reflection fluorescence microscopy**

iAstrocytes were plated at 20,000 cells/cm2 in 35 mm imaging dishes (Ibidi, cat. no. 81156) and simultaneously transduced with pLBR11 (GfaABC1D:FIRE-pHLy). Prior to imaging, the conditioned media of iAstrocytes was collected. iAstrocytes were then stained with LysoTracker as detailed above and re-incubated with the collected conditioned media for the duration of imaging. TIRF was performed on a Nikon Ti-E inverted microscope equipped with a Nikon TIRF illuminator, Nikon motorized stage with piezo Z inset, Andor iXon Ultra DU897 camera, and run by NIS-Elements (v5.20.00 build 1423). iAstrocytes were imaged using an Apo TIRF 100x/1.49 oil (DIC N2/ 100X I) objective, 488nm and 561nm lasers (Agilent MLC400 monolithic laser combiner) and Sutter Lambda 10-B with ET525/50m and ET600/60m emission filters (Chroma), respectively, for 120 seconds with 100 ms exposure (per channel).

**Antibody staining for flow cytometry**

To measure cell-surface protein levels (i.e. LAMP1, TFRC), iAstrocytes were dissociated with Accutase for 10 mins at 37 °C, diluted with ice-cold DPBS + 1% BSA, and spun down at 300 x g for 5 minutes at 4 °C. After aspirating the supernatant, all cells were incubated in 1:20 Fc block (BD Bioscience cat. no. 564220) diluted in DPBS + 3% BSA for 10 minutes on ice, followed by the addition of 1:50 primary antibodies against LAMP1 (AF488- or BV605-conjugated; BioLegend cat. no. 328610 and 328634) for an additional 30 minute incubation on ice in the dark. iAstrocytes were then washed with ice-cold DPBS + 1% BSA, spun down at 300 x g for 5 mins at 4 °C, and resuspended in DPBS + 1% BSA for flow cytometry. When astrocytes were not transduced with sgRNAs, DAPI (ThermoFisher Scientific cat. no. D1306) was added at 0.4 mg/mL to assess cell viability; when astrocytes were transduced with sgRNAs, TO-PRO-3 (ThermoFisher Scientific cat. no. T3605) was added at 1 μM.

For intracellular staining, dissociated iAstrocytes were fixed with 2% paraformaldehyde for 10 min at RT, washed twice with DPBS + 0.5% Tween 20 (Millipore Sigma cat. no. P9461), incubated with unconjugated primary antibodies against C3 (1:500; ThermoFisher Scientific cat. no. PA1-29715), VCAM1 (1:250; BioLegend cat. no. 305802), phospho-S6 (1:200; Cell Signaling Technology cat. no. 2211), phospho-4E-BP1 (1:200; Cell Signaling Technology cat. no. 2855), p62 (1:200; Cell Signaling Technology cat. no. 88588), or IL-32 (1:250; ProteinTech cat. no. 11079-1-AP) for 20 min at RT, washed with DPBS + 0.5% Tween 20, incubated with conjugated secondary antibodies (1:1000, Donkey anti mouse IgG AlexaFluor 488 conjugated, ThermoFisher Scientific cat. no. A-21202; 1:1000 Donkey anti rabbit IgG AlexaFluor 555 conjugated, ThermoFisher Scientific cat. no. A-31572; 1:1000 Donkey anti goat IgG AlexaFluor 647 conjugated, ThermoFisher Scientific cat. no. A-21447) for 20 min at RT, washed with DPBS + 0.5% Tween 20, and then resuspended in DPBS + 0.5% Tween 20 for flow cytometry.

**Pooled CRISPRi screening**

CRISPRi screens were conducted as described in Leng *et al*. [9]. Both LysoTracker and LAMP1 screens were conducted with the H1 (i.e. “druggable genome”) sgRNA sub-library from our next-generation CRISPRi library [37]. We generated lentivirus for the pooled H1 sgRNA library as previously described [84]. For each screen (i.e. LysoTracker or cell-surface LAMP1, vehicle or ITC), iAstrocytes were plated at 20,000 cells/cm^2^ onto 4 Matrigel-coated 15-cm dishes, transduced with the H1 lentiviral sgRNA library with >70% transduction efficiency, treated with vehicle control or IL-1α+TNF+C1q for 24 hours, stained for LysoTracker or cell-surface LAMP1, and then sorted into LysoTracker high vs. low or cell-surface LAMP1 high vs. low (top and bottom 35% of cells on fluorescence histogram) populations. iAstrocytes were sorted with a BD FACSAria Fusion cell sorter at 5,000-10,000 events per second, and then pelleted via centrifugation at 300 x g for 10 minutes for genomic DNA extraction. sgRNA abundances were then measured using next-generation sequencing as previously described [84]. LysoTracker screens were performed with two experimental replicates per condition, while LAMP1 screens were done with one replicate. Results from all CRISPRi screens in this study can be interactively explored at [www.crisprbrain.org](http://www.crisprbrain.org/) [88].

**Extracellular vesicle isolation**

For all experiments involving isolation of extracellular vesicles (EVs) from astrocyte conditioned media, astrocytes were treated with ScienCell Astrocyte media made using exosome-depleted FBS (ThermoFisher Scientific cat. no. A2720803). Extracellular vesicles were isolated from 10 mL of condition media from iAstrocytes plated onto 10 cm dishes using differential ultracentrifugation as described in Patel *et al.* [89]. All spins were done at 4 °C. Briefly, conditioned media was spun down at 300 g for 10 min, then the supernatant was spun down at 2000 g for 30 min, then the supernatant was spun down at 15,600 g for 30 min, then the supernatant was spun down at 120,000 g for 2 hours, and finally the pellet (not visible) was resuspended in 100 μL of DPBS. The spins at 15,600 g and 120,000 g were done using a SW 41 Ti rotor with 13.2 mL UltraClear tubes (Beckman Coulter cat. no. 344059) in a Beckman Coulter Optima XE ultracentrifuge. To increase exosome yield for western blotting against certain targets shown in Fig. 5a (Hsc70, Caveolin-1, IL-32), 10 nM bafilomycin A1 was added together with vehicle control or ITC. For IL-32, bands were present on blots using samples treated with or without bafilomycin A1 (see raw blot image files in Additional file 1).

**Measurement of EV size distribution**

The size distribution of EVs isolated above was measured via nanoparticle tracking analysis using the NanoSight 3000 following the manufacturer’s instructions. 100 μL of each resuspended EV sample was diluted to final volume of 500 μL for analysis on the Nanosight 3000.

**Western blots**

iAstrocytes were dissociated with Accutase for 10 mins at 37°C, spun down at 300 x g for 5 mins, washed with ice-cold DPBS, lysed in RIPA buffer (Thermo Fisher Scientific, cat. no. 89900) with protease (Roche, cat. no. 04693159001) and phosphatase inhibitors (Sigma-Aldrich, cat. no. PHOSS-RO), and incubated on ice for 45 minutes with brief vortexing every 15 minutes. Samples were centrifuged at 18,000 x g for 20 mins at 4°C to pellet insoluble components of lysate. After the total protein was quantified using the Pierce BCA Protein Assay Kit (ThermoFisher Scientific, cat. no. 23225) in the supernatant, each sample was diluted to the same concentration, and denatured with NuPAGE LDS Sample Buffer (4X) (ThermoFisher Scientific, cat. no. NP0007) and reduced with DTT.

Equivalent protein amounts were run on 10% NuPAGE Bis-Tris gels (ThermoFisher Scientific, cat. no. NP0301) and transferred to nitrocellulose membranes (Bio-Rad, 1704271). For blots shown in Fig. 1f-h, Fig. 1l, and Fig. 3g, 20-40 μg of protein was loaded; for blots shown in Fig. 5a,c, 1-2 μg of protein was loaded. We performed ponceau staining (Milipore Sigma, cat. no. P7170) to validate equivalent loading between lanes. Membranes were blocked with Intercept blocking buffer (LI-COR cat. no. 927-60001) on a shaker, after which they were incubated with primary antibodies against mTOR (1:1000, Cell Signaling Technology cat. no. 2972), phospho-S6 (1:500; Cell Signaling Technology cat. no. 2211), S6 (1:1000; Cell Signaling Technology cat. no. 2217), phospho-ULK1 (1:500; Cell Signaling Technology cat. no. 14202), phospho-Akt (1:500; Cell Signaling Technology cat. no. 4060), pan-Akt (1:1000; Cell Signaling Technology cat. 2920), phospho-4E-BP1 (1:1000; Cell Signaling Technology cat. no. 2855), LAMP1 (1:1000; Abcam cat. no. ab25630), LAMP2 (1:1000; Abcam cat. no. ab25631), CTSD (1 ug/mL, R&D Systems cat. no. AF1014), β-actin (1:1000; Cell Signaling Technology cat. no. 4970), EGFP (1:200; Santa Cruz Biotechnology cat. no. sc-9996), LC3 (1:1000; Cell Signaling Technology cat. no. 2775), CD81 (1:500; BioLegend cat. no. 349502), CD63 (1:5680; BioLegend cat. no. 353039), Apo-AI (1:200; Santa Cruz Biotechnology cat. no. sc-376818), GAPDH (1:200; Santa Cruz Biotechnology cat. no. sc-47724), Hsc70 (1:200; Santa Cruz Biotechnology cat. no. sc-7298), TSG101 (1:500; BioLegend cat. no. 934301), ALIX (1:500; BioLegend cat. no. 634501), or Caveolin-1 (1:1000; Cell Signaling Technology cat. no. 3267) at 4°C overnight on a shaker. Blots were washed with TBS and incubated with secondary antibodies goat anti-mouse IRDye 800CW (1:10,000; LI-COR cat. no. 926-32210) and goat anti-rabbit IRDye 680RD (1:10,000; LI-COR cat. no. 926-68071) in blocking buffer (see above) for 1 hour at RT on a shaker. Afterwards, blots were washed with TBS, and imaged using an Odyssey Fc Imaging system (LI-COR, cat. no. 2800). To re-probe blots, we incubated membranes with 1X NewBlot Nitro Stripping Buffer (LI-COR, cat. no. 928-40030) for 5 minutes at RT on a shaker before re-blocking and probing as above. Immunoblot bands were quantified with LI-COR ImageStudio software. For blotting against CD63 and CD81, reducing agent was omitted from the sample buffer since the antibodies used here recognize epitopes containing disulfide bonds.

Source data for immunoblots is provided in Additional file 1.

**mito-EV staining for flow cytometry**

Conditioned media from iAstrocytes was spun down at 300 g to remove dead cells and then incubated with 10 μg/mL Hoechst, 5 nM TMRM (ThermoFisher Scientific cat. no. T668), and 50 nM MitoTracker Green (ThermoFisher Scientific cat. no. M7514) for 5 min at 37 °C. After incubation, the conditioned media was analyzed directly with flow cytometry.

**IL-32 or CTSB immunoassays**

IL-32 concentration was measured using an ELISA kit (R&D Systems cat no. DY3040-05) following the manufacturer’s instructions. For measurement of IL-32 concentration in iAstrocyte conditioned media, the conditioned media was first spun down at 300 g to remove dead cells, then 50 μL of conditioned media + 25 μL of RIPA buffer + 25 μL of 1% BSA in DPBS was added per well, with the same fraction of RIPA and diluent added to the IL32 standards. For measurement of IL-32 concentration in the exosome fraction vs. supernatant, 75 μL of sample + 25 μL of RIPA buffer was added per well, with the same fraction of RIPA and diluent added to the IL32 standards. CTSB concentration was measured via an electrochemiluminescence immunoassay (Mesoscale Discoveries cat. no. K151ACAR-2) following the manufacturer’s instructions.

**Whole-cell proteomics sample preparation**

iAstrocytes were washed and collected (detached gently using a cell scraper) in ice-cold DPBS, spun down at 300 x g for 5 mins at 4 °C, and stored at -80 °C. Cell pellets were lysed in 2.5% sodium dodecyl sulfate (SDS) 50mM Tris pH 8 and heated to 95 °C for 5min, then subjected to probe sonication. Lysates were centrifuged at 14000g for 15 min before protein quantification using BCA assay (Pierce, Thermo Fisher Scientific) according to manufacturer’s instructions. Protein lysates were acetone precipitated by adding 5 volumes of ice-cold acetone, vortexed, incubated at -20 °C for 1 hr and pelleted by centrifugation at 21000g and 4 °C for 15 min. Protein pellets were resuspended in 1% sodium deoxycholate (SDC) 50 mM EPPS pH 8.5 and reduced by incubating at 37 °C for 20 min with rotation in the presence of 5mM DTT. Protein was then alkylated by incubating in the dark at 37 °C for 20 min in the presence of 25mM iodoacetamide before digestion overnight with LysC at an enzyme-to-protein ratio of 1:50 followed by 3 hr digestion with trypsin at an enzyme-to-protein ratio of 1:50. Digestion was stopped by acidification to 1% trifluoroacetic acid (TFA) and placed on ice for 10min to promote precipitation of SDC. The SDC was then pelleted and removed by centrifugation at 21000g for 10min. The supernatant containing acidified peptides were then de-salted on in-house made Stagetips [90] packed with polystyrene divinyl benzene reverse phase sulphonate solid phase extraction material (CDS Empore, Fisher Scientific) as previously published [90]. Stagetips were prepared by activating with 100% methanol, conditioning with 80% acetonitrile containing 0.1% TFA, equilibrated with 0.2% TFA, followed by sample loading, washing twice with 99% isopropanol containing 0.1% TFA, washing twice with 0.2% TFA and once with 0.1% FA. Peptides were eluted with 60% acetonitrile (ACN) containing 0.5% ammonium hydroxide. The desalted peptides were then flash frozen and dried by centrifugal evaporation.

**Endolysosomal proteomics sample preparation**

pLBR09 (gfaABC1D: LAMP1-XTEN80-mEGFP-3XHA) used for lysosome immunoprecipitation was generated by subcloning pLBR08 (EF1α: LAMP1-XTEN80-mEGFP-3XHA). Briefly, pLBR08 was generated via Gibson assembly of PCR-amplified LAMP1 cDNA (from mTagRFP-T-Lysosomes-20 acquired from Nikon Imaging Center at UCSF, Addgene plasmid #58022) and PCR-amplified XTEN80-mEGFP-3xHA immunoprecipitation tag with a linearized backbone generated from ClaI and BspDI (New England BioLabs cat. no. R0557) digestion of pKL017 (pHIV EF1a:Clover:WPRE). pLBR09 was then generated by linearizing pKL020 (pHIV gfaABC1D:mRuby:WPRE) and excising the LAMP1-XTEN80-mEGFP-3XHA cassette of pLBR08 via restriction digest with XbaI (New England Biolabs cat. no. R0145) and ClaI. The resulting fragments were run on a 1% agarose gel and gel extracted (Zymoclean Gel DNA Recovery Kit, cat. no. D4007). These fragments were ligated together with T4 DNA Ligase per manufacturer protocol. iAstrocytes were plated at 20,000 cells/cm^2^ per experimental replicate and transduced with pLBR09 lentivirus during plating. We used three replicates each for vehicle and ITC conditions.

Lysosome immunoprecipitation was performed as previously described [91]. Briefly, iAstrocytes were washed and collected (detached gently using a cell scraper) in ice-cold DPBS, spun down at 300 x g for 5 mins at 4 °C. We then resuspended the cell pellet in homogenization buffer (25 mM Tris HCl pH 7.5, 50 mM sucrose, 0.5 mM MgCl_2_, 0.2 mM EGTA with protease inhibitors). The iAstrocyte cell suspension was mechanically lysed with a 23G syringe, immediately transferred to isotonic buffer (2.5M sucrose, 0.2 mM EGTA, 0.5 mM MgCl_2_) and spun at 3000 x g for 10 mins at 4 °C. The supernatant was transferred to anti-HA magnetic beads (Thermo Scientific cat. no. PI88836) for the pulldown of intact lysosomes, with the final resuspension in 1X urea buffer (2M Urea, 1 mM DTT, 12.5 mM Tris). On-bead digest was performed by sequentially incubating the resulting protein samples with 5 mM (final concentration) IAA at 22°C and 225 rpm for 30 mins, 0.5 μg LysC at 22°C and 225 rpm overnight, 1 μg of trypsin at 22°C and 225 rpm for 4 hours, and 1% (final concentration) TFA. Acidified peptides were then de-salted according to the same protocol as whole-cell proteome samples. For western blotting, NuPAGE LDS Sample Buffer (4X) was added to samples collected prior to the on-bead digestion. See Western blots section for further details.

**LC-MS Analysis**

Peptides were resuspended in 2% ACN with 0.1% TFA before loading onto a 25 cm x 75 µm ID, 1.6 µm C18 column (IonOpticks) maintained at 40°C. Peptides were separated with an EASY-nLC 1200 system (Thermo Fisher Scientific, San Jose, CA) at a flow rate of 300 nL min^-1^ using a binary buffer system of 0.1% FA (buffer A) and 80% acetonitrile with 0.1% FA (buffer B) in a two-step gradient. The following was performed for whole cell proteome samples, 3% to 27% B in 105 min and from 27% to 40% B in 15min. For lysosome-specific samples, 3% to 27% B in 52.5 min and from 27% to 40% B in 14.5 min. All samples were analyzed on a Fusion Lumos mass spectrometer (Thermo Fisher Scientific, San Jose, CA) equipped with a nanoFlex ESI source operated at 1550 volts, RF lens set to 30%, operated in data dependent acquisition mode with a duty cycle time of 1 sec. Full MS scans were acquired with a m/z scan range of 375-1500 m/z in the Orbitrap mass analyzer (FTMS) with a resolution of 240k for whole cell proteome and 120k for lysosome-specific samples. Selected precursor ions were subjected to fragmentation using higher-energy collisional dissociation (HCD) with a quadrupole isolation window of 0.7 m/z, and normalized collision energy of 31%. HCD fragments were analyzed in the Ion Trap mass analyzer (ITMS) set to Turbo scan rate. Fragmented ions were dynamically excluded from further selection for a period of 45 sec for whole cell proteome and 60 sec for lysosome-specific samples. The AGC target was set to 1,000,000 and 10,000 for full FTMS and ITMS scans, respectively. The maximum injection time was set to Auto for both full FTMS and ITMS scans.

**Drug treatments**

Vacuolin-1 (Milipore Sigma, cat. no. 673000), bafilomycin A1 (Milipore Sigma, cat. no. B1793), rapamycin (Milipore Sigma, cat. no. 553210), PP242 (Milipore Sigma, cat. no. 475988), RapaLink-1 (MedChemExpress cat. no. HY-111373), PI4KIII beta inhibitor 3 (MedChemExpress cat. no. HY-15679), and PI-273 (MedChemExpress cat. no. HY-103489) were resuspended in DMSO per manufacturer’s instructions, aliquoted, and stored at -80°C. All compounds were added to iAstrocytes alongside vehicle or ITC treatments (i.e. 24 hour treatments) with the exception of using bafilomycin A1 for measurement of autophagic flux, which was added to astrocytes for 4 hours before sample collection.

**Data analysis**

*Analysis of published RNA-seq datasets*

We used BioJupies [92] to reanalyze bulk RNA-seq data and obtain differentially expressed genes from Perriot *et al*. [93] (GSE120411; hiPSC-derived astrocytes treated with IL1β and TNF) and Barbar *et al*. [19] (syn21861229; CD49f+ astrocytes sorted from cerebral organoids treated with vehicle control or IL-1α+TNF+C1q).

*Overlap analysis of differentially expressed genes (DEGs)*

Human or rodent astrocyte DEGs from various diseases or disease models were extracted manually from the supplementary information provided on the respective publications or shared by the corresponding authors upon request; see Table S3 (Additional file 5) for a list of all publications and datasets used for the analysis. GO Cellular Component terms relating to the endolysosomal system and biological pathway terms related to mTOR signaling were downloaded from Enrichr [94-96]. Overlap between astrocyte DEGs and the gene sets of interest was assessed and visualized using the R package GeneOverlap (v1.38.0); Fisher’s exact test *P* values were adjusted for multiple testing using the Benjamini-Hochberg procedure.

*Proteomics*

Raw files were searched against the human reference proteome (Swiss-Prot, downloaded on 20^th^ of April 2020, refer to PRIDE upload for full sequence) along with the default common contaminants list with enzyme specificity set to trypsin within MaxQuant version 2.0.1.0 using standard settings with match between runs and the MaxLFQ algorithm activated. Whole cell proteome (raw data file names begin with ‘Proteome’) and lysosome-specific (raw data file names begin with ‘LAMP1’) raw files were set as separate parameter groups to ensure that the MaxLFQ algorithm was performed only within each sample set. The resulting proteinGroups text file was then uploaded to Perseus version 1.6.7.0 where all reverse sequences, potential contaminants and proteins only identified by site were filtered out of the dataset. All protein abundance values were normalized by Log 2 transformation. The lysosome-specific raw data files were evaluated separate from whole cell proteomic files. Search results from lysosomal raw data files were filtered to include proteins that were quantified in at least two out of three biological replicates within at least the ITC (raw data file names include ITC) or vehicle (raw data file names include WT) triplicate set. Missing values were then imputed within each replicate by replacing from the normal distribution using default settings. A two sample students t-test was performed between the ITC treated and vehicle triplicates, with permutation-based FDR using default settings. The resulting T-test difference and significance were used to generate the lysosome-specific volcano plot.

*Pathway enrichment analysis*

We used Enrichr [94-96] to perform enrichment analysis of gene lists.

*CRISPRi screening analysis*

We analyzed next generation sequencing reads generated from our CRISPRi screens as previously described [84]. The “gene score” was calculated as -log_10_(*P* value) times the phenotype score.

*Pseudobulk analysis of snRNA-seq data from Macnair* et al.

The processed feature-barcode count matrix from Macnair *et al.* [62] was downloaded from <https://zenodo.org/records/8338963> [97] and loaded into R as a Seurat object. Seurat::AggregateExpression was then used to combine raw counts across cells from each cell type for each sample. DESeq2::estimateSizeFactors was then used to normalize the counts, and then the normalized counts added a pseudocount of 1 followed by transformation to log_2_ scale. IL-32 log-normalized counts were then plotted across samples derived from different multiple sclerosis lesions. *P* values for differences in IL-32 log-normalized counts were calculated by linear regression in R using lm() without adjustment for clinical covariates.

*Flow cytometry*

Data from flow cytometry experiments were analyzed using FlowJo (version 10.7.1). Live cells were gated by plotting SSC-A vs. FSC-A and then single cells were gated by plotting FSC-H vs. FSC-A. For experiments involving CRISPRi knockdown, analysis was restricted to sgRNA-transduced cells (gating on the histogram of BFP fluorescence values). For antibody staining experiments where median fluorescence intensity (MFI) values were reported, the average MFI of unstained control samples were subtracted from the MFI of stained samples.

*Fluorescence imaging analysis*

We used CellProfiler (v3.15) [98] to quantify iAstrocyte immunostaining imaging data. The total image intensity of LAMP1, LAMP2, LC3, and LysoTracker signals were quantified after first thresholding images relative to either a no-primary antibody control (immunofluorescence—LAMP1, LAMP2, LC3) or no-dye control (LysoTracker). The total image intensity of LAMP1, LAMP2, and LC3 was then divided by the total intensity of Hoechst to correct for variation in cell number. All frames collected were averaged to return a single quantification per well, which we considered as a single data point for statistical tests.

*Statistics and reproducibility*

Sample sizes were determined by referencing existing studies in the field. Major findings were validated using independent samples and orthogonal approaches. Numbers of replicates are listed in each figure. Randomization was not relevant to our study because no animals or human subjects were involved. Statistics were computed in RStudio (version 4.0.5). Normality of data distributions were tested where appropriate (Shapiro-Wilk test using the function shapiro.test). Simple pairwise tests were conducted using t.test for normally distributed data or wilcox.test for non-normally distributed data. For instances involving multiple comparisons of normally distributed data, linear regression using the function lm was used to compute *P* values. For experiments with factorial design, linear regression including interaction terms (equivalent to multi-way ANOVA) was performed using the function lm. For percentage data or data ranged from 0 to 1, beta regression was used to calculated *P* values using the R package betareg (v3.1-4), with logit linking function and the bias-corrected maximum likelihood estimator. Correction for multiple testing was performed where appropriate.
